# New Synthetic Analogs of Natural 5Z,9Z-Dienoic Acids—Hybrid Molecules Based on Oleanolic Acid: Synthesis and Study of Antitumor Activity

**DOI:** 10.3390/cancers16233893

**Published:** 2024-11-21

**Authors:** Regina A. Tuktarova, Lilya U. Dzhemileva, Usein M. Dzhemilev, Vladimir A. D’yakonov

**Affiliations:** 1 N.D. Zelinsky Institute of Organic Chemistry, Russian Academy of Sciences, Leninsky Prospect 47, Moscow 119991, Russia; regina-tuktarova@yandex.ru (R.A.T.); dzhemilev@anrb.ru (U.M.D.); 2 Chemical Engineering Center, ITMO University, Kronverksky Prospekt 49, Saint Petersburg 191002, Russia

**Keywords:** oleanolic acids, 5,9-dienoic acid, hybrid molecules, cytotoxicity, anticancer activity, apoptosis, cell cycle, genotoxicity

## Abstract

**Objectives**: A series of synthetic analogs of natural (5Z,9Z)-diene acids were synthesized for the first time in the form of hybrid molecules containing an oleanolic acid fragment. This fragment was simultaneously linked by an amide bond to various hetero- and carbocyclic amines and a complex ester bond to (5Z,9Z)-tetradeca-5,9-dienecarboxylic acid, which was synthesized by a new reaction of Ti-catalyzed homocyclomagnification of 1,2-dienes. **Results**: Among the synthesized hybrids, the highest cytotoxic activity was observed for compound **9a** in the series of Jurkat, K562, U937, and HEK293, with IC50 values of 4.5; 3.1; 2.8; and 26.17 μM/L, respectively. Furthermore, the synthesized compound **9a** has been observed to induce apoptosis and exhibit genotoxicity in Jurkat culture, which suggests that it may be a promising candidate for further investigation as an antitumor agent.

## Simple Summary

It is of great importance to consider the genotoxicity of drug compounds when designing various compounds with potential biological activity. The synthesis of hybrid molecules comprising natural (5Z,9Z)-diene acids and oleanolic acid was achieved for the first time through the implementation of a novel reaction involving Ti-catalyzed homocyclomagnetization of 1,2-dienes. The synthesized hybrids have been observed to exhibit cytotoxicity against Jurkat, K562, U937, and HEK293 cell lines, and have also demonstrated genotoxicity in Jurkat cells. It seems probable that the principal mechanism of action of these molecules is the inhibition of topoisomerase I, which is of great significance in the context of the potential development of anticancer drugs.

## 1. Introduction

Oleanolic acid (OA) is one of the most prevalent pentacyclic triterpenoids in nature. It is pervasively distributed throughout the plant kingdom, where it is frequently encountered in both its free form and as a saponin triterpenoid, an aglycone bonded to one or more sugar groups. OA is a common constituent of numerous foods and plant materials and is also an indispensable component of the human diet [1].

**Scheme 1:**
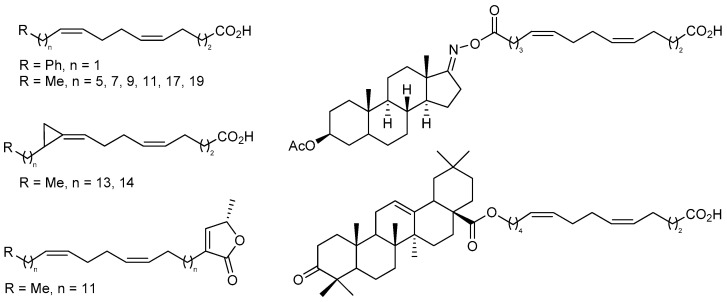
Some natural and synthetic inhibitors of topoisomerase I containing a 1Z,5Z-diene fragment.

Oleanolic acid and other triterpenes with similar chemical structures possess a diverse range of pharmacological properties [2]. Given its intricate and multifaceted mechanisms, it exerts a favorable impact on lipid metabolism in individuals with diabetes mellitus and metabolic syndrome. Additionally, oleanolic acid enhances insulin sensitivity in tissues, preserves β-cell viability, and serves as a protective agent against complications associated with diabetes. There is evidence to suggest that it has antiviral, antibacterial, antifungal, anticarcinogenic, antidiabetic, anti-inflammatory, hepatoprotective, hypolipidemic, and anti-atherosclerotic effects [3]. A synthetic triterpenoid, oleanolic acid, was identified as a potent inhibitor of inflammation. It has been demonstrated to induce the production of interferon-γ, inducible nitric oxide synthase (iNOS), and cyclooxygenase-2 in mouse macrophages. These compounds are considered to be highly potent inducers of the immune response, for example by decreasing NADH-ubiquinone oxidoreductase and hemoxygenase-1, and they protect cells from oxidative and electrophilic stress [4]. Over the past decade, our research group, in collaboration with other groups, has demonstrated that natural and synthetic fatty dienoic acids containing a 1Z,5Z-diene fragment in their structure exhibit a diverse range of biological activities, including antitumor, antibacterial, antiviral, and other effects (Scheme 1) [5,6,7,8,9,10,11]. In this series of studies, highly effective inhibitors of human topoisomerase I and II were identified among derivatives of these acids, and the mechanisms of action of these compounds in the cell were elucidated. Additionally, various variants of inhibitors were proposed [12,13,14,15,16,17,18,19].

The objective is to create unique molecules with multitarget action, particularly pronounced in hemoblastosis. In addition to inhibiting topoisomerase I by a pharmacophore containing a 5Z,9Z-diene system, the steroid component is likely to have a powerful immunoregulatory effect, including the inhibition of lymphoid cell activity, the maturation and differentiation of both T- and B-subpopulations of lymphocytes, and the induction of lymphoid cell apoptosis [20]. These characteristics are of significant importance in the treatment of T-cell hemoblastosis.

The objective of this study is to propose the hypothesis that the synthesis of hybrid molecules containing an oleanolic acid fragment and a natural (5Z,9Z)-diene acid should result in the development of compounds with sufficiently high cytotoxicity against tumor lines, a high-penetrating effect inside the cell, the inhibition of one of the key cell cycle enzymes, human topoisomerase I, and the activation of a number of signaling pathways responsible for cell growth and proliferation. This strategy has previously demonstrated high efficiency in the synthesis of (5Z,9Z)-diene acids containing a steroidal fragment [13,15,16,17,18].

Concurrently, a variety of hetero- and carbocyclic amines were incorporated into the hybrid molecules described above through amide bonding.

## 2. Materials and Methods

### 2.1. Chemistry

Oleanolic acid, heterocyclic amines, 4-dimethylaminopyridine (DMAP), N-[3-(methylamino)propyl]-N′-ethylcarbodiimide hydrochloride (EDC·HCl) were obtained from Sigma-Aldrich and Acros organics. Dichloromethane was freshly distilled before use. Reactions were monitored by TLC on Sorbfil plates. Column chromatography was carried out on Acrus silica gel (0.060–0.200 mm). Optical rotations were measured on a Perkin–Elmer 341 polarimeter (Perkin–Elmer, Waltham, MA, USA). IR spectra were recorded on Bruker VERTEX 70V (Bruker, Leipzig, Germany) using KBr discs over the range of 400–4000 cm^−1^. ^1^H and ^13^C NMR spectra were obtained using a Bruker AM-300 spectrometer (Bruker, Leipzig, Germany) in CDCl_3_ operating at 300.13 MHz for ^1^H and 75.47 MHz for ^13^C and a Bruker AV 600 spectrometer (Bruker, Leipzig, Germany) in CDCl_3_ operating at 600.1 MHz for ^1^H and 150.9 MHz for ^13^C. Mass spectra of MALDI TOF/TOF positive ions (matrix of sinapic acid) are recorded on a mass spectrometer Bruker AutoflexTM III Smartbeam (Bruker, Leipzig, Germany).

### 2.2. Cell Culturing

The human cell lines Jurkat, K562, U937, and HEK293 were obtained from the European Collection of Authenticated Cell Cultures (ECACC) and subsequently cultured in accordance with established standard protocols and sterile techniques. The cells were maintained in RPMI 1640 (for Jurkat, K562, and U937) or DMEM (for HEK293) medium, both of which were supplemented with 4 μM glutamine, 10% FBS (Sigma, Kanagawa, Japan), and 100 units/mL penicillin-streptomycin (Sigma). Subsequently, the cells were seeded into 24-well plates at a density of 5 × 10^4^ cells per well and incubated for 12 h (Jurkat, K562, U937) and 24 h (HEK293).

#### 2.2.1. Cytotoxicity Assay

The viability of the cells was evaluated through the utilization of the 7-AAD (7-aminoactinomycin D) dye (eBioscience™, San Diego, CA, USA). Following a 24 h incubation period with the test substances, the cells were washed with phosphate-salt buffer (PBS) and centrifuged at 400× *g* for five minutes. The precipitate was resuspended in 200 μL of flow cytometry buffer (PBS without calcium and magnesium, 2.5% FBS) and incubated with a 1 mM 7-AAD dye solution for 15 min in the dark at room temperature. Subsequently, all cell samples were subjected to analysis on a NovoCyte Penteon flow cytometer (Agilent, Santa Clara, CA, USA).

#### 2.2.2. Cell Cycle Analysis

Cell cycle analysis was performed using the MAK344 cell cycle assay kit (Sigma-Aldrich, St. Louis, MO, USA). Immediately after incubation with the tested compounds for 24 h, cells were gently washed with ice-cold phosphate-buffered saline (PBS) and centrifuged at 450× *g* for 5 min. After removing the supernatant, the cell precipitate was resuspended and fixed with 70% ethanol with continuous shaking on ice, and then stored at −18 degrees Celsius for 24 h. Prior to staining, the cells were washed with ice-cold PBS, and then 0.5 mL of the Cell Cycle Assay Kit working solution was added to the cell suspension at room temperature in the dark according to the working protocol. Samples were then analyzed on a NovoCyte Penteon flow cytometer (Agilent, USA).

#### 2.2.3. Multiparametric Analysis of Genotoxicity and Early Apoptosis

In this study, we employed Luminex technology (MILLIPLEX^®^ MAP 7-plex DNA Damage/Genotoxicity Magnetic Bead Kit and MILLIPLEX^®^ MAP Early Apoptosis 7-plex Magnetic Bead Kit) to analyze protein analytes in Jurkat cell lysates. The MILLIPLEX^®^ MAP 7-plex DNA Damage/Genotoxicity Magnetic Bead Kit (Cat. No. 48-621MAG) was employed for the simultaneous quantification of the following seven analytes in cell lysates: The following analytes were quantified: ATR (total), CHK1 (S345), CHK2 (T68), H2AX (S139), MDM2 (total), p21 (total), and p53 (S15). The MILLIPLEX^®^ 7-plex Early Apoptosis Magnetic Bead Kit (Cat. No. 48-669MAG) was employed for the simultaneous quantification of the following seven analytes: The following analytes were also quantified: AKT/PKB (S473), BAD (S112), BCL-2 (S70), activated caspase-8, activated caspase-9, JNK/SAPK1 (T183/Y185), and p53 (S46). The Luminex xMAP intracellular protein assay is a multiplex multiparameter assay that employs a multiplex of fluorescent-labeled antibodies and magnetic particles to enhance the sensitivity, dynamic range, and accuracy of the analytical method. Luminex technology is employed to identify the proteins of interest present in each well. The mean fluorescence intensity (MFI) of each measured protein is then converted to its concentration (pg·mL^−1^) using specialized software and standard curves.

The test substances were incubated with the cells at two concentrations, 0.5 and 1.0, for a period of six hours. The CC_50_ values for these concentrations are presented in Tables S1 and S2 of the Supporting Information, which also includes the exact values. Following exposure, approximately 1 × 10⁶ cells from each well were transferred to 0.5-mL tubes (Eppendorf, Hamburg, Germany) and washed with PBS. Subsequently, the PBS was removed, 10 μL of lysis buffer (Cell Signaling Buffer and Detection Kit, Luminex, Austin, TX, USA) supplemented with 2 × protease and phosphatase inhibitors (Luminex, USA) was added, and the mixture was shaken for 30 s. A cocktail of fluorescence-encoded magnetic bead microspheres (MagPlex^®^, Luminex, USA) was resuspended and treated with ultrasound. Subsequently, 70 μL of this cocktail, 70 μL of protein standard, and 70 μL of the test sample were added to the wells of a 96-well microplate and incubated for 2 h at room temperature with stirring on an orbital shaker at 700–850 rpm. A magnetic plate was firmly attached to the bottom of a 96-well plate, and all wells were washed three times with 100 μL of wash buffer (Cell Signaling Buffer and Detection Kit, Luminex, USA) added to each well to remove unbound particles. Subsequently, 50 μL of the was added to each well, and the samples were incubated for one hour at 4 °C on an orbital shaker set at 700–850 rpm. Subsequently, the samples were washed once more to remove any unbound antibodies. Afterwards, 70 μL of streptavidin-phycoerythrin (streptavidin-PE) (Luminex, USA) was added to each well and incubated for 30 min at room temperature on a shaker at 600 rpm. Subsequently, the samples were washed with streptavidin-PE and resuspended in 100 μL of wash buffer for a period of 2 min on a shaker at 800 rpm. The data were analyzed using the Milliplex Analyst 5.1 software (EMD Millipore, Billerica, MA, USA).

#### 2.2.4. Statistics

Comparisons between groups were conducted using one-way ANOVA for groups with one independent variable and two-factor ANOVA for groups with two independent variables. This was followed by the least significant difference (LSD) test. In the event that the mean of a given sample exhibited a deviation of greater than three standard deviations from the overall mean, the sample was excluded from the data set. A separate test of variance analysis was employed to evaluate the data pertaining to signaling pathway proteins. A statistical significance level of *p* < 0.05 was employed. A separate *t*-test with the Bonferroni-Dunn correction was employed to identify statistically significant effects.

## 3. Results

### 3.1. Chemistry

**Scheme 2:**
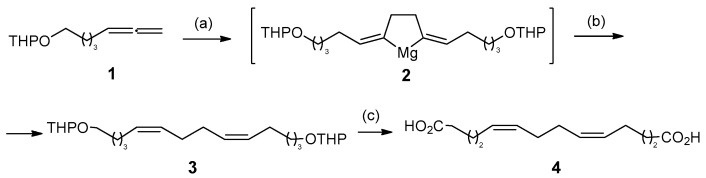
Synthesis of (5Z,9Z)-tetradeca-5,9-dienedioic acid **4** (a): EtMgBr, Mg, Cp_2_TiCl_2_ (5 mol%), diethyl ether; (b): H_3_O^+^; (c): H_2_CrO_4_/H_2_SO_4_, acetone, CH_2_Cl_2_.

Tetradeca-5Z,9Z-diene-1,14-dicarboxylic acid was synthesized in two steps using the previously developed [21]. *homo*-cyclomagnesiation of tetrahydropyran 5,6-heptadien-1-ol ether 1 with EtMgBr in the presence of the Cp_2_TiCl_2_ catalyst (5 mol%) to give magnesacyclopentane 2. The subsequent hydrolysis of organomagnesium compound 2 and oxidation of the resulting 1,14-bis-tetrahydropyranyl-5Z,9Z-diene-1,14-diol 3 with the Jones reagent gives target dicarboxylic acid 4 in an overall yield of ~52% (Scheme 2).

**Scheme 3:**
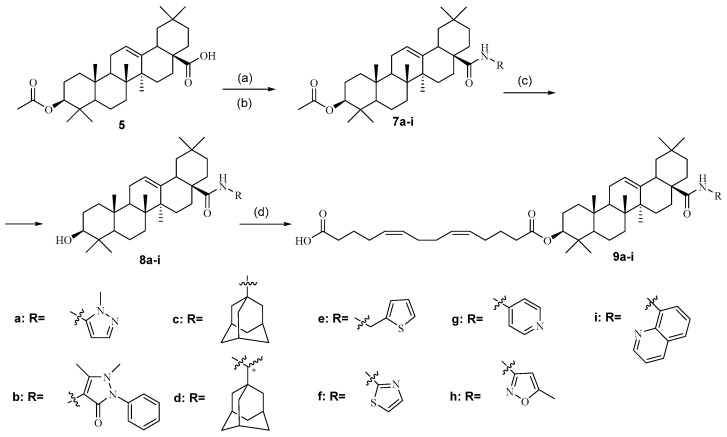
Synthesis of conjugates of oleanolic and 5Z,9Z-dienoic acid containing a heterocyclic or carbocyclic moiety **9a**–**i**. Reagents and conditions: (a): oxalyl chloride, anhydrous CH_2_Cl_2_, rt, 16 h; (b): heterocyclic or carbocyclic amine **6**, DIPEA, anhydrous CH_2_Cl_2_, rt (yield 75‒85%); (c): NaOH (4M), THF, MeOH, rt (yield ~95%); (d): (5Z,9Z)-tetradeca-5,9-dienedioic acid **4**, EDC·HCl, DMAP, CH_2_Cl_2_ (60‒65%)

The synthesis of hybrid molecules based on oleanolic and tetradeca-5Z,9Z-dien-1,14-dicarboxylic acids was conducted in a series of steps. Initially, hetero- and carbocyclic derivatives of oleanolic acid (**7a**–**i**) were obtained through the interaction of 3β-O-acetyloleanolic acid (5) with heterocyclic and carbocyclic amines (6) in two stages, in accordance with the methodology outlined in reference [22]. Following the removal of the acyl protecting groups by hydrolysis in an alkaline medium, oleanolic acid amides with hetero- and carbocyclic substituents (**8a**–**i**) were obtained, which subsequently participated in a reaction with tetradeca-5Z,9Z-dien-1. The 14-dicarboxylic acid 4 was reacted with N-(3-dimethylaminopropyl)-N′-ethylcarbodiimide hydrochloride (EDC-HCl) and catalytic amounts of 4-dimethylaminopyridine (DMAP) in dichloromethane to yield the target hybrid molecules **9a**–**i** (Scheme 3, Figures S1–S54 in Supplementary Materials).

### 3.2. Biological Evaluation

#### 3.2.1. Cytotoxic Activity In Vitro

**Figure 1:**
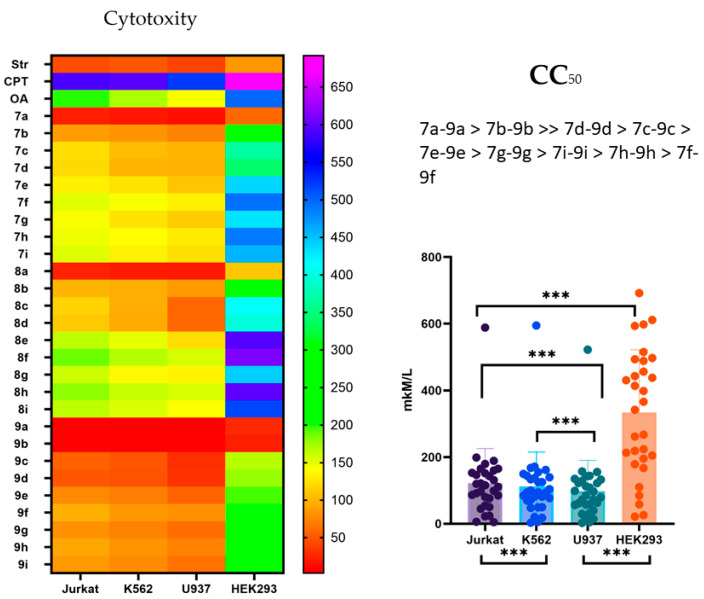
The in vitro cytotoxic activity (CC50) of the oleanolic acid-based hybrid molecules synthesized in this study was evaluated on cell cultures of Jurkat, K562, A549, and HEK293 cell lines. The presence of three asterisks (*p* < 0.01) indicates that the observed differences between CC50 values for the four cell lines are statistically significant. The table in the Supplementary Information File lists the exact CC50 values for each compound in each of the four cell lines. A statistical analysis was conducted using regression analysis and stepwise analysis of variance (one-step ANOVA). The incubation period was 24 h.

The cytotoxicity of the synthesized compounds was evaluated on one adherent and three suspension cell lines. The cell lines were designated as HEK293, Jurkat, K562, and U937, respectively. The data pertaining to the cytotoxicity of the aforementioned compounds are presented in Figure 1 and Table S1 of the Supplementary Information File. A comparison of the cytotoxicity parameters of each compound against a particular cell line revealed that Jurkat cells exhibited the highest sensitivity to this compound library, while HEK293 cells demonstrated the highest resistance. The cytotoxic effect of the synthesized compounds and the original oleanolic acid on the selected cell lines, with a few exceptions, increased in a predictable manner. The order of resistance was U937 **>** K562 **>** Jurkat **>>** HEK293. All synthesized compounds have been observed to exhibit higher levels of cytotoxicity than the original oleanolic acid. In each group of compounds, irrespective of the structure and nature of the attached amine, an increase in cytotoxicity is observed in the series **7 > 8 >> 9**. Among the synthesized compounds, compounds **9a** and **9b**, which contain a N-methyl-pyrazolic and a 1,5-dimethyl-3-oxo-2-phenyl-2,3-dihydro-1H-pyrazolic fragment, respectively, exhibit the highest cytotoxicity. In general, the SS50 value decreases in each series of synthesized compounds. The cytotoxicity of the compounds decreases in the following order: **7a**–**9a > 7b**–**9b > 7d**–**9d > 7c**–**9c > 7e**–**9e > 7g**–**9g > 7i**–**9i > 7h**–**9h > 7f**–**9f.** The selectivity index (SI) ranges from three to seven for the Jurkat/HEK293 ratio, from three to nine for the K562/HEK293 ratio, and from two to thirteen for the U937/HEK293 ratio.

#### 3.2.2. Genotoxicity and Apoptosis Induction Studies

The development of efficacious therapies for cancer and other diseases, characterized by abnormalities in the regulation of cell death, hinges on a comprehensive understanding of the diverse mechanisms through which cells can lose viability and ultimately undergo apoptosis. It is not uncommon for multiple mechanisms of cell death to be present in a single cell when it is subjected to the effects of a toxic agent under investigation. The process of apoptosis is highly conserved across multicellular organisms and is subject to strict genetic control [23]. Apoptosis may be initiated by the cell itself when it detects damage through a number of intracellular sensors, a process known as the intrinsic pathway. Conversely, apoptosis may result from interaction with a toxic agent via death receptors, which then activate a caspase-free cascade, known as the extrinsic pathway of apoptosis [24].

**Figure 2:**
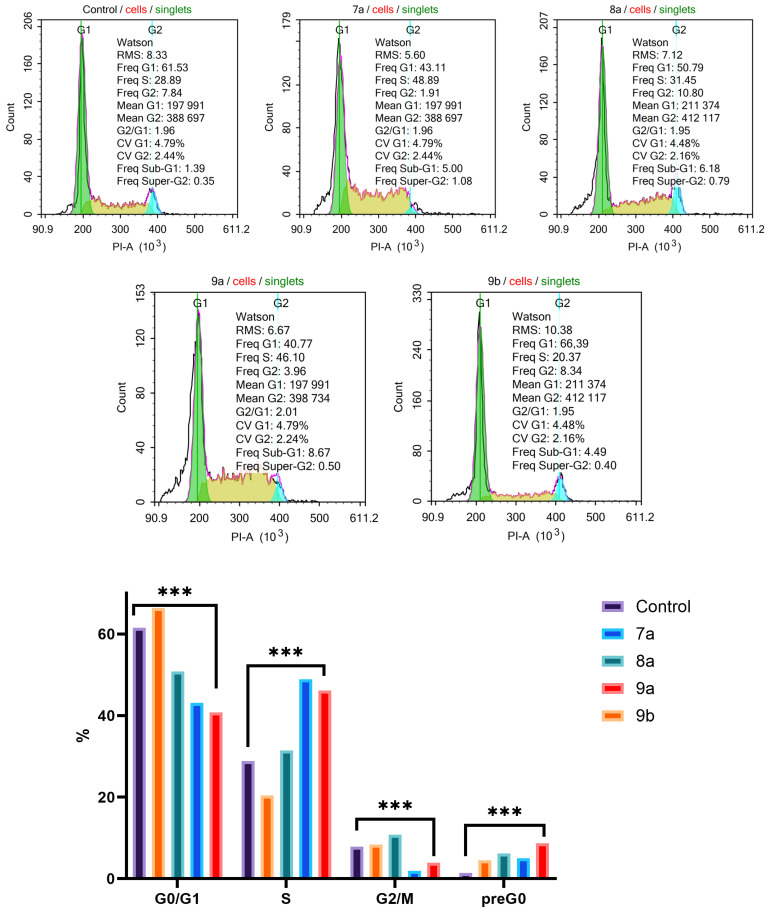
Illustrates the ratio of apoptosis phases in Jurkat cells following exposure to lithocholic acid-based ionic compounds. All synthesized compounds were administered at a concentration corresponding to the CC50 value, and the period of incubation was 24 h. The presence of three asterisks (*p* < 0.01) indicates that the observed differences between the experimental and control values are statistically significant. This was determined through one-way ANOVA comparisons.

The hybrid molecules with the highest CC_50_ value, namely **7a**, **8a**, **9a**, and **9b**, were selected based on their cytotoxicity (Figure 1, Table S1 in the Supplementary Materials) as the most promising candidates for further investigation. The synthesized compounds were incubated with Jurkat cells to evaluate the effect on the cell cycle, followed by an analysis of the signaling pathways involved in genotoxic stress and programmed cell death. The effects of selected compounds on the cell cycle of Jurkat cells are illustrated in Figure 2.

A comparative analysis of the effect of selected synthesized compounds at CC_50_ concentration on the cell cycle demonstrated that the synthesized hybrid molecules exhibited a pronounced suppressive effect on all phases of the cell cycle. For instance, when the G1 phase is considered, ionic compound **9a** is the most effective, resulting in a reduction in the cell population in the G1 phase to 40.70% compared to the control (61.53%, respectively). Additionally, compound **9a** demonstrates the highest percentage of cells in the pre-G0 phase (8.67%), which provides evidence of apoptosis induction (Figure 2). Upon analysis of the S-phase, it was observed that two compounds, **7a** and **9a**, exhibited the greatest increase in cell population within this phase (48.89% and 46.10%, respectively) in comparison to the control. Furthermore, compounds **7a** and **9a** were observed to reduce the percentage of cells in the G2/M phase (1.91 % and 3.96%, respectively). Therefore, hybrid molecule **9a**, which is based on oleanolic acid and the 1Z,5Z-diene fragment, affects all phases of the cell cycle, but it has a particularly pronounced effect on G1, pre-G0, and G2 phases (Figure 2).

#### 3.2.3. A Study of the Major Kinases of Signalling Pathways Involved in Genotoxicity and Apoptosis

The Jurkat cell line was selected for investigation into the intracellular signaling of genotoxicity and the induction of apoptosis. Recent studies have demonstrated that nonsense mutations in exon 6 of the TP53 gene, which are characteristic of the Jurkat cell line, result in the production of a shortened p53 isoform that appears to evade nonsense-mediated decay. These isoforms, designated p53 ψ, exhibit a deficiency in the canonical p53 transcriptional activity. Alternatively, they localize to mitochondria, where they activate a pro-tumorigenic cellular program by regulating mitochondrial transition pore permeability through interaction with cyclophilin D [25]. The expression of the p53 ψ isoform by the Jurkat cell line may contribute to the previously reported increased calcium release upon TCR activation [26]. Furthermore, the researchers published data indicating that Jurkat cells express p53 ψ, a recently discovered p53 isoform with a direct link to malignant transformation. The presented data indicate that this cell line may serve as a model system for investigating the non-canonical activity of p53 ψ [27]. Our previous research demonstrated that a shortened p53 ψ transcript is expressed in Jurkat cells in response to exposure to ionic compounds, with the level of an expressed protein exhibiting considerable variability [28].

The cellular response to DNA damage is primarily coordinated by two signaling pathways, namely ATM-Chk2 and ATR-Chk1. These pathways are activated by double-stranded and single-stranded DNA breaks, respectively. It has been demonstrated that these pathways operate in parallel and can overlap [29]. However, over the past decade, it has become increasingly evident that their relationship is more intricate and nuanced than previously assumed. In response to double-stranded DNA damage, ATM is required for both the activation of ATR-Chk1 and the initiation of DNA repair by homologous recombination (HRR). This promotes the formation of single-stranded DNA at sites of damage by nucleolytic resection. It has been demonstrated that cells and organisms can survive with mutations in ATM or in the genes of other proteins that are required for homologous recombination repair (HRR), such as BRCA1 and BRCA2. However, this survival is associated with an increased risk of genomic instability and cancer predisposition. In contrast, the ATR-Chk1 pathway plays a pivotal role in the direct response to DNA damage and replication checkpoints, thus being indispensable for the survival of numerous cell types, although not all [29]. The MDM2 protein has been demonstrated to exert a negative regulatory effect on the p53 tumor suppressor protein. The tumor suppressor protein p53 responds to cellular stress by activating the transcription of several genes that are responsible for DNA repair, cell cycle arrest, anti-angiogenesis, apoptosis, and autophagy. The specific downstream pathway activated by p53 is contingent upon a number of factors, including the severity of the stress experienced, the nature of the stressor, and the cell type in question. The regulation of p53 primarily occurs at the level of protein stability within the regulatory network. This involves p53 being polyubiquitinated by MDM2 and subsequently cleaved by the 26S proteasome [30]. A pivotal element of this network is the p53/MDM2 feedback loop, wherein p53 turnover is regulated by MDM2, and MDM2 expression is subject to the transcriptional control of p53 [31]. It can be reasonably deduced that the MDM2-p53 protein complex represents a convenient universal target for p53 reactivation during the process of cancer transformation in tissues [32]. A number of chemotherapeutic agents, including etoposide, camptothecin, and their derivatives, have been observed to damage tumor cells by inducing double-strand DNA breaks. The H2A.X protein is a member of the histone H2A family. The level of γ-H2A.X detected by flow cytometry has been demonstrated to correlate with the number of DNA strand breaks and tumor cell death. The process of serine phosphorylation at position 139 of the H2A.X protein serves as a reliable indicator of DNA damage, thereby enabling the assessment of the genotoxicity of the compound with sufficient confidence [33,34]. As the level of DNA damage increases, the level of phosphorylated H2A.X also rises, accumulating specifically at sites of damage to the DNA molecule. The accumulation of phosphorylated H2A.X is frequently employed as a marker of the level of DNA damage within the cell and plays a pivotal role in DNA repair processes [35,36].

**Figure 3:**
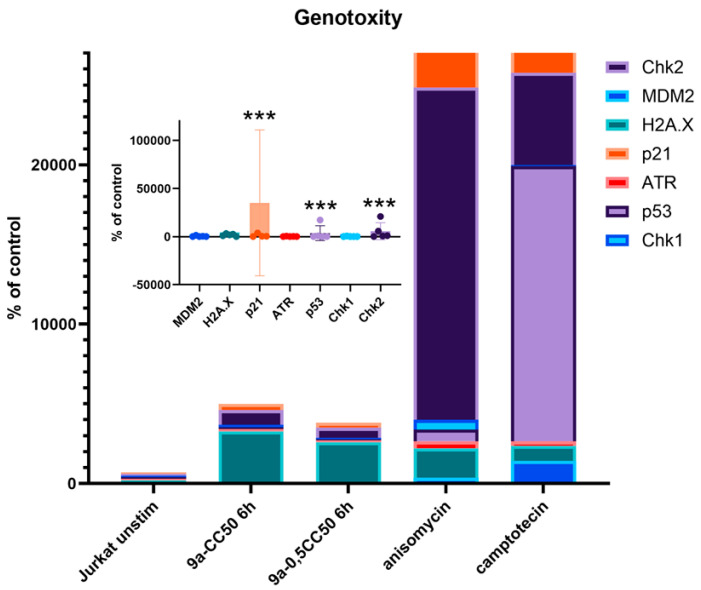
Genotoxic effect of compound **9a** on Jurkat cells after a 6 h exposure compared to the control sample (untreated cells). Anisomycin (IC_50_ 0.192 μmol/L) and camptothecin (CC50 concentration of the compound is presented in Table S1 in the Supplementary Materials) are shown for comparison. Exact values are given in Table S2 in the Supporting Information section. The presence of three asterisks (*p* < 0.01) indicates that the results are significantly different from the control. The experiment was conducted in triplicate. One-way ANOVA, *p* < 0.05, *p* < 0.01, and *p* < 0.001. “ns” indicates no significant difference.

The effects of compound **9a** were examined at two concentrations (CC_50_ and 0.5CC_50_). Compounds and two control compounds, anisomycin, and camptothecin, were incubated with Jurkat cells for six hours. Subsequently, seven proteins involved in different signaling pathways that are activated by the genotoxic effects of chemical compounds were determined in the lysate of experimental and control samples using a kit (MILLIPLEX^®^ MAP 7-plex DNA Damage/Genotoxicity Magnetic Bead Kit and MILLIPLEX^®^ MAP Early Apoptosis 7-plex Magnetic Bead Kit panels) (Figure 3, Table S2). Therefore, when Jurkat cells were exposed to compound **9a** for six hours, a dose-dependent increase in the primary marker of DNA double-strand breaks, phosphorylated H2A.X, was observed. In comparison to the control compound camptothecin, the level of phosphorylated H2A.X was found to be approximately fourfold higher at CC_50_ and 3.5-fold higher at 0.5CC_50_. Additionally, our study examined anisomycin (Flagecidin), a bacterial antibiotic derived from Streptomyces griseolus, which inhibits protein synthesis and also functions as a JNK activator. Anisomycin has been demonstrated to stimulate autophagy and increase apoptosis [37,38]. Anisomycin, at a concentration of IC_50_ (0.192 μmol/L), when exposed to Jurkat cells, exhibits a slightly different pattern. In addition to an increase in H2A.X protein, the concentration of Chk1, Chk2, MDM2, and ATR proteins increases, indicating the formation of single- and double-stranded DNA breaks. Camptothecin exhibits one of the highest levels of MDM2 kinase activity among the compounds under examination. It is established that pronounced DNA degradation exerts an inhibitory effect on MDM2 (apoptosis commences within the initial hours, as the cell is unable to repair the damaged DNA). Conversely, moderate DNA degradation results in an increase in MDM2, which forms a p53 inhibitory complex. This allows the cell to initiate a repair program, even if unsuccessful, ultimately leading to apoptosis.

Cyclin-dependent kinase 1A inhibitor (CDKN1A/p21) is a well-studied protein with a recognized role in the cell cycle. It induces cell cycle arrest by inhibiting the activity of cyclin-dependent kinases (CDKs), making it a key regulator of this process. Extensive studies conducted over the years have illuminated various additional mechanisms associated with CDKN1A/p21, implicating it in processes such as apoptosis, DNA damage response (DDR), and regulation of stem cell differentiation. Furthermore, the p21 protein functions as an anti-apoptotic protein by inhibiting the cell cycle. The observed increase in p21 levels concomitant with an increase in p53, H2A.X, and Chk1 protein levels suggests that cells are attempting to repair DNA damage caused by anisomycin and camptothecin. In samples treated with compound **9a**, the level of p21 protein is nearly indistinguishable from the control, suggesting that DNA repair is irreversible and unfeasible in cells when exposed to the synthesized hybrid molecule. Furthermore, the samples of cells treated with compound **9a** exhibited an increase in phosphorylated H2A.X, which corroborates the occurrence of DNA degradation in tumor cells (Figure 3).

The process of apoptosis is a complex biological phenomenon that is associated with the activation of numerous signaling pathways. In each case, specific proteins are activated, which can act as “markers” of certain intracellular processes. The imbalance of these processes can lead to the activation of programmed cell death. The activation of cysteine proteases, in particular caspases, represents a pivotal intracellular regulator of cell apoptosis. Caspases are involved in both the intrinsic and extrinsic pathways of apoptosis [39]. Caspase-3 plays a pivotal role in apoptosis [40], a process that is initiated by various activators and can be broadly classified into two major signaling pathways: the death receptor-mediated pathway, which involves caspase-8 and caspase-10, and the mitochondrial-mediated pathway, which involves caspase-9 [41]. Caspase-3 is the principal protease activated by both FAS receptor ligands and cellular apoptosis induced by mitochondrial dysfunction [42]. The tumor suppressor protein p53 induces the expression of several pro-apoptotic proteins, including Bax, Bad, and Bak, which prevent the capture of Bcl-2. Subsequently, free Bax, Bad, and Bak bind to the mitochondrial membrane, resulting in mitochondrial damage and cellular apoptosis [43]. Prior research has demonstrated that p53 facilitates the transcription of Bax and Bad, which regulate the release of cytochrome c from mitochondria and induce cellular apoptosis by activating the cleavage of caspase-3 and caspase-9 [44]. It is currently understood that Bcl-2 and Bax proteins exist in a state of constant dynamic equilibrium, forming homo- and heterodimers. When the production of the proapoptotic protein Bax is dominant, this equilibrium is disrupted, resulting in a shift towards the formation of a substantial number of homodimers with elevated pro-apoptogenic activity [45]. Jun N-terminal kinases (JNKs) play a pivotal role in both extrinsic and intrinsic apoptotic pathways initiated by death receptors. JNKs activate apoptotic signaling pathways by upregulating pro-apoptotic genes. This is achieved through two main mechanisms: transactivation of specific transcription factors and direct modulation of mitochondrial pro- and anti-apoptotic protein activity through various phosphorylation events [46].

**Figure 4:**
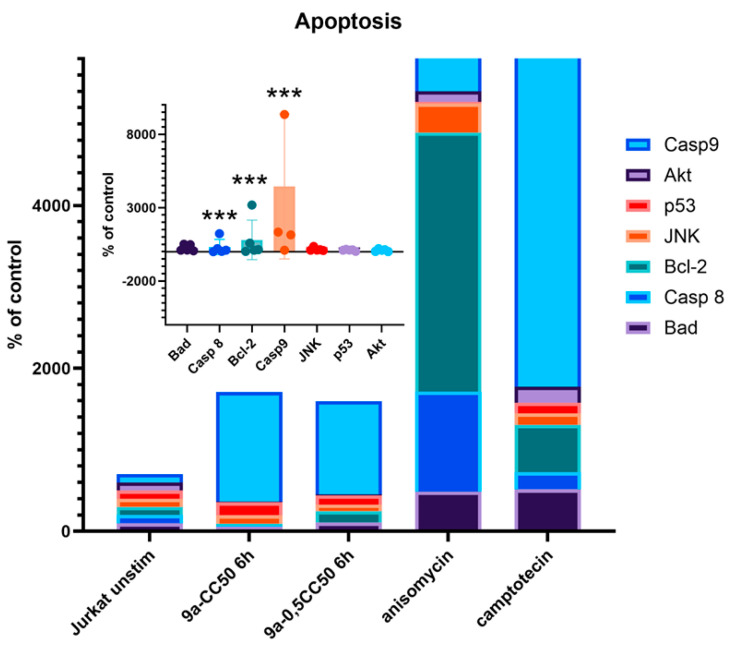
The induction of apoptosis by compound **9a** in Jurkat cells after a 6 h exposure compared to the control sample (untreated cells). Anisomycin (IC50 0.192 μmol/L) and camptothecin (CC50 concentration of the compound is presented in Table S1) are shown for comparison. Exact values are given in Table S2 in the Supporting Information section. The presence of three asterisks (*p* < 0.01) indicates that the results are significantly different from the control. The experiment was conducted in triplicate. One-way ANOVA, *p* < 0.05, *p* < 0.01, and *p* < 0.001. “ns” indicates no significant difference.

The effects of compound **9a** were examined at two concentrations (CC_50_ and 0.5CC_50_) and two control compounds, anisomycin, and camptothecin, were incubated with Jurkat cells for six hours. Following this incubation period, seven proteins of different signaling pathways responsible for the induction of different apoptosis pathways in the cell were identified in the lysate of experimental and control samples using a kit (MILLIPLEX^®^ MAP 7-plex DNA Damage/Genotoxicity Magnetic Bead Kit and MILLIPLEX^®^ MAP Early Apoptosis 7-plex Magnetic Bead Kit panels) (Figure 4, Table S3). It is noteworthy that the role of JNK is still a topic of considerable debate among researchers. While the pro-apoptotic role of JNK is well established, it is important to recognize that the anti-apoptotic role of JNK is often influenced by a number of factors. Such factors may include the parallel activation of cell survival or anti-apoptotic pathways and the overall strength of apoptotic signaling. It appears that sustained JNK activation is associated with apoptosis, whereas acute and transient JNK activation is involved in the proliferative or cell survival pathway [47].

Thus, when analyzing cell samples treated with compound **9a** at concentrations of CC_50_ and 0.5CC_50_, a pronounced increase in caspases 9, 8, and JNK in the first 6 h of incubation is observed, which is a reliable indicator that the new hybrid molecule induces apoptosis in Jurkat cells via a p53-independent pathway. This is of particular significance for cells that have mutated p53, which are the cells of most malignant tumors. In samples treated with anisomycin and camptothecin, the significant increase in Bcl-1 protein levels relative to the control group is noteworthy. The majority of members of the BCL2 family, which are associated with apoptosis, regulate cell fate in response to antitumor agents. Modulations in the mRNA and protein levels of these genes are commonly associated with the sensitivity or resistance of different cancer cell types to chemotherapeutic agents. Furthermore, alterations in the expression of BCL2 family members induced by antitumor treatment may facilitate apoptosis in tissues with resistance to the majority of chemotherapeutic agents [47,48,49].

## 4. Conclusions

5Z,9Z-dienoic acids demonstrate considerable potential for biological activity against the primary enzymes that relieve torsional stress from the DNA molecule during its biosynthesis. They have been observed to inhibit a range of topoisomerases in humans, animals, and bacteria. This provides a powerful and innumerable pool of combinations of these pharmacophores carrying 5Z,9Z-diene moieties, which can be used to create a wide range of substances with specified properties. Furthermore, the 5Z,9Z-diene system in the molecule is distinctive in that it inhibits topoisomerase through a mechanism distinct from that of camptothecin, the primary and currently known “topoisomerase poison”. It causes what is referred to as reversible inhibition, which allows the topoisomerase enzyme to contribute less to the occurrence of non-homologous DNA recombination in the cell due to irreversible inhibition. At the same time, it provides the necessary inhibition of the enzyme during active DNA synthesis in tumors. The aforementioned characteristics permit the creation of biologically active molecules containing a 5Z,9Z-diene pharmacophore, exhibiting slightly reduced cytotoxicity and a diminished toxic impact on healthy tissues in comparison to camptothecin. In the present work, a series of hybrid molecules based on oleanolic acid and 5Z,1Z-diene fragments was synthesized. Hybrid molecule **9a** has been shown to affect the cell cycle by increasing the population in the pre-G0 phase, and also possesses apoptosis-inducing ability through activation of JNK kinase and inducing the accumulation of caspases 8 and 9 in cells. The synthesized compound **9a** is also shown to exhibit genotoxic effects through the accumulation of phosphorylated H2AX protein and activation of Chk1. All of the above effects of the hybrid compound we prepared indicate its high anticancer potential, and it is very likely that this molecule will activate apoptosis in p53-deficient tumor tissues.

## Data Availability

The data presented in this study are available in this article.

## Supplementary Materials

The following supporting information can be downloaded at: https://www.mdpi.com/article/10.3390/cancers16233893/s1, Figures S1–S54: 1H and 13C NMR Spectra of the synthesized compounds; Table S1: Numerical CC50 values for each compound in Jurkat, K562, U937, and HEK293 cell lines. Table S2: Changes in genotoxicity-related protein levels (% of control) in Jurkat cells upon exposure to compound **9a**. Table S3: Changes in apoptosis-related protein levels (% of control) in Jurkat cells upon exposure to compound **9a**.
