# Supplementary material for: New Synthetic Analogs of Natural 5Z,9Z-Dienoic Acids—Hybrid Molecules Based on Oleanolic Acid: Synthesis and Study of Antitumor Activity
Source: Cancers (Basel). 2024 Nov 21;16(23):3893. doi: 10.3390/cancers16233893 (PMC11640419; doi:10.3390/cancers16233893)
Supplement: Supplementary file 1 [file cancers-16-03893-s001.zip › cancers-3310094-supplementary.pdf]

Supporting Information for

New Synthetic Analogs of Natural 5Z,9Z-Dienoic Acids - Hybrid Molecules Based on Oleanolic Acid: Synthesis and Study of Antitumor Activity

Regina A. Tuktarova [1,2], Lilya U. Dzhemileva [1,*], Usein M. Dzhemilev [1], and Vladimir A. D'yakonov [1,*]

[1] N.D. Zelinsky Institute of Organic Chemistry, Russian Academy of Sciences, Leninsky Prospect 47,Moscow, 119991, Russia
[2] ITMO University, Kronverksky Prospekt 49, Saint Petersburg, 191002, Russia

*Correspondence: DyakonovVA@gmail.com (V.A.D.); Dzhemilev@mail.ru (L.U.D.);

Table of contents

1. Experimental data for the compounds **7a-7i, 8a-8i, 9a-9i**

2. ^{1}H and ^{13}C NMR Spectra of the compounds **7a-7i, 8a-8i, 9a-9i** (Figures **S1**-**S54**)

3. **Table S1.** Numerical CC50 values for each compound in Jurkat, K562, U937, and HEK293 cell lines

4. **Table S2**: Changes in genotoxicity-related protein levels (% of control) in Jurkat cells upon exposure to compound **9a**

5. **Table S3**: Changes in apoptosis-related protein levels (% of control) in Jurkat cells upon exposure to compound **9a**

Chemical Experimental data for the compounds 7a-7i, 8a-8i, 9a-9i

^{1}H NMR ($CDCl_3$, 500 MHz) and ^{13}C NMR ($CDCl_3$, 125 MHz) spectral data and synthesis method for (5*Z*,9*Z*)-tetradeca-5,9-dienedioic acid **5** are described in the literature [V.A. D'yakonov, L.U. Dzhemileva, R.A. Tuktarova, A.A. Makarov, I.I. Islamov, A. R. Mulyukova, U.M. Dzhemilev, Catalytic cyclometallation in steroid chemistry III1: Synthesis of steroidal derivatives of 5Z,9Z-dienoic acid and investigation of its human topoisomerase I inhibitory activity, Steroids 102 (2015) 110–117].

*General procedures for synthesis of oleanolic acid with hetero- and carbocyclic moiety 7**a-i**.*

To a solution of (3*β*)-3-(acetyloxy)olean-12-en-28-oic acid **5** (0.49 g, 1.0 mmol) in anhydrous CH_2Cl_2 (30 mL) at 0 °C, oxalyl chloride (1.5 mL, 18.0 mmol) was added. After stirring at room temperature overnight, the mixture was evaporated, and co-evaporated with CH_2Cl_2 (3 × 10 mL). The residue was dissolved in anhydrous CH_2Cl_2 (30 mL), and then DIPEA (0.4 mL, 3.0 mmol) and heterocyclic or carbocyclic amine (1.2 mmol) were added at 0 °C. After stirring at rt for 24 h, the solvent was evaporated, and the residue was purified by chromatography on silica gel using hexane/ethyl acetate (5:1 to 1:2) as mobile phase, affording the product **7a-i**.

(3*β*)-28-[(1-Methyl-1*H*-pyrazol-5-yl)amino]-28-oxoolean-12-en-3-yl acetate (**7a**)

Yield: 0.48 g, 83%, white crystals, mp 268–270 °C. $[\alpha]_D^{22}$ + 37.0 (*c* 0.83, $CHCl_3$); IR (KBr) ν_{max} 2947, 2930, 2876, 1733, 1717, 1684, 1558, 1457, 1371, 1149, 1090, 1009, 926, 756, 651 cm^{-1}; ^{1}H NMR ($CDCl_3$, 500 MHz) *δ* 7.76 (1H, br s, NH), 7.33 (1H, m, pyrazolyl), 6.14 (1H, m, pyrazolyl), 5.45 (1H, m, H-12), 4.43 (1H, t, *J* = 8.5 Hz, H-3), 3.63 (3H, s, NCH_3), 2.71–0.78 (23H, m), 2.00 (3H, s, CH_3CO), 1.17 (3H, s, H-27), 0.91 (6H, s, H-25, H-30), 0.89 (3H, s, H-29), 0.82 (6H, s, H-23, H-24), 0.75 (3H, s, H-26); ^{13}C NMR ($CDCl_3$, 125 MHz) *δ* 176.7 (C-28), 170.9 ($CH_3\underline{C}O$), 144.6 (C-13), 138.2 (pyrazolyl), 135.8 (pyrazolyl), 123.4 (C-12), 99.4 (pyrazolyl), 80.7 (C-3), 55.1 (C-5), 47.4 (C-9), 47.2 (C-17), 46.4 (C-19), 42.3 (C-14), 42.1 (C-18), 39.3 (C-8), 38.1 (C-1), 37.6 (C-4), 36.8 (C-10), 35.5 (NCH_3), 34.0 (C-21), 32.9 (C-29), 32.7 (C-22), 32.3 (C-7), 30.7 (C-20), 27.9 (C-23), 27.3 (C-15), 25.7 (C-27), 23.8 (C-16), 23.6 (C-30), 23.5 (C-2, C-11), 21.3 ($\underline{C}H_3CO$), 18.1 (C-6), 17.2 (C-26), 16.7 (C-24), 15.4 (C-25); anal. calcd for $C_{36}H_{55}N_3O_3$: C, 74.83; H, 9.59; found C, 74.78; H, 9.54. MALDI TOF: m/z 578.445 ($[M]^+$, calcd 578.419).

28-[(1,5-Dimethyl-3-oxo-2-phenyl-2,3-dihydro-1*H*-pyrazol-4-yl)amino]-28-oxoolean-12-en-3-yl acetate (**7b**)

Yield: 0.54 g, 79%, white solid. $[\alpha]_D^{21}$ + 24.5 (*c* 0.82, $CHCl_3$); IR (KBr) ν_{max} 2944, 2875, 1733, 1713, 1668, 1596, 1497, 1456, 1394, 1365, 1246, 1047, 1008, 986, 759, 650 cm^{-1}; ^{1}H NMR ($CDCl_3$, 500 MHz) *δ* 7.47–7.26 (6H, m, Ph, NH), 5.45 (1H, m, H-12), 4.50 (1H, t, *J* = 8.5 Hz, H-3), 3.04 (3H, s, NCH_3), 2.85–0.82 (23H, m), 2.28 (3H, s, CCH_3), 2.05 (3H, s, CH_3CO), 1.19 (3H, s, H-27), 0.95 (3H, s, H-25), 0.94 (3H, s, H-30), 0.92 (3H, s, H-29), 0.87 (3H, s, H-23), 0.85 (3H, s, H-24), 0.84 (3H, s, H-26); ^{13}C NMR ($CDCl_3$, 125 MHz) *δ* 176.9 (C-28), 170.9 ($CH_3\underline{C}O$), 161.7 (C=O), 148.6 (CH=), 143.7 (C-13), 134.8 (Ph), 129.1 (Ph), 126.6 (Ph), 123.7 (Ph), 123.2 (C-12), 109.5 (CH=), 80.9 (C-3), 55.3 (C-5), 47.6

(C-9), 47.2 (C-17), 46.5 (C-19), 42.0 (C-14, C-18), 39.4 (C-8), 38.2 (C-1), 37.7 (C-4), 36.9 (C-10), 36.5 (NCH_3), 34.2 (C-21), 33.3 (C-22), 33.0 (C-29), 32.6 (C-7), 30.7 (C-20), 28.0 (C-23), 27.5 (C-15), 25.7 (C-27), 23.8 (C-16), 23.6 (C-30), 23.5 (C-2, C-11), 21.3 ($\underline{C}H_3CO$), 18.2 (C-6), 17.2 (C-26), 16.7 (C-24), 15.5 (C-25), 12.9 ($C\underline{C}H_3$); anal. calcd for $C_{43}H_{61}N_3O_4$: C, 75.51; H, 8.99; found C, 75.48; H, 8.84. MALDI TOF: m/z 683.345 ($[M]^+$, calcd 683.466).

(3*β*)-28-(1-Adamantylamino)-28-oxoolean-12-en-3-yl acetate (**7c**)

Yield: 0.53 g, 84%, white crystals, mp 280–282 °C. $[\alpha]_D^{19}$ + 39.5 (*c* 0.65, $CHCl_3$); IR (KBr) ν_{max} 2909, 2851, 1733, 1653, 1508, 1456, 1362, 1246, 1094, 1028, 754, 665 cm^{-1}; ^1H NMR ($CDCl_3$, 500 MHz) *δ* 5.53 (1H, br s, NH), 5.33 (1H, m, H-12), 4.49 (1H, t, *J* = 8.5 Hz, H-3), 2.53–0.83 (23H, m), 2.05 (6H, m, CH_3CO, adamantyl), 1.97, 1.67 (12H, m, adamantyl), 1.16 (3H, s, H-27), 0.97 (3H, s, H-25), 0.90 (9H, s, H-26, H-29, H-30), 0.88 (3H, s, H-23), 0.86 (3H, s, H-24); ^{13}C NMR ($CDCl_3$, 125 MHz) *δ* 176.9 (C-28), 170.9 ($CH_3\underline{C}O$), 144.8 (C-13), 122.3 (C-12), 80.8 (C-3), 55.2 (C-5), 51.4 (adamantyl), 47.5 (C-9), 46.9 (C-19), 46.5 (C-17), 42.4, 42.2 (C-14, C-18), 41.4 (adamantyl), 39.5 (C-8), 38.2 (C-1), 37.7 (C-4), 36.9 (C-10), 36.5 (adamantyl), 34.3 (C-21), 33.0 (C-22, C-29), 32.6 (C-7), 30.7 (C-20), 29.5 (adamantyl), 28.0 (C-23), 27.4 (C-15), 25.5 (C-27), 23.9 (C-16), 23.5 (C-2, C-11, C-30), 21.3 ($\underline{C}H_3CO$), 18.2 (C-6), 17.7 (C-26), 16.7 (C-24), 15.6 (C-25); anal. calcd for $C_{42}H_{65}NO_3$: C, 77.02; H, 10.00; found C, 76.89; H, 9.94. MALDI TOF: m/z 632.516 ($[M+H]^+$, calcd 632.504), 654.513 ($[M+Na]^+$, calcd 654.486).

(3*β*)-28-{[1-(1-Adamantyl)ethyl]amino}-28-oxoolean-12-en-3-yl acetate (**7d**)

Yield: 0.52 g, 80%, white solid. $[\alpha]_D^{25}$ + 23.3 (*c* 0.81, $CHCl_3$); IR (KBr) ν_{max} 2926, 2906, 2849, 1733, 1650, 1609, 1507, 1452, 1366, 1246, 1095, 1026, 754, 665, 608 cm^{-1}; ^1H NMR ($CDCl_3$, 500 MHz) *δ* 5.49 (5.48) (1H, br s, NH), 5.32 (1H, m, H-12), 4.49 (1H, t, *J* = 8.5 Hz, H-3), 3.64 (1H, m, CH), 2.83–0.82 (23H, m), 2.05 (3H, m, CH_3CO), 1.99–1.96 (3H, m, adamantyl), 1.72–1.68 (1.64–1.60) (6H, m, adamantyl), 1.56–1.53 (1.44–1.40) (6H, m, adamantyl), 1.15 (3H, s, H-27), 1.00 (0.99) (3H, d, *J* = 6.5 Hz, CH_3), 0.93 (6H, s, H-25, H-30), 0.91 (3H, s, H-29), 0.86 (3H, s, H-23), 0.85 (3H, s, H-24), 0.82 (3H, m, H-26); ^{13}C NMR ($CDCl_3$, 125 MHz) *δ* 176.4 (C-28), 170.9 ($CH_3\underline{C}O$), 143.8 (C-13), 122.3 (C-12), 80.9 (C-3), 55.3 (C-5), 52.8 (CH), 47.5 (C-9), 46.5 (C-19), 46.2 (C-17), 41.9 (C-14, C-18), 39.4 (C-8), 38.6 (adamantyl), 38.1 (C-1), 37.7 (C-4), 37.1 (adamantyl), 36.9 (C-10), 35.7 (adamantyl), 34.2 (C-21, C-22), 33.1 (C-29), 33.0 (C-7), 30.7 (C-20), 28.3 (adamantyl), 28.1 (C-23), 27.4 (C-15), 25.6 (C-27), 23.6 (C-16, C-30), 23.4 (C-2, C-11), 21.3 ($\underline{C}H_3CO$), 18.2 (C-6), 17.9 (C-26), 16.7 (C-24), 15.4 (C-25), 14.5 (CH_3); anal. calcd for $C_{44}H_{69}NO_3$: C, 80.07; H, 10.54; found C, 79.98; H, 10.52. MALDI TOF: m/z 660.523 ($[M+H]^+$, calcd 660.536).

(3*β*)-28-Oxo-28-[(2-thienylmethyl)amino]olean-12-en-3-yl acetate (**7e**)

Yield: 0.51 g, 86%, white crystals, mp 230–232 °C. $[\alpha]_D^{20}$ + 20.7 (*c* 0.88, $CHCl_3$); IR (KBr) ν_{max} 2946, 2876, 1732, 1647, 1511, 1465, 1368, 1247, 1149, 1027, 827, 755, 696, 665, 609 cm^{-1}; ^1H NMR ($CDCl_3$, 500 MHz) *δ* 7.21 (1H, d, *J* = 4.5 Hz, thienyl), 6.95–6.93 (2H, m, thienyl), 6.28 (1H, m, NH), 5.34 (1H, m, H-12), 4.72 (1H, dd, ^2J = 15.5 Hz, ^3J = 6.5 Hz, $C\underline{H_2}NH$), 4.48 (1H, m, H-3), 4.37 (1H, dd, ^2J =

15.5 Hz, ^3J = 4.5 Hz, C<u>H</u>$_2$NH), 2.56–0.81 (23H, m), 2.04 (3H, m, CH_3CO), 1.15 (3H, s, H-27), 0.91 (3H, s, H-25), 0.90 (6H, s, H-29, H-30), 0.86 (6H, s, H-23, H-24), 0.68 (3H, s, H-26); ^{13}C NMR ($CDCl_3$, 125 MHz) δ 177.9 (C-28), 170.9 (CH$_3$<u>C</u>O), 144.7 (C-13), 141.1 (thienyl), 126.8 (thienyl), 125.8 (thienyl), 125.0 (thienyl), 122.9 (C-12), 80.8 (C-3), 55.2 (C-5), 47.5 (C-9), 46.6 (C-19), 46.3 (C-17), 42.2, 42.0 (C-14, C-18), 39.3 (C-8), 38.5 (<u>C</u>H$_2$NH), 38.1 (C-1), 37.7 (C-4), 36.8 (C-10), 34.1 (C-21), 32.9 (C-29), 32.4, 32.3 (C-7, C-22), 30.7 (C-20), 28.0 (C-23), 27.3 (C-15), 25.7 (C-27), 23.8 (C-16), 23.6, 23.5 (C-2, C-11, C-30), 21.3 (<u>C</u>H$_3$CO), 18.2 (C-6), 16.7 (C-24, C-26), 15.4 (C-25); anal. calcd for $C_{37}H_{55}NO_3S$: C, 74.83; H, 9.33; found C, 74.77; H, 9.30. MALDI TOF: m/z 594.274 ($[M+H]^+$, calcd 594.398), 616.264 ($[M+Na]^+$, calcd 616.380), 632.218 ($[M+K]^+$, calcd 632.354).

(3*β*)-28-Oxo-28-(1,3-thiazol-2-ylamino)olean-12-en-3-yl acetate (**7f**)

Yield: 0.47 g, 81%, white crystals, mp 268–270 °C. $[\alpha]_D^{23}$ + 43.0 (*c* 0.84, $CHCl_3$); IR (KBr) ν_{max} 2946, 2930, 2876, 1733, 1675, 1532, 1474, 1367, 1318, 1247, 1163, 1027, 1009, 985, 827, 755, 703, 652, 622 cm^{-1}; ^{1}H NMR ($CDCl_3$, 500 MHz) δ 7.42 (1H, d, *J* = 3.6 Hz, thiazolyl), 7.29 (1H, s, NH), 6.94 (1H, d, *J* = 3.2 Hz, thiazolyl), 5.56 (1H, m, H-12), 4.48 (1H, m, H-3), 2.77–0.81 (23H, m), 2.04 (3H, m, CH_3CO), 1.19 (3H, s, H-27), 0.94 (3H, s, H-25), 0.93 (3H, s, H-30), 0.90 (3H, s, H-29), 0.85 (3H, s, H-23), 0.83 (3H, s, H-24), 0.63 (3H, s, H-26); ^{13}C NMR ($CDCl_3$, 125 MHz) δ 175.9 (C-28), 170.9 (CH$_3$<u>C</u>O), 158.1 (thiazolyl), 143.8 (C-13), 137.5 (thiazolyl), 124.0 (C-12), 113.5 (thiazolyl), 80.8 (C-3), 55.2 (C-5), 47.5 (C-9), 46.7 (C-17), 46.4 (C-19), 41.9 (C-14), 41.8 (C-18), 39.3 (C-8), 38.2 (C-1), 37.7 (C-4), 36.8 (C-10), 33.9 (C-21), 32.9 (C-29), 32.3, 32.2 (C-7, C-22), 30.7 (C-20), 27.9 (C-23), 27.3 (C-15), 25.8 (C-27), 24.1 (C-16), 23.6, 23.5 (C-2, C-11, C-30), 21.3 (<u>C</u>H$_3$CO), 18.1 (C-6), 16.6 (C-24), 16.4 (C-26), 15.4 (C-25); anal. calcd for $C_{35}H_{52}N_2O_3S$: C, 72.37; H, 9.02; found C, 72.29; H, 8.99. MALDI TOF: m/z 603.253 ($[M+Na]^+$, calcd 603.359).

(3*β*)-28-Oxo-28-(pyridin-4-ylamino)olean-12-en-3-yl acetate (**7g**)

Yield: 0.46 g, 80%, white crystals, mp 94–96 °C. $[\alpha]_D^{23}$ + 21.3 (*c* 0.85, $CHCl_3$); IR (KBr) ν_{max} 2948, 2930, 2873, 1732, 1695, 1588, 1506, 1472, 1413, 1369, 1327, 1248, 1179, 1028, 826, 755, 664, 601, 539 cm^{-1}; ^{1}H NMR ($CDCl_3$, 400 MHz) δ 8.44 (2H, d, *J* = 4.8 Hz, pyridyl), 8.04 (1H, s, NH), 7.47 (2H, d, *J* = 5.6 Hz, pyridyl), 5.56 (1H, m, H-12), 4.47 (1H, m, H-3), 2.70–0.80 (23H, m), 2.04 (3H, m, CH_3CO), 1.19 (3H, s, H-27), 0.93 (6H, s, H-29, H-30), 0.90 (3H, s, H-25), 0.84 (3H, s, H-23), 0.83 (3H, s, H-24), 0.65 (3H, s, H-26); ^{13}C NMR ($CDCl_3$, 100 MHz) δ 177.6 (C-28), 171.0 (CH$_3$<u>C</u>O), 150.2 (pyridyl), 145.4 (pyridyl), 144.8 (C-13), 123.6 (C-12), 113.7 (pyridyl), 80.8 (C-3), 55.1 (C-5), 47.6 (C-9), 47.4 (C-17), 46.5 (C-19), 42.4, 42.1 (C-14, C-18), 39.3 (C-8), 38.2 (C-1), 37.6 (C-4), 36.8 (C-10), 34.1 (C-21), 32.9 (C-29), 32.2 (C-7, C-22), 30.7 (C-20), 27.9 (C-23), 27.3 (C-15), 25.7 (C-27), 24.1 (C-16), 23.7, 23.5 (C-2, C-11, C-30), 21.3 (<u>C</u>H$_3$CO), 18.0 (C-6), 16.8 (C-26), 16.6 (C-24), 15.4 (C-25); anal. calcd for $C_{37}H_{54}N_2O_3$: C, 77.31; H, 9.47; found C, 76.89; H, 9.39. HRMS (ESI-TOF): calcd for $C_{37}H_{55}N_2O_3$ $[M+H]^+$ 575.4213, found 575.4203.

(3*β*)-28-[(5-Methylisoxazol-3-yl)amino]-28-oxoolean-12-en-3-yl acetate (**7h**)

Yield: 0.47 g, 81%, white crystals, mp 99–101 °C. $[\alpha]_D^{21}$ + 31.6 (*c* 0.82, $CHCl_3$); IR (KBr) ν_{max} 2947, 2876, 1733, 1694, 1619, 1539, 1472, 1419, 1369, 1247, 1181, 1028, 1008, 986, 861, 802, 756, 666, 610 cm^{-1}; ^1H NMR ($CDCl_3$, 400 MHz) δ 8.40 (1H, s, NH), 6.72 (1H, s, isoxazolyl), 5.50 (1H, m, H-12), 4.49 (1H, m, H-3), 2.72–0.70 (23H, m), 2.39 (3H, m, CH_3), 2.05 (3H, m, CH_3CO), 1.18 (3H, s, H-27), 0.93 (6H, s, H-29, H-30), 0.92 (3H, s, H-25), 0.86 (3H, s, H-23), 0.84 (3H, s, H-24), 0.70 (3H, s, H-26); ^{13}C NMR ($CDCl_3$, 100 MHz) δ 176.6 (C-28), 171.0 ($CH_3\underline{C}O$), 169.7 (isoxazolyl), 158.1 (isoxazolyl), 143.9 (C-13), 123.8 (C-12), 96.6 (isoxazolyl), 80.8 (C-3), 55.2 (C-5), 47.5 (C-9), 47.3 (C-17), 46.5 (C-19), 41.9 (C-14, C-18), 39.3 (C-8), 38.1 (C-1), 37.6 (C-4), 36.8 (C-10), 34.0 (C-21), 32.9 (C-29), 32.3, 32.2 (C-7, C-22), 30.7 (C-20), 27.9 (C-23), 27.3 (C-15), 25.8 (C-27), 23.9 (C-16), 23.5 (C-2, C-11, C-30), 21.3 ($\underline{C}H_3CO$), 18.0 (C-6), 16.6 (C-24, C-26), 15.4 (C-25), 12.7 (CH_3); anal. calcd for $C_{36}H_{54}N_2O_3$: C, 71.85; H, 9.04; found C, 70.89; H, 9.01. HRMS (ESI-TOF): calcd for $C_{36}H_{54}N_2O_3Na$ $[M+Na]^+$ 601.3981, found 601.3970.

(3*β*)-28-Oxo-28-(quinolin-8-ylamino)olean-12-en-3-yl acetate (**7i**)

Yield: 0.49 g, 80%, white crystals, mp 246–248 °C. $[\alpha]_D^{21}$ - 3.4 (*c* 0.80, $CHCl_3$); IR (KBr) ν_{max} 2947, 2876, 1732, 1669, 1527, 1486, 1459, 1436, 1384, 1325, 1247, 1027, 985, 899, 826, 792, 666, 597 cm^{-1}; ^1H NMR ($CDCl_3$, 400 MHz) δ 10.37 (1H, s, NH), 8.86 (1H, d, *J* = 6.8 Hz, quinolinyl), 8.80 (1H, d, *J* = 3.2 Hz, quinolinyl), 8.13 (1H, d, *J* = 8.0 Hz, quinolinyl), 7.53-7.42 (3H, m, quinolinyl), 5.72 (1H, m, H-12), 4.48 (1H, m, H-3), 3.02-0.70 (23H, m), 2.03 (3H, m, CH_3CO), 1.21 (3H, s, H-27), 0.99 (3H, s, H-30), 0.96 (3H, s,H-29), 0.83 (3H, s, H-23), 0.77 (3H, s, H-24), 0.74 (3H, s, H-25), 0.49 (3H, s, H-26); ^{13}C NMR ($CDCl_3$, 100 MHz) δ 176.9 (C-28), 170.8 ($CH_3\underline{C}O$), 147.1 (quinolinyl), 143.0 (C-13), 138.8 (quinolinyl), 136.2 (quinolinyl), 134.8 (quinolinyl), 127.8, 127.4 (quinolinyl), 123.8 (C-12), 121.3, 121.0 (quinolinyl), 116.4 (quinolinyl), 80.7 (C-3), 55.1 (C-5), 47.9 (C-9), 47.4 (C-17), 46.6 (C-19), 42.1, 41.7 (C-14, C-18), 39.3 (C-8), 38.0 (C-1), 37.5 (C-4), 36.7 (C-10), 34.2 (C-21), 32.9 (C-29), 32.8 (C-22), 32.3 (C-7), 30.7 (C-20), 27.9 (C-23), 27.4 (C-15), 25.8 (C-27), 24.0 (C-16), 23.6, 23.4 (C-2, C-11, C-30), 21.2 ($\underline{C}H_3CO$), 17.9 (C-6), 16.5 (C-24), 16.2 (C-26), 15.2 (C-25); anal. calcd for $C_{41}H_{56}N_2O_3$: C, 78.80; H, 9.03; found C, 78.39; H, 8.96. HRMS (ESI-TOF): calcd for $C_{41}H_{57}N_2O_3$ $[M+H]^+$ 625.4369, found 625.4339, $C_{41}H_{56}N_2O_3Na$ $[M+Na]^+$ 647.4188, found 647.4156, $C_{41}H_{56}N_2O_3K$ $[M+Na]^+$ 663.3928, found 663.3888.

Method of removing acyl group using sodium hydroxide solution.

Aqueous solution of sodium hydroxide (0.9 mL, 4 mol L^{-1}) was added to a 10 of **7a-j** in tetrahydrofuran (5 mL) and methanol (5 mL), the mixture was stirred at rt for 5 h, then poured into water (40 mL) and extracted with dichloromethane (3 × 30 mL). The extract was washed with brine, dried over sodium sulfate, evaporated, and the residue purified by column chromatography on silica gel, using hexane/ethyl acetate (3:1 to 1:3) mixture as mobile phase.

(3*β*)-3-Hydroxy-*N*-(1-methyl-1*H*-pyrazol-5-yl)olean-12-en-28-amide (**8a**)

Yield: 0.52 g, 97%, white crystals, mp 236–238 °C. $[\alpha]_D^{22}$ + 48.6 (*c* 0.64, $CHCl_3$); IR (KBr) ν_{max} 2945, 2928, 2872, 1671, 1558, 1455, 1386, 1182, 1029, 927, 755, 656 cm^{-1}; ^1H NMR ($CDCl_3$, 500 MHz) δ 7.68 (1H, br s, NH), 7.37 (1H, m, pyrazolyl), 6.21 (1H, m, pyrazolyl), 5.49 (1H, m, H-12), 3.67 (3H, s, NCH_3), 3.20 (1H, m, H-3), 2.69–0.71 (23H, m), 1.20 (3H, s, H-27), 0.98 (3H, s, H-23), 0.94 (6H, s, H-29, H-30), 0.89 (3H, s, H-25), 0.77 (6H, s, H-24, H-26); ^{13}C NMR ($CDCl_3$, 125 MHz) δ 176.7 (C-28), 144.8 (C-13), 138.3 (pyrazolyl), 135.78 (pyrazolyl), 123.8 (C-12), 99.2 (pyrazolyl), 78.7 (C-3), 55.1 (C-5), 47.4 (C-9), 47.3 (C-17), 46.5 (C-19), 42.6 (C-14), 42.2 (C-18), 39.3 (C-8), 38.8 (C-4), 37.6 (C-1), 36.9 (C-10), 35.5 (NCH_3), 34.0 (C-21), 32.9 (C-29), 32.6 (C-22), 32.3 (C-7), 30.7 (C-20), 28.1 (C-23), 27.3 (C-15), 27.1 (C-2), 25.7 (C-27), 23.9 (C-16), 23.6 (C-11, C-30), 18.2 (C-6), 17.2 (C-26), 15.6 (C-24), 15.4 (C-25); anal. calcd for $C_{34}H_{53}N_3O_2$: C, 76.22; H, 9.97; found C, 76.11; H, 9.90. MALDI TOF: m/z 536.250 ($[M+H]^+$, calcd 536.422).

N-(1,5-Dimethyl-3-oxo-2-phenyl-2,3-dihydro-1*H*-pyrazol-4-yl)-3-hydroxyolean-12-en-28-amide (**8b**)

Yield: 0.61 g, 95%, white solid. $[\alpha]_D^{19}$ + 22.3 (*c* 0.84, $CHCl_3$); IR (KBr) ν_{max} 2927, 2865, 1654, 1595, 1497, 1457, 1363, 1281, 1207, 1106, 1038, 1030, 1008, 996, 757, 702, 664, 588 cm^{-1}; ^1H NMR ($CDCl_3$, 500 MHz) δ 7.47–7.25 (6H, m, Ph, NH), 5.46 (1H, m, H-12), 3.21 (1H, t, J = 8.5 Hz, H-3), 3.05 (3H, s, NCH_3), 2.87–0.73 (23H, m), 2.29 (3H, s, CCH_3), 1.20 (3H, s, H-27), 0.99 (3H, s, H-23), 0.96 (3H, s, H-30), 0.93 (3H, s, H-29), 0.91 (3H, s, H-25), 0.85 (3H, s, H-26), 0.78 (3H, s, H-24); ^{13}C NMR ($CDCl_3$, 125 MHz) δ 176.9 (C-28), 161.7 (C=O), 148.5 (CH=), 143.7 (C-13), 134.9 (Ph), 129.2 (Ph), 126.6 (Ph), 123.7 (Ph), 123.4 (C-12), 109.5 (CH=), 78.9 (C-3), 55.2 (C-5), 47.6 (C-9), 47.2 (C-17), 46.6 (C-19), 42.0 (C-14, C-18), 39.4 (C-8), 38.8 (C-4), 38.5 (C-1), 37.0 (C-10), 36.5 (NCH_3), 34.2 (C-21), 33.2 (C-22), 33.0 (C-29), 32.7 (C-7), 30.7 (C-20), 28.1 (C-23), 27.5 (C-15), 27.2 (C-2), 25.8 (C-27), 23.9 (C-16), 23.6 (C-30), 23.5 (C-11), 18.3 (C-6), 17.3 (C-26), 15.6 (C-24), 15.4 (C-25), 13.0 (C<u>C</u>H$_3$); anal. calcd for $C_{41}H_{59}N_3O_3$: C, 76.71; H, 9.26; found C, 76.65; H, 9.21. MALDI TOF: m/z 642.492 ($[M+H]^+$, calcd 642.463), 664.452 ($[M+Na]^+$, calcd 664.445), 680.394 ($[M+K]^+$, calcd 680.419).

(3*β*)-*N*-1-Adamantyl-3-hydroxyolean-12-en-28-amide (**8c**)

Yield: 0.57 g, 97%, white solid. $[\alpha]_D^{18}$ + 27.0 (*c* 0.86, $CHCl_3$); IR (KBr) ν_{max} 2909, 2851, 1644, 1511, 1456, 1386, 1359, 1293, 1243, 1186, 1030, 997, 754, 664, 597 cm^{-1}; ^1H NMR ($CDCl_3$, 500 MHz) δ 5.53 (1H, br s, NH), 5.34 (1H, m, H-12), 3.23 (1H, m, H-3), 2.53–0.73 (23H, m), 2.05 (3H, m, adamantyl), 1.97, 1.67 (12H, m, adamantyl), 1.16 (3H, s, H-27), 1.00 (3H, s, H-23), 0.95 (3H, s, H-25), 0.90 (9H, s, H-26, H-29, H-30), 0.79 (3H, s, H-24); ^{13}C NMR ($CDCl_3$, 125 MHz) δ 176.9 (C-28), 144.8 (C-13), 122.4 (C-12), 78.9 (C-3), 55.2 (C-5), 51.4 (adamantyl), 47.6 (C-9), 46.9 (C-19), 46.5 (C-17), 42.4, 42.3 (C-14, C-18), 41.4 (adamantyl), 39.5 (C-8), 38.8 (C-4), 38.6 (C-1), 36.9 (C-10), 36.5 (adamantyl), 34.3 (C-21), 33.0 (C-22, C-29), 32.7 (C-7), 30.7 (C-20), 29.5 (adamantyl), 28.1 (C-23), 27.4 (C-15), 27.2 (C-2), 25.5 (C-27), 23.9 (C-16), 23.6 (C-11, C-30), 18.3 (C-6), 17.8 (C-26), 15.6 (C-24), 15.5 (C-25); anal. calcd for $C_{40}H_{63}NO_3$: C, 81.44; H, 10.76; found C, 81.37; H, 10.74. MALDI TOF: m/z 590.503 ($[M+H]^+$, calcd 590.494), 612.484 ($[M+Na]^+$, calcd 612.476), 628.449 ($[M+K]^+$, calcd 628.449).

(3β)-*N*-[1-(1-Adamantyl)ethyl]-3-hydroxyolean-12-en-28-amide (**8d**)

Yield: 0.59 g, 95%, white solid. $[\alpha]_D^{26}$ + 38.4 (*c* 0.82, $CHCl_3$); IR (KBr) ν_{max} 2925, 2906, 2849, 1649, 1502, 1451, 1385, 1364, 1190, 1094, 1029, 997, 754, 665, 601 cm^{-1}; ^1H NMR ($CDCl_3$, 500 MHz) δ 5.50 (5.49) (1H, br s, NH), 5.33 (1H, m, H-12), 3.64 (1H, m, CH), 3.21 (1H, m, H-3), 2.83–0.73 (23H, m), 1.99–1.96 (3H, m, adamantyl), 1.72–1.68 (1.64–1.60) (6H, m, adamantyl), 1.56–1.53 (1.44–1.40) (6H, m, adamantyl), 1.16 (3H, s, H-27), 1.01 (0.99) (3H, m, CH_3), 0.99 (3H, s, H-23), 0.93 (3H, s, H-30), 0.91 (6H, s, H-25, H-29), 0.83 (3H, s, H-26), 0.78 (3H, s, H-24); ^{13}C NMR ($CDCl_3$, 125 MHz) δ 176.4 (C-28), 143.8 (C-13), 122.4 (C-12), 78.9 (C-3), 55.3 (C-5), 52.8 (CH), 47.6 (C-9), 46.5 (C-19), 46.3 (C-17), 42.0 (C-14, C-18), 39.4 (C-8), 38.8 (C-4), 38.7 (adamantyl), 38.5 (C-1), 37.1 (adamantyl), 37.0 (C-10), 35.7 (adamantyl), 34.2 (C-21, C-22), 33.1 (C-7, C-29), 30.7 (C-20), 28.3 (adamantyl), 28.1 (C-23), 27.4 (C-15), 27.2 (C-2), 25.7 (C-27), 23.7 (C-16), 23.6 (C-30), 23.4 (C-11), 18.3 (C-6), 17.9 (C-26), 15.6 (C-24), 15.4 (C-25), 14.5 (CH_3); anal. calcd for $C_{42}H_{67}NO_2$: C, 81.63; H, 10.93; found C, 81.58; H, 10.89. MALDI TOF: m/z 618.480 ($[M+H]^+$, calcd 618.525), 640.447 ($[M+Na]^+$, calcd 640.507).

(3β)-3-Hydroxy-*N*-(2-thienylmethyl)olean-12-en-28-amide (**8e**)

Yield: 0.53 g, 97%, white solid. $[\alpha]_D^{20}$ + 17.7 (*c* 0.75, $CHCl_3$); IR (KBr) ν_{max} 2944, 2927, 2864, 1643, 1511, 1461, 1385, 1181, 1142, 1077, 1045, 755, 697 cm^{-1}; ^1H NMR ($CDCl_3$, 500 MHz) δ 7.21 (1H, d, J = 6.0 Hz, thienyl), 6.95–6.93 (2H, m, thienyl), 6.28 (1H, m, NH), 5.35 (1H, m, H-12), 4.74 (1H, dd, ^2J = 19.0 Hz, ^3J = 8.0 Hz, $C\underline{H_2}NH$), 4.37 (1H, dd, ^2J = 15.5 Hz, ^3J = 5.0 Hz, $C\underline{H_2}NH$), 3.22 (1H, m, H-3), 2.56–0.69 (23H, m), 1.17 (3H, s, H-27), 0.99 (3H, s, H-23), 0.91 (6H, s, H-29, H-30), 0.89 (3H, s, H-25), 0.79 (3H, s, H-24), 0.69 (3H, s, H-26); ^{13}C NMR ($CDCl_3$, 125 MHz) δ 177.9 (C-28), 144.8 (C-13), 141.1 (thienyl), 126.8 (thienyl), 125.8 (thienyl), 125.0 (thienyl), 123.0 (C-12), 78.9 (C-3), 55.1 (C-5), 47.5 (C-9), 46.7 (C-19), 46.3 (C-17), 42.3, 42.0 (C-14, C-18), 39.4 (C-8), 38.8 (C-4), 38.5 (C-1, $\underline{C}H_2NH$), 36.9 (C-10), 34.1 (C-21), 33.0 (C-29), 32.4 (C-7, C-22), 30.7 (C-20), 28.1 (C-23), 27.3 (C-15), 27.2 (C-2), 25.7 (C-27), 23.8 (C-16), 23.6 (C-30), 23.5 (C-11), 18.3 (C-6), 16.8 (C-26), 15.6 (C-24), 15.4 (C-25); anal. calcd for $C_{35}H_{53}NO_2S$: C, 76.17; H, 9.68; found C, 76.09; H, 9.63. MALDI TOF: m/z 552.281 ($[M+H]^+$, calcd 552.388), 574.266 ($[M+Na]^+$, calcd 574.369), 590.220 ($[M+K]^+$, calcd 590.343).

(3β)-3-Hydroxy-*N*-1,3-thiazol-2-ylolean-12-en-28-amide (**8f**)

Yield: 0.52 g, 96%, white solid. $[\alpha]_D^{23}$ + 49 (*c* 0.86, $CHCl_3$); IR (KBr) ν_{max} 2944, 2867, 1675, 1532, 1473, 1387, 1319, 1273, 1164, 1029, 997, 827, 756, 709, 654, 622 cm^{-1}; ^1H NMR ($CDCl_3$, 500 MHz) δ 7.42 (1H, d, J = 3.5 Hz, thiazolyl), 7.28 (1H, s, NH), 6.94 (1H, d, J = 3.5 Hz, thiazolyl), 5.55 (1H, m, H-12), 3.21 (1H, m, H-3), 2.80–0.70 (23H, m), 1.18 (3H, s, H-27), 0.97 (3H, s, H-23), 0.94 (6H, s, H-29, H-30), 0.87 (3H, s, H-25), 0.76 (3H, s, H-24), 0.63 (3H, s, H-26); ^{13}C NMR ($CDCl_3$, 125 MHz) δ 176.1 (C-28), 158.4 (thiazolyl), 143.7 (C-13), 137.2 (thiazolyl), 123.9 (C-12), 113.5 (thiazolyl), 78.9 (C-3), 55.1 (C-5), 47.6 (C-9), 46.7 (C-17), 46.4 (C-19), 41.8 (C-14), 41.6 (C-18), 39.3 (C-8), 38.8 (C-4), 38.5 (C-1), 36.9 (C-10), 33.9 (C-21), 32.9 (C-29), 32.3 (C-7, C-22), 30.7 (C-20), 28.1 (C-23), 27.3 (C-15), 27.2 (C-2), 25.9 (C-27), 23.9 (C-16), 23.5 (C-11, C-30), 18.2 (C-6), 16.5 (C-26), 15.6 (C-24), 15.3 (C-

25); anal. calcd for $C_{33}H_{50}N_2O_2S$: C, 73.56; H, 9.35; found C, 73.49; H, 9.31. MALDI TOF: m/z 561.269 ($[M+Na]^+$, calcd 561.349), 577.521 ($[M+K]^+$, calcd 577.323).

(3*β*)-3-Hydroxy-*N*-pyridin-4-ylolean-12-en-28-amide (**8g**)

Yield: 0.50 g, 94%, white crystals, mp 154–156 °C. $[\alpha]_D^{23}$ + 23.9 (*c* 0.81, $CHCl_3$); IR (KBr) ν_{max} 2927, 2863, 1692, 1588, 1507, 1469, 1415, 1386, 1327, 1283, 1213, 1179, 1158, 1030, 997, 826, 755, 665, 582 cm^{-1}; ^1H NMR ($CDCl_3$, 400 MHz) *δ* 8.44 (2H, d, *J* = 5.2 Hz, pyridyl), 8.22 (1H, s, NH), 7.57 (2H, d, *J* = 5.2 Hz, pyridyl), 5.57 (1H, m, H-12), 3.21 (1H, m, H-3), 2.72–0.70 (23H, m), 1.20 (3H, s, H-27), 0.98 (3H, s, H-23), 0.94 (6H, s, H-29, H-30), 0.89 (3H, s, H-25), 0.76 (3H, s, H-24), 0.66 (3H, s, H-26); ^{13}C NMR ($CDCl_3$, 100 MHz) *δ* 177.8 (C-28), 149.3 (pyridyl), 146.1 (pyridyl), 144.7 (C-13), 123.7 (C-12), 113.8 (pyridyl), 78.8 (C-3), 55.1 (C-5), 47.7 (C-9), 47.5 (C-17), 46.6 (C-19), 42.3, 42.1 (C-14, C-18), 39.3 (C-8), 38.8 (C-4), 38.5 (C-1), 36.9 (C-10), 34.1 (C-21), 32.9 (C-29), 32.3, 32.1 (C-7, C-22), 30.7 (C-20), 28.1 (C-23), 27.3 (C-15), 27.1 (C-2), 25.8 (C-27), 24.1 (C-16), 23.6 (C-11, C-30), 18.2 (C-6), 16.8 (C-26), 15.6 (C-24), 15.4 (C-25); anal. calcd for $C_{35}H_{52}N_2O_2$: C, 78.90; H, 9.84; found C, 77.89; H, 9.49. HRMS (ESI-TOF): calcd for $C_{35}H_{52}N_2O_2$ $[M+H]^+$ 533.4107, found 533.4165, calcd for $C_{35}H_{52}N_2O_2Na$ $[M+Na]^+$ 555.3926, found 555.3987.

(3*β*)-3-Hydroxy-*N*-(5-methylisoxazol-3-yl)olean-12-en-28-amide (**8h**)

Yield: 0.49 g, 93%, white crystals, mp 104–106 °C. $[\alpha]_D^{21}$ + 34.1 (*c* 0.82, $CHCl_3$); IR (KBr) ν_{max} 2927, 2863, 1692, 1619, 1538, 1471, 1418, 1386, 1272, 1181, 1094, 1031, 908, 802, 757, 666 cm^{-1}; ^1H NMR ($CDCl_3$, 400 MHz) *δ* 8.43 (1H, s, NH), 6.72 (1H, s, isoxazolyl), 5.49 (1H, m, H-12), 3.21 (1H, m, H-3), 2.73–0.69 (23H, m), 2.38 (3H, s, CH_3), 1.18 (3H, s, H-27), 0.98 (3H, s, H-23), 0.93 (6H, s, H-29, H-30), 0.89 (3H, s, H-25), 0.77 (3H, s, H-24), 0.69 (3H, s, H-26); ^{13}C NMR ($CDCl_3$, 100 MHz) *δ* 176.5 (C-28), 169.7 (isoxazolyl), 158.1 (isoxazolyl), 143.9 (C-13), 123.8 (C-12), 96.6 (isoxazolyl), 78.9 (C-3), 55.1 (C-5), 47.6 (C-9), 47.3 (C-17), 46.5 (C-19), 41.9 (C-14, C-18), 39.3 (C-8), 38.7 (C-4), 38.5 (C-1), 36.9 (C-10), 34.1 (C-21), 32.9 (C-29), 32.3 (C-7, C-22), 30.7 (C-20), 28.1 (C-23), 27.3 (C-15), 27.1 (C-2), 25.9 (C-27), 23.9 (C-16), 23.5 (C-11, C-30), 18.2 (C-6), 16.6 (C-26), 15.5 (C-24), 15.3 (C-25), 12.7(CH_3); anal. calcd for $C_{34}H_{52}N_2O_2$: C, 76.08; H, 9.76; found C, 75.79; H, 9.65. HRMS (ESI-TOF): calcd for $C_{35}H_{52}N_2O_2Na$ $[M+Na]^+$ 559.3876, found 559.3861.

(3*β*)-3-Hydroxy-*N*-quinolin-8-ylolean-12-en-28-amide (**8i**)

Yield: 0.54 g, 92%, white crystals, mp 172–174 °C. $[\alpha]_D^{22}$ - 5.5 (*c* 0.75, $CHCl_3$); IR (KBr) ν_{max} 2945, 2926, 2864, 1658, 1529, 1487, 1424, 1385, 1325, 1261, 1204, 1165, 1029, 1009, 825, 791, 755, 667, 597 cm^{-1}; ^1H NMR ($CDCl_3$, 500 MHz) *δ* 10.39 (1H, s, NH), 8.86 (1H, d, ^3J = 9.0 Hz, ^4J = 1.0 Hz, quinolinyl), 8.81 (1H, d, ^3J = 5.0 Hz, ^4J = 1.5 Hz, quinolinyl), 8.16 (1H, d, ^3J = 10.5 Hz, ^4J = 1.5 Hz, quinolinyl), 7.53–7.42 (3H, m, quinolinyl), 5.73 (1H, m, H-12), 3.20 (1H, m, H-3), 3.03–0.69 (23H, m), 1.23 (3H, s, H-27), 1.00 (3H, s, H-30), 0.98 (3H, s, H-29), 0,96 (3H, s, H-23), 0.73 (3H, s, H-25), 0.69 (3H, s, H-24), 0.50 (3H, s, H-26); ^{13}C NMR ($CDCl_3$, 125 MHz) *δ* 177.0 (C-28), 147.7 (quinolinyl), 143.0 (C-13), 138.9 (quinolinyl), 136.3 (quinolinyl), 134.9 (quinolinyl), 127.9, 127.6 (quinolinyl), 124.1 (C-12),

121.4, 121.1 (quinolinyl), 116.5 (quinolinyl), 78.9 (C-3), 55.1 (C-5), 48.1 (C-9), 47.6 (C-17), 46.8 (C-19), 42.2, 41.8 (C-14, C-18), 39.4 (C-8), 38.7 (C-4), 38.5 (C-1), 36.9 (C-10), 34.3 (C-21), 33.1 (C-22), 32.9 (C-29), 32.4 (C-7), 30.8 (C-20), 28.1 (C-23), 27.6 (C-15), 27.2 (C-2), 25.9 (C-27), 24.2 (C-16), 23.7 (C-30), 23.5 (C-11), 18.2 (C-6), 16.3 (C-26), 15.5 (C-24), 15.3 (C-25); anal. calcd for $C_{39}H_{54}N_2O_2$: C, 80.37; H, 9.34; found C, 79.89; H, 9.25. HRMS (ESI-TOF): calcd for $C_{39}H_{54}N_2O_2Na$ $[M+Na]^+$ 605.4083, found 605.4109.

*Reaction of steroids with (5Z,9Z)-tetradeca-5,9-dienedioic acid (**4**).*

To a solution of oleanolic acid with hetero- and carbocyclic moiety (1.0 mmol) in dry dichloromethane (30 ml) was added the (5*Z*,9*Z*)-tetradeca-5.9-dienedioic acid **4** (0.51 g, 2.0 mmol) followed by *N*-[3-(methylamino)propyl]-*N'*-ethylcarbodiimide hydrochloride (0.48 g, 2.5 mmol) and 4-dimethylaminopyridine (18 mg, 0.15 mmol) under argon. The mixture was stirred at room temperature for 12 h until the reaction was complete (TLC monitoring, hexane/ethyl acetate). The mixture was diluted with H_2O (10 ml) and the CH_2Cl_2 layer was separated, dried over $MgSO_4$, and concentrated. The crude product was purified by column chromatography (silica gel) using hexane/ethyl acetate (2:1) as the elution solvent to afford conjugate of oleanolic and 5*Z*,9*Z*-dienoic acid containing a heterocyclic or carbocyclic moiety.

(5*Z*,9*Z*)-14-({(3*β*)-28-[(1-methyl-1*H*-pyrazol-5-yl)amino]-28-oxoolean-12-en-3-yl}oxy)-14-oxotetradeca-5,9-dienoic acid (**9a**)

Yield: 0.47 g, 61%, colorless waxy solid. $[\alpha]_D^{18}$ + 29.2 (*c* 0.72, $CHCl_3$); IR (KBr) ν_{max} 2926, 2855, 1731, 1700, 1558, 1456, 1385, 1260, 1181, 1142, 1077, 1022, 803, 769, 649 cm^{-1}; ^1H NMR ($CDCl_3$, 500 MHz) δ 7.65 (1H, br s, NH), 7.39 (1H, m, pyrazolyl), 6.23 (1H, m, pyrazolyl), 5.50 (1H, m, H-12), 5.45–5.32 (4H, m, CH=), 4.49 (1H, m, H-3), 3.69 (3H, s, NCH_3), 2.70–0.80 (23H, m), 2.36–2.29 (4H, m, $C\underline{H}_2CO_2$, $C\underline{H}_2CO_2H$), 2.12–2.05 (8H, m, $C\underline{H}_2CH=$), 1.74–1.67 (4H, m, CH_2), 1.21 (3H, s, H-27), 0.95 (6H, s, H-25, H-30), 0.93 (3H, s, H-29), 0.86 (6H, s, H-23, H-24), 0.78 (3H, s, H-26); ^{13}C NMR ($CDCl_3$, 125 MHz) δ 177.5 (C-28, CO_2H), 173.7 (CO_2), 144.9 (C-13), 138.3 (pyrazolyl), 133.47 (pyrazolyl), 130.2 (CH=), 130.1 (CH=), 128.9 (CH=), 123.7 (C-12), 99.2 (pyrazolyl), 80.6 (C-3), 55.1 (C-5), 47.4 (C-9), 47.3 (C-17), 46.5 (C-19), 42.6 (C-14), 42.3 (C-18), 39.4 (C-8), 38.1 (C-1), 37.7 (C-4), 36.8 (C-10), 35.4 (NCH_3), 34.5 ($\underline{C}H_2CO_2$), 34.0 (C-21), 33.4 ($\underline{C}H_2CO_2H$), 32.9 (C-29), 32.6 (C-22), 32.3 (C-7), 30.7 (C-20), 28.0 (C-23), 27.3 (C-15, $\underline{C}H_2CH=$), 26.7 ($\underline{C}H_2CH=$), 26.5 ($\underline{C}H_2CH=$), 25.7 (C-27), 25.1 (CH_2), 24.8 (CH_2), 23.9 (C-16), 23.6 (C-30), 23.5 (C-2, C-11), 18.1 (C-6), 17.2 (C-26), 16.7 (C-24), 15.4 (C-25); anal. calcd for $C_{48}H_{73}N_3O_5$: C, 74.67; H, 9.53; found C, 74.59; H, 9.51. MALDI TOF: m/z 794.533 ($[M+Na]^+$, calcd 794.545).

(5*Z*,9*Z*)-14-({28-[(1,5-Dimethyl-3-oxo-2-phenyl-2,3-dihydro-1*H*-pyrazol-4-yl)amino]-28-oxoolean-12-en-3-yl}oxy)-14-oxotetradeca-5,9-dienoic acid (**9b**)

Yield: 0.52 g, 59%, colorless waxy solid. $[\alpha]_D^{18}$ + 16.8 (*c* 0.57, $CHCl_3$); IR (KBr) ν_{max} 2942, 2871, 1702, 1617, 1587, 1498, 1457, 1364, 1315, 1242, 1144, 1106, 1075, 1031, 988, 763, 702, 648, 590 cm^{-1}; ^{1}H NMR ($CDCl_3$, 500 MHz) δ 7.47–7.27 (6H, m, Ph, NH), 5.45–5.33 (5H, m, H-12, CH=), 4.51 (1H, m, H-3), 3.07 (3H, s, NCH_3), 2.89–0.83 (23H, m), 2.35-2.29 (4H, m, C$\underline{H}_2$CO$_2$, C$\underline{H}_2$CO$_2$H), 2.27 (3H, s, CCH_3), 2.12–2.05 (8H, m, C$\underline{H}_2$CH=), 1.72–1.67 (4H, m, CH_2), 1.19 (3H, s, H-27), 0.95 (3H, s, H-25), 0.93 (6H, s, H-29, H-30), 0.87 (3H, s, H-23), 0.86 (3H, s, H-24), 0.85 (3H, s, H-26); ^{13}C NMR ($CDCl_3$, 125 MHz) δ 177.3 (CO_2H), 177.1 (C-28), 173.7 (CO_2), 161.6 (C=O), 148.9 (CH=), 143.7 (C-13), 134.5 (Ph), 129.2 (Ph), 126.7 (Ph), 124.3 (Ph), 130.2 (CH=), 129.0 (CH=), 123.4 (C-12), 109.5 (CH=), 80.8 (C-3), 55.3 (C-5), 47.5 (C-9), 47.2 (C-17), 46.5 (C-19), 41.9 (C-14), 41.8 (C-18), 39.4 (C-8), 38.1 (C-1), 37.7 (C-4), 36.9 (C-10), 36.1 (NCH_3), 34.2 (C-21, $\underline{C}$H$_2$CO$_2$), 33.4 (C-22, $\underline{C}$H$_2$CO$_2$H), 33.1 (C-29), 32.6 (C-7), 30.7 (C-20), 28.1 (C-23), 27.5 (C-15), 27.3 ($\underline{C}$H$_2$CH=), 26.6 ($\underline{C}$H$_2$CH=), 25.8 (C-27), 25.1 (CH_2), 24.8 (CH_2), 23.6, 23.5 (C-2, C-11, C-16, C-30), 18.2 (C-6), 17.3 (C-26), 16.8 (C-24), 15.5 (C-25), 12.8 (C$\underline{C}$H$_3$); anal. calcd for $C_{55}H_{79}N_3O_6$: C, 75.22; H, 9.07; found C, 75.13; H, 9.02. MALDI TOF: m/z, 900.669 ([M+Na]$^+$, calcd 900.587), 916.640 ([M+K]$^+$, calcd 916.561).

(5*Z*,9*Z*)-14-{[(3*β*)-28-(1-Adamantylamino)-28-oxoolean-12-en-3-yl]oxy}-14-oxotetradeca-5,9-dienoic acid (**9c**)

Yield: 0.51 g, 62%, colorless waxy solid. $[\alpha]_D^{25}$ + 18.5 (*c* 0.77, $CHCl_3$); IR (KBr) ν_{max} 2922, 2852, 1730, 1660, 1519, 1456, 1385, 1361, 1260, 1181, 1143, 1094, 1020, 801, 755, 668, 597 cm^{-1}; ^{1}H NMR ($CDCl_3$, 500 MHz) δ 5.57 (1H, br s, NH), 5.43–5.33 (5H, m, H-12, CH=), 4.52 (1H, m, H-3), 2.53–0.83 (23H, m), 2.38–2.30 (4H, m, C$\underline{H}_2$CO$_2$, C$\underline{H}_2$CO$_2$H), 2.11-2.04 (8H, m, C$\underline{H}_2$CH=), 2.05 (3H, m, adamantyl), 1.97 (6H, m, adamantyl), 1.72–1.66 (4H, m, CH_2), 1.67 (6H, m, adamantyl), 1.17 (3H, s, H-27), 0.98 (3H, s, H-25), 0.91 (9H, s, H-26, H-29, H-30), 0.87 (6H, s, H-23, H-24); ^{13}C NMR ($CDCl_3$, 125 MHz) δ 178.2 (CO_2H), 177.1 (C-28), 173.7 (CO_2), 144.8 (C-13), 130.3 (CH=), 130.2 (CH=), 129.0 (CH=), 128.9 (CH=), 122.3 (C-12), 80.8 (C-3), 55.2 (C-5), 51.4 (adamantyl), 47.5 (C-9), 46.9 (C-19), 46.5 (C-17), 42.4, 42.2 (C-14, C-18), 41.4 (adamantyl), 39.5 (C-8), 38.2 (C-1), 37.7 (C-4), 36.9 (C-10), 36.5 (adamantyl), 34.3 (C-21, $\underline{C}$H$_2$CO$_2$), 33.3 ($\underline{C}$H$_2$CO$_2$H), 32.9 (C-22, C-29), 32.6 (C-7), 30.7 (C-20), 29.5 (adamantyl), 28.1 (C-23), 27.3 (C-15, $\underline{C}$H$_2$CH=), 26.7 ($\underline{C}$H$_2$CH=), 26.5 ($\underline{C}$H$_2$CH=), 25.5 (C-27), 25.1 (CH_2), 24.6 (CH_2), 23.8 (C-16), 23.5 (C-2, C-11, C-30), 18.2 (C-6), 17.8 (C-26), 16.8 (C-24), 15.6 (C-25); anal. calcd for $C_{54}H_{83}NO_5$: C, 78.50; H, 10.13; found C, 78.39; H, 10.09. MALDI TOF: m/z 826.53 ([M+H]$^+$, calcd 826.635), 848.504 ([M+Na]$^+$, calcd 848.617, 864.464 ([M+K]$^+$, calcd 864.591).

(5*Z*,9*Z*)-14-[((3*β*)-28-{[1-(1-Adamantyl)ethyl]amino}-28-oxoolean-12-en-3-yl)oxy]-14-oxotetradeca-5.9-dienoic acid (**9d**)

Yield: 0.53 g, 62%, colorless waxy solid. $[\alpha]_D^{24}$ + 19.5 (*c* 0.79, $CHCl_3$); IR (KBr) ν_{max} 2927, 2850, 1730, 1651, 1515, 1454, 1385, 1364, 1243, 1095, 1019, 755, 665 cm^{-1}; ^{1}H NMR ($CDCl_3$, 500 MHz) δ 5.54 (5.52) (1H, br s, NH), 5.43–5.33 (5H, m, H-12, CH=), 4.51 (1H, t, *J* = 8.5 Hz, H-3), 3.64 (1H, m, CH), 2.82–0.83 (23H, m), 2.37–2.31 (4H, m, C$\underline{H}_2$CO$_2$, C$\underline{H}_2$CO$_2$H), 2.12–2.05 (8H, m, C$\underline{H}_2$CH=), 1.99–1.96 (3H, m, adamantyl), 1.72–1.68 (1.64–1.60) (6H, m, adamantyl), 1.73–1.66 (4H, m, CH_2), 1.56–1.53

(1.44–1.40) (6H, m, adamantyl), 1.16 (3H, s, H-27), 1.01 (0.99) (3H, d, J = 6.5 Hz, CH_3), 0.94 (6H, s, H-25, H-30), 0.91 (3H, s, H-29), 0.87 (3H, s, H-23), 0.86 (3H, s, H-24), 0.83 (3H, m, H-26); ^{13}C NMR ($CDCl_3$, 125 MHz) δ 178.3 (CO_2H), 176.6 (C-28), 173.7 (CO_2), 143.8 (C-13), 130.3 (CH=), 130.1 (CH=), 129.0 (CH=), 128.9 (CH=), 122.4 (C-12), 80.8 (C-3), 55.3 (C-5), 52.9 (CH), 47.5 (C-9), 46.6 (C-19), 46.2 (C-17), 42.1 (C-14, C-18), 39.5 (C-8), 38.6 (adamantyl), 38.1 (C-1), 37.7 (C-4), 37.1 (adamantyl), 36.9 (C-10), 35.7 (adamantyl), 34.3 ($\underline{C}H_2CO_2$), 34.2 (C-21, C-22), 33.3 ($\underline{C}H_2CO_2H$), 33.1 (C-7, C-29), 30.7 (C-20), 28.3 (adamantyl), 28.1 (C-23), 27.3 (C-15, $\underline{C}H_2$CH=), 26.7 ($\underline{C}H_2$CH=), 26.5 ($\underline{C}H_2$CH=), 25.7 (C-27), 25.1 (CH_2), 24.6 (CH_2), 23.6, 23.5 (C-2, C-16, C-30), 23.4 (C-11), 18.2 (C-6), 17.9 (C-26), 16.8 (C-24), 15.4 (C-25), 14.4 (CH_3); anal. calcd for $C_{56}H_{87}NO_3$: C, 76.67; H, 10.00; found C, 76.58; H, 9.97. MALDI TOF: m/z 854.784 ($[M+H]^+$, calcd 854.666), 876.765 ($[M+Na]^+$, calcd 876.648).

(5*Z*,9*Z*)-14-Oxo-14-({(3*β*)-28-Oxo-28-[(2-thienylmethyl)amino]olean-12-en-3-yl}oxy)tetradeca-5,9-dienoic acid (**9e**)

Yield: 0.49 g, 63%, colorless waxy solid. $[\alpha]_D^{19}$ + 15.3 (*c* 0.53, $CHCl_3$); IR (KBr) ν_{max} 2943, 2871, 1730, 1635, 1519, 1456, 1386, 1366, 1243, 1212, 1169, 1146, 1096, 1016, 853, 827, 697 cm^{-1}; ^{1}H NMR ($CDCl_3$, 500 MHz) δ 7.23 (1H, d, J = 6.0 Hz, thienyl), 6.98–6.94 (2H, m, thienyl), 6.28 (1H, m, NH), 5.45–5.35 (5H, m, H-12, CH=), 4.75 (1H, dd, ^2J = 19.0 Hz, ^3J = 8.0 Hz, C$\underline{H}_2$NH), 4.51 (1H, m, H-3), 4.38 (1H, dd, ^2J = 19.0 Hz, ^3J = 5.5 Hz, C$\underline{H}_2$NH), 2.57–0.82 (23H, m), 2.39–2.31 (4H, m, C$\underline{H}_2CO_2$, C$\underline{H}_2CO_2H$), 2.12–2.05 (8H, m, C$\underline{H}_2$CH=), 1.74–1.68 (4H, m, CH_2), 1.17 (3H, s, H-27), 0.93 (3H, s, H-25), 0.92 (6H, s, H-29, H-30), 0.88 (6H, s, H-23, H-24), 0.69 (3H, s, H-26); ^{13}C NMR ($CDCl_3$, 125 MHz) δ 178.1 (C-28, CO_2H), 173.7 (CO_2), 144.8 (C-13), 140.9 (thienyl), 130.3 (CH=), 130.2 (CH=), 129.0 (CH=), 128.9 (CH=), 126.8 (thienyl), 125.9 (thienyl), 125.1 (thienyl), 123.0 (C-12), 80.7 (C-3), 55.2 (C-5), 47.5 (C-9), 46.6 (C-19), 46.3 (C-17), 42.3, 42.0 (C-14, C-18), 39.4 (C-8), 38.6 ($\underline{C}H_2$NH), 38.1 (C-1), 37.7 (C-4), 36.8 (C-10), 34.3 ($\underline{C}H_2CO_2$), 34.1 (C-21), 33.3 ($\underline{C}H_2CO_2H$), 32.9 (C-29), 32.4, 32.3 (C-7, C-22), 30.7 (C-20), 28.1 (C-23), 27.3 (C-15, $\underline{C}H_2$CH=), 26.7 ($\underline{C}H_2$CH=), 26.5 ($\underline{C}H_2$CH=), 25.7 (C-27), 25.1 (CH_2), 24.6 (CH_2), 23.8 (C-16), 23.6, 23.5 (C-2, C-11, C-30), 18.2 (C-6), 16.7 (C-24, C-26), 15.4 (C-25); anal. calcd for $C_{49}H_{73}NO_5S$: C, 74.67; H, 9.34; found C, 74.59; H, 9.30. MALDI TOF: m/z 788.591 ($[M+H]^+$, calcd 788.529), 810.602 ($[M+Na]^+$, calcd 810.511), 826.544 ($[M+K]^+$, calcd 826.485).

(5*Z*,9*Z*)-14-Oxo-{[(3*β*)-28-oxo-28-(1,3-thiazol-2-ylamino)olean-12-en-3-yl]oxy}tetradeca-5,9-dienoic acid (**9f**)

Yield: 0.47 g, 61%, colorless waxy solid. $[\alpha]_D^{24}$ + 25 (*c* 0.87, $CHCl_3$); IR (KBr) ν_{max} 2944, 2861, 1729, 1690, 1538, 1461, 1365, 1321, 1278, 1244, 1206, 1179, 1164, 1145, 1089, 1011, 987, 917, 827, 757, 712, 652, 622 cm^{-1}; ^{1}H NMR ($CDCl_3$, 500 MHz) δ 7.38 (1H, d, J = 3.5 Hz, thiazolyl), 7.29 (1H, s, NH), 6.92 (1H, d, J = 4.0 Hz, thiazolyl), 5.46 (1H, m, H-12), 5.41–5.35 (4H, m, CH=), 4.49 (1H, m, H-3), 2.98–0.80 (23H, m), 2.37 (2H, t, J = 7.5 Hz, C$\underline{H}_2CO_2H$), 2.31 (2H, t, J = 7.5 Hz, C$\underline{H}_2CO_2$), 2.13–2.05 (8H, m, C$\underline{H}_2$CH=), 1.74-1.67 (4H, m, CH_2), 1.17 (3H, s, H-27), 0.97 (3H, s, H-30), 0.94 (3H, s, H-29), 0.89 (3H, s, H-25), 0.85 (3H, s, H-23), 0.83 (3H, s, H-24), 0.62 (3H, s, H-26); ^{13}C NMR ($CDCl_3$, 125 MHz) δ 178.6 (CO_2H), 176.1 (C-28), 173.5 (CO_2), 160.1 (thiazolyl), 143.7 (C-13), 135.7 (thiazolyl),

130.2 (CH=), 130.1 (CH=), 129.1 (CH=), 123.1 (C-12), 113.2 (thiazolyl), 80.7 (C-3), 55.2 (C-5), 47.5 (C-9), 46.9 (C-17), 46.2 (C-19), 41.7 (C-14), 40.8 (C-18), 39.3 (C-8), 38.1 (C-1), 37.7 (C-4), 36.9 (C-10), 34.3 ($\underline{C}H_2CO_2$), 34.0 (C-21), 33.7 ($\underline{C}H_2CO_2H$), 33.0 (C-29), 32.3 (C-22), 32.2 (C-7), 30.7 (C-20), 28.0 (C-23), 27.4, 27.3 (C-15, $\underline{C}H_2CH$=), 26.7 ($\underline{C}H_2CH$=), 26.6 ($\underline{C}H_2CH$=), 25.9 (C-27), 25.1 (CH_2), 24.8 (CH_2), 23.6 (C-30), 23.5 (C-2, C-11), 23.1 (C-16), 18.1 (C-6), 16.7 (C-24), 16.5 (C-26), 15.4 (C-25); anal. calcd for $C_{47}H_{70}N_2O_5S$: C, 72.83; H, 9.10; found C, 72.71; H, 9.07. MALDI TOF: m/z 775.381 ($[M+H]^+$, calcd 775.508), 797.631 ($[M+Na]^+$, calcd 797.490), 813.248 ($[M+K]^+$, calcd 813.464).

(5*Z*,9*Z*)-14-Oxo-{[(3*β*)-28-oxo-28-(pyridin-4-ylamino)olean-12-en-3-yl]oxy}tetradeca-5,9-dienoic acid (**9g**)

Yield: 0.46 g, 60%, colorless waxy solid. $[\alpha]_D^{24}$ + 25.7 (*c* 0.81, $CHCl_3$); IR (KBr) ν_{max} 2946, 2861, 1728, 1704, 1588, 1509, 1468, 1417, 1379, 1365, 1283, 1246, 1204, 1177, 1146, 1013, 829, 754, 589, 537 cm^{-1}; ^1H NMR ($CDCl_3$, 400 MHz) δ 8.48 (2H, m, pyridyl), 8.09 (1H, s, NH), 7.54 (2H, d, J = 4.8 Hz, pyridyl), 5.59 (1H, m, H-12), 5.41-5.34 (4H, m, CH=), 4.49 (1H, m, H-3), 2.71–0.80 (23H, m), 2.36 (2H, m, $C\underline{H}_2CO_2H$), 2.32 (2H, m, $C\underline{H}_2CO_2$), 2.13–2.05 (8H, m, $C\underline{H}_2CH$=), 1.74–1.67 (4H, m, CH_2), 1.21 (3H, s, H-27), 0.95 (3H, s, H-30), 0.94 (3H, s, H-29), 0.89 (3H, s, H-25), 0.85 (3H, s, H-23), 0.83 (3H, s, H-24), 0.62 (3H, s, H-26); ^{13}C NMR ($CDCl_3$, 100 MHz) δ 177.7 (C-28), 177.4 (CO_2H), 173.7 (CO_2), 149.0 (pyridyl), 146.1 (pyridyl), 144.8 (C-13), 130.2 (CH=), 130.1 (CH=), 129.1 (CH=), 128.9 (CH=), 123.7 (C-12), 113.8 (pyridyl), 80.6 (C-3), 55.1 (C-5), 47.7 (C-9), 47.4 (C-17), 46.2 (C-19), 42.4, 42.1 (C-14, C-18), 39.4 (C-8), 38.2 (C-1), 37.7 (C-4), 36.8 (C-10), 34.3 ($\underline{C}H_2CO_2$), 34.1 (C-21), 33.7 ($\underline{C}H_2CO_2H$), 32.9 (C-29), 32.2 (C-7, C-22), 30.7 (C-20), 28.0 (C-23), 27.4 (C-15, $\underline{C}H_2CH$=), 26.7 ($\underline{C}H_2CH$=), 25.7 (C-27), 25.1 (CH_2), 24.9 (CH_2), 24.1 (C-16), 23.7 (C-2), 23.6 (C-11, C-30), 18.1 (C-6), 16.7 (C-24, C-26), 15.4 (C-25); anal. calcd for $C_{49}H_{72}N_2O_5$: C, 76.52; H, 9.44; found C, 75.89; H, 9.27. MALDI TOF: m/z 775.381 ($[M+H]^+$, calcd 775.508), 797.631 ($[M+Na]^+$, calcd 797.490), 813.248 ($[M+K]^+$, calcd 813.464). HRMS (ESI-TOF): calcd for $C_{49}H_{73}N_2O_5$ $[M+H]^+$ 769.5519, found 769.5441.

(5*Z*,9*Z*)-14-({(3*β*)-28-[(5-methylisoxazol-3-yl)amino]-28-oxoolean-12-en-3-yl}oxy)-14-oxotetradeca-5,9-dienoic acid (**9h**)

Yield: 0.47 g, 61%, colorless waxy solid. $[\alpha]_D^{21}$ + 25.1 (*c* 0.82, $CHCl_3$); IR (KBr) ν_{max} 2927, 2855, 1729, 1707, 1619, 1543, 1459, 1420, 1366, 1271, 1243, 1180, 1145, 1097, 1009, 988, 910, 803, 757 cm^{-1}; ^1H NMR ($CDCl_3$, 500 MHz) δ 8.99 (1H, s, NH), 6.75 (1H, m, isoxazolyl), 5.49 (1H, m, H-12), 5.43–5.33 (4H, m, CH=), 4.50 (1H, m, H-3), 2.78–0.82 (23H, m), 2.39 (3H, s, CH_3), 2.38 (2H, t, J = 7.5 Hz, $C\underline{H}_2CO_2H$), 2.32 (2H, t, J = 7.5 Hz, $C\underline{H}_2CO_2$), 2.12–2.05 (8H, m, $C\underline{H}_2CH$=), 1.73–1.69 (4H, m, CH_2), 1.19 (3H, s, H-27), 0.94 (6H, s, H-29, H-30), 0.92 (3H, s, H-25), 0.86 (3H, s, H-23), 0.84 (3H, s, H-24), 0.69 (3H, s, H-26); ^{13}C NMR ($CDCl_3$, 125 MHz) δ 178.4 (CO_2H), 176.7 (C-28), 173.7 (CO_2), 169.7 (isoxazolyl), 158.2 (isoxazolyl), 143.9 (C-13), 130.3 (CH=), 130.1 (CH=), 129.1 (CH=), 128.9 (CH=), 123.6 (C-12), 96.7 (isoxazolyl), 80.7 (C-3), 55.2 (C-5), 47.5 (C-9), 47.4 (C-17), 46.4 (C-19), 41.9, 41.7 (C-14, C-18), 39.3 (C-8), 38.1 (C-1), 37.7 (C-4), 36.8 (C-10), 34.3 ($\underline{C}H_2CO_2$), 34.0 (C-21), 33.3 ($\underline{C}H_2CO_2H$), 32.9 (C-29), 32.3 (C-7, C-22), 30.7 (C-20), 28.0 (C-23), 27.3 (C-15, $\underline{C}H_2CH$=), 26.7

(<u>C</u>H_2CH=), 26.5 (<u>C</u>H_2CH=), 25.9 (C-27), 25.1 (CH_2), 24.6 (CH_2), 23.7 (C-16), 23.6 (C-2, C-11, C-30), 18.1 (C-6), 16.7 (C-24, C-26), 15.4 (C-25), 12.6 (CH_3); anal. calcd for $C_{48}H_{72}N_2O_6$: C, 74.57; H, 9.39; found C, 74.09; H, 9.17. HRMS (ESI-TOF): calcd for $C_{48}H_{73}N_2O_6$ $[M+H]^+$ 773.5469, found 773.5494, calcd for $C_{48}H_{72}N_2O_6Na$ $[M+Na]^+$ 795.5288, found 795.5310, calcd for $C_{48}H_{72}N_2O_6K$ $[M+K]^+$ 811.5027, found 811.5248.

(5*Z*,9*Z*)-14-Oxo-14-{[(3*β*)-28-oxo-28-(quinolin-8-ylamino)olean-12-en-3-yl]oxy}tetradeca-5,9-dienoic acid (**9i**)

Yield: 0.46 g, 59%, colorless waxy solid. $[\alpha]_D^{21}$ + 25.1 (*c* 0.82, $CHCl_3$); IR (KBr) ν_{max} 2927, 2855, 1729, 1707, 1619, 1543, 1459, 1420, 1366, 1271, 1243, 1180, 1145, 1097, 1009, 988, 910, 803, 757 cm^{-1}; ^{1}H NMR ($CDCl_3$, 500 MHz) δ 10.36 (1H, s, NH), 8.86-8.83 (2H, m, quinolinyl), 8.21 (1H, d, J = 8.4 Hz, quinolinyl), 7.57–7.47 (3H, m, quinolinyl), 5.71 (1H, m, H-12), 5.42–5.32 (4H, m, CH=), 4.50 (1H, m, H-3), 2.78–0.82 (23H, m), 2.36 (2H, t, J = 7.6 Hz, C<u>H</u>$_2CO_2$H), 2.32 (2H, t, J = 7.5 Hz, C<u>H</u>$_2CO_2$), 2.12–2.05 (8H, m, C<u>H</u>$_2$CH=), 1.73–1.69 (4H, m, CH_2), 1.23 (3H, s, H-27), 1.00 (3H, s, H-30), 0.98 (3H, s, H-29), 0.84 (3H, s, H-23), 0.79 (3H, s, H-24), 0.76 (3H, s, H-25), 0.52 (3H, s, H-26); ^{13}C NMR ($CDCl_3$, 125 MHz) δ 178.4 (CO_2H), 177.2 (C-28), 173.7 (CO_2), 147.5 (quinolinyl), 143.2 (C-13), 138.4 (quinolinyl), 136.9 (quinolinyl), 134.6 (quinolinyl), 130.4 (CH=), 130.1 (CH=), 129.1 (CH=), 128.8 (CH=), 128.1, 127.8 (quinolinyl), 123.9 (C-12), 121.3 (quinolinyl), 117.4 (quinolinyl), 80.8 (C-3), 55.2 (C-5), 48.1 (C-9), 47.5 (C-17), 46.8 (C-19), 42.2, 41.9 (C-14, C-18), 39.5 (C-8), 38.1 (C-1), 37.7 (C-4), 36.8 (C-10), 34.3 (<u>C</u>H_2CO_2, C-21), 33.2 (<u>C</u>H_2CO_2H), 33.1 (C-29), 32.8 (C-22), 32.4 (C-7), 30.8 (C-20), 28.0 (C-23), 27.6 (C-15), 27.3 (<u>C</u>H_2CH=), 26.7 (<u>C</u>H_2CH=), 26.5 (<u>C</u>H_2CH=), 25.9 (C-27), 25.1 (CH_2), 24.6 (CH_2), 24.0 (C-16), 23.7 (C-2), 23.5 (C-11, C-30), 18.1 (C-6), 16.7 (C-24), 16.4 (C-26), 15.3 (C-25); anal. calcd for $C_{53}H_{74}N_2O_5$: C, 77.71; H, 9.11; found C, 77.29; H, 9.09. HRMS (ESI-TOF): calcd for $C_{53}H_{75}N_2O_5$ $[M+H]^+$ 819.5676, found 819.5706, calcd for $C_{53}H_{74}N_2O_5Na$ $[M+Na]^+$ 841.5495, found 841.5525.

^{1}H and ^{13}C NMR Spectra of all Compounds

Figure S1. ^{13}C NMR Spectrum of compound **7a** (125 MHz, $CDCl_3$)

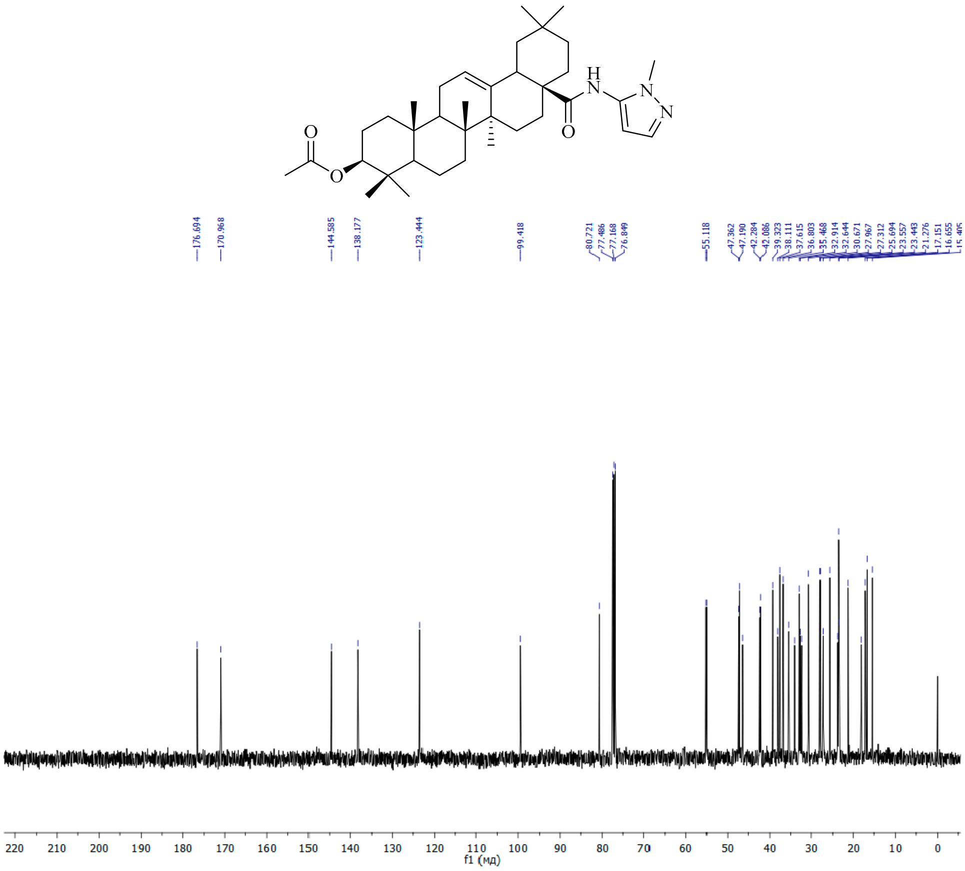

Figure S2. ^{1}H NMR Spectrum of compound **7a** (500 MHz, $CDCl_3$)

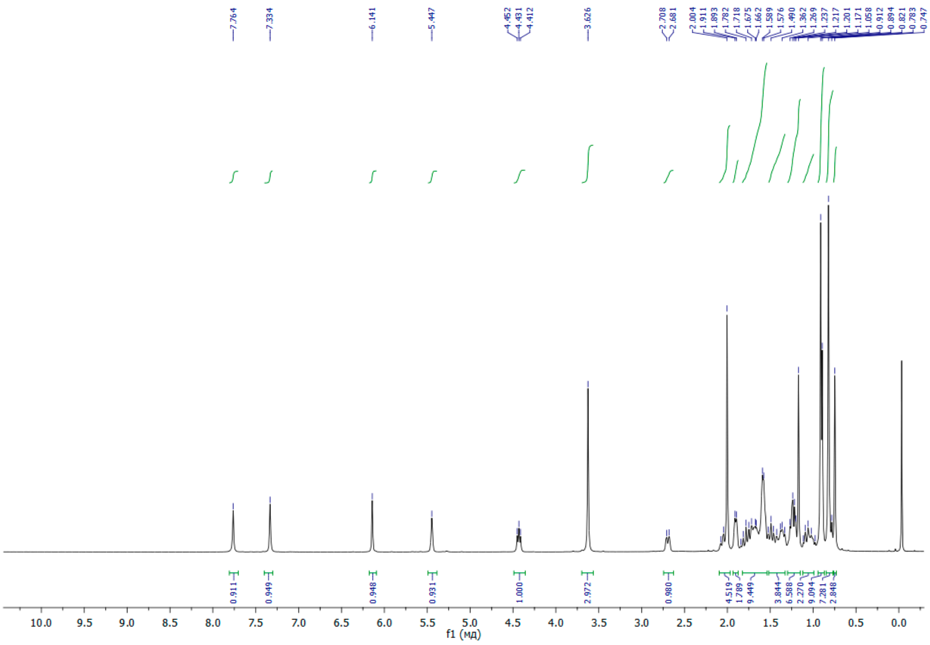

Figure S3. ^{13}C NMR Spectrum of compound **7b** (125 MHz, $CDCl_3$)

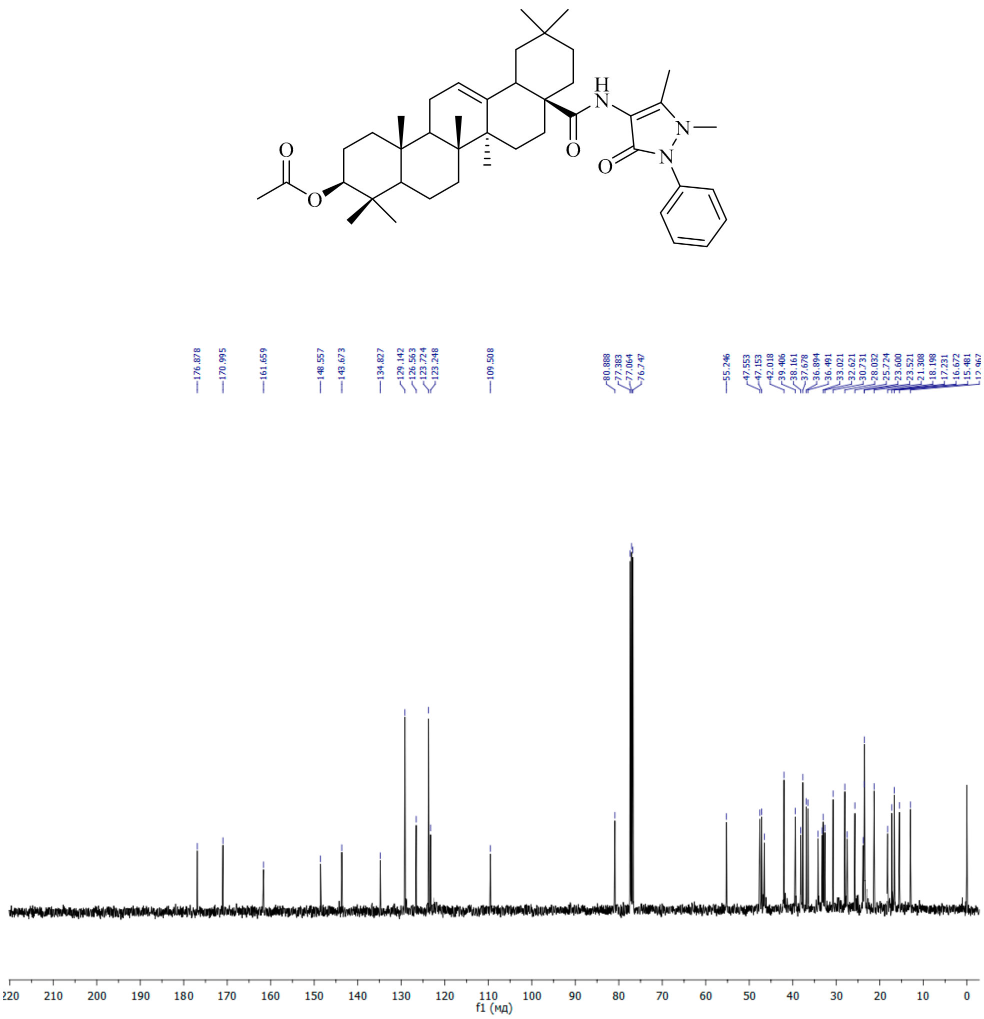

Figure S4. ^{1}H NMR Spectrum of compound **7b** (500 MHz, $CDCl_3$)

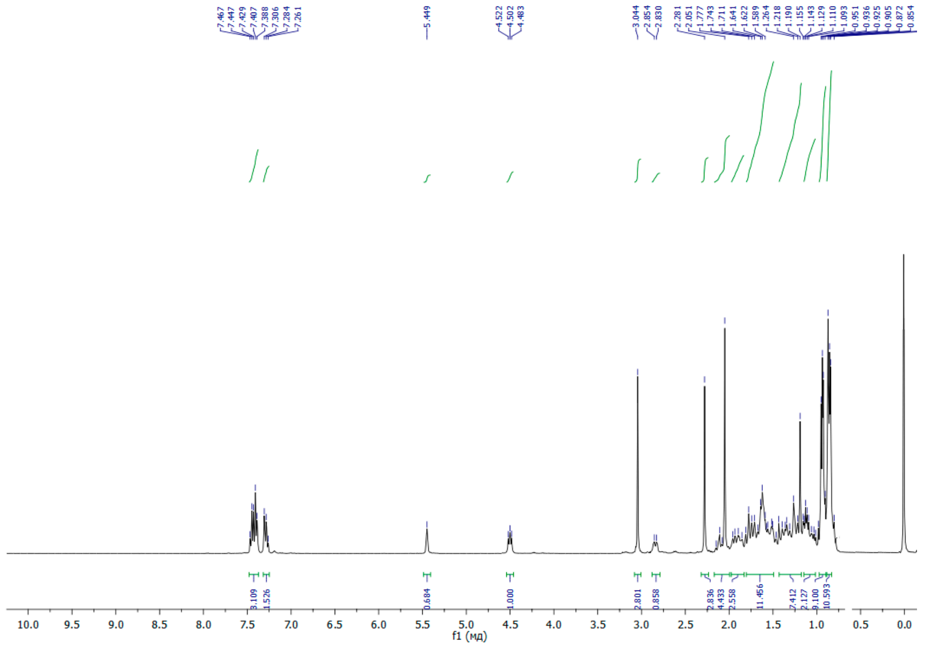

Figure S5. ^{13}C NMR Spectrum of compound **7c** (125 MHz, $CDCl_3$)

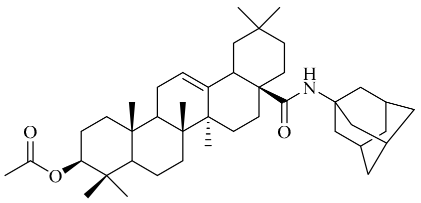

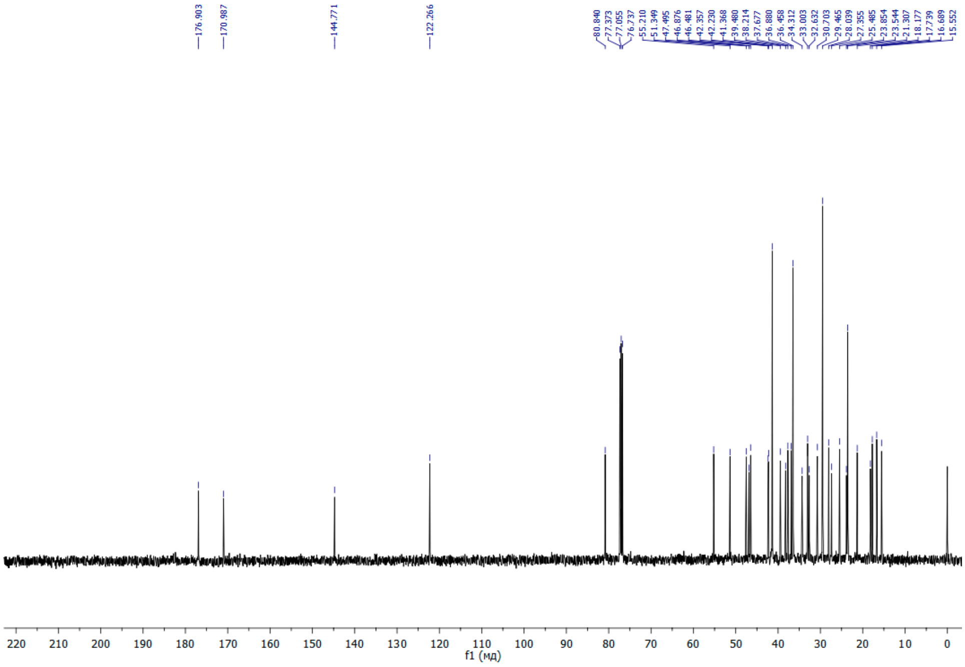

Figure S6. ^{1}H NMR Spectrum of compound **7c** (500 MHz, $CDCl_3$)

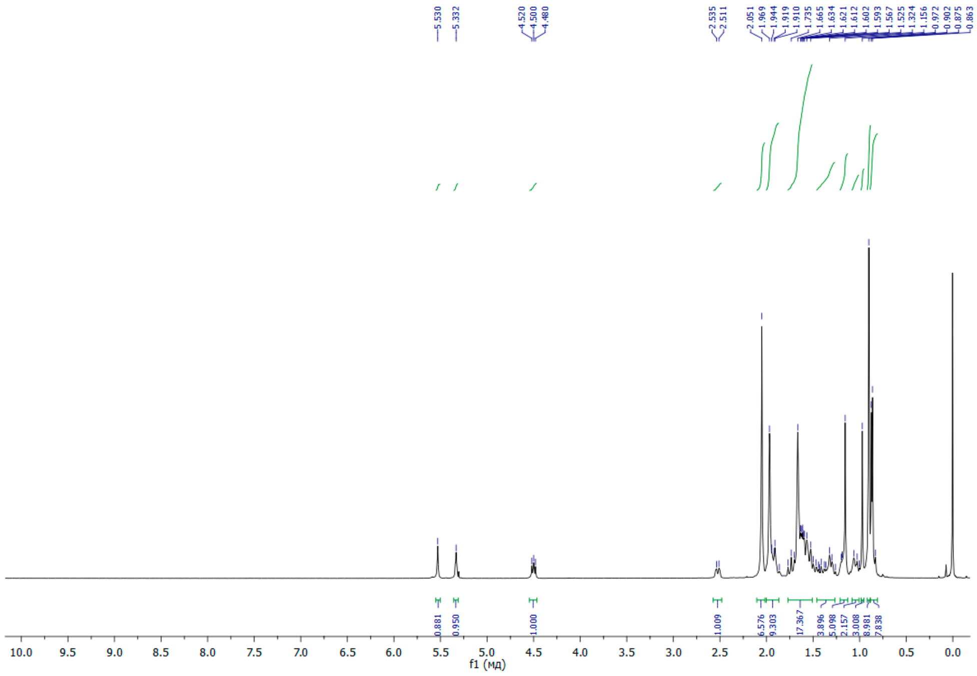

Figure S7. ^{13}C NMR Spectrum of compound **7d** (125 MHz, $CDCl_3$)

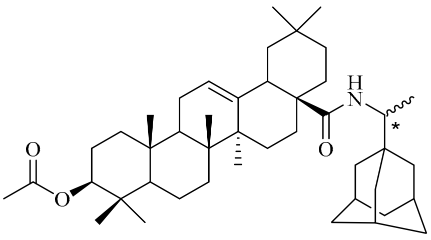

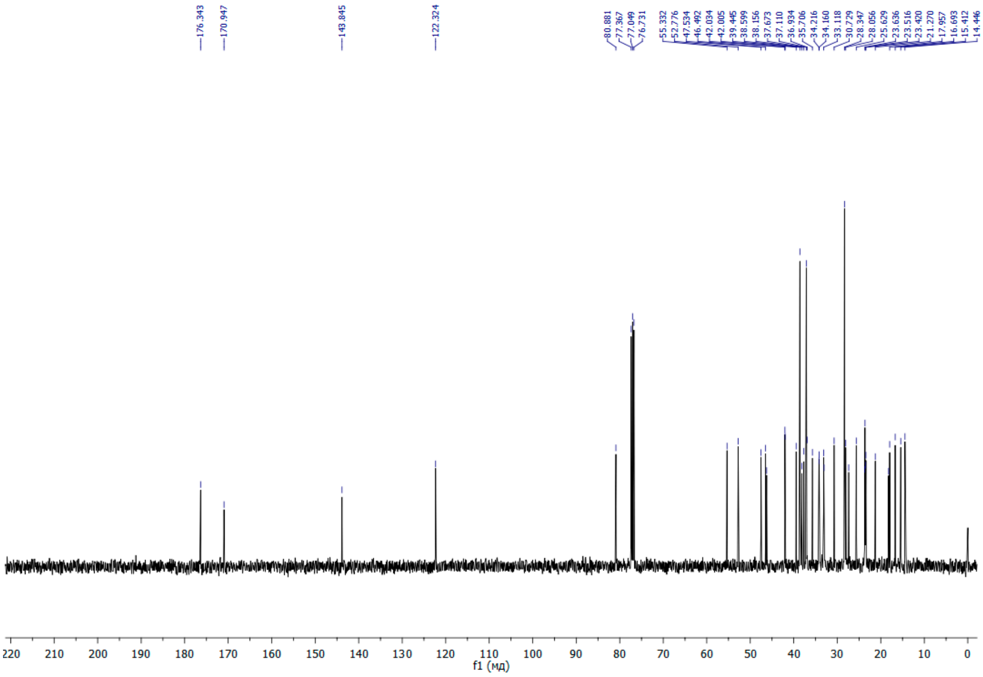

Figure S8. ^{1}H NMR Spectrum of compound **7d** (500 MHz, $CDCl_3$)

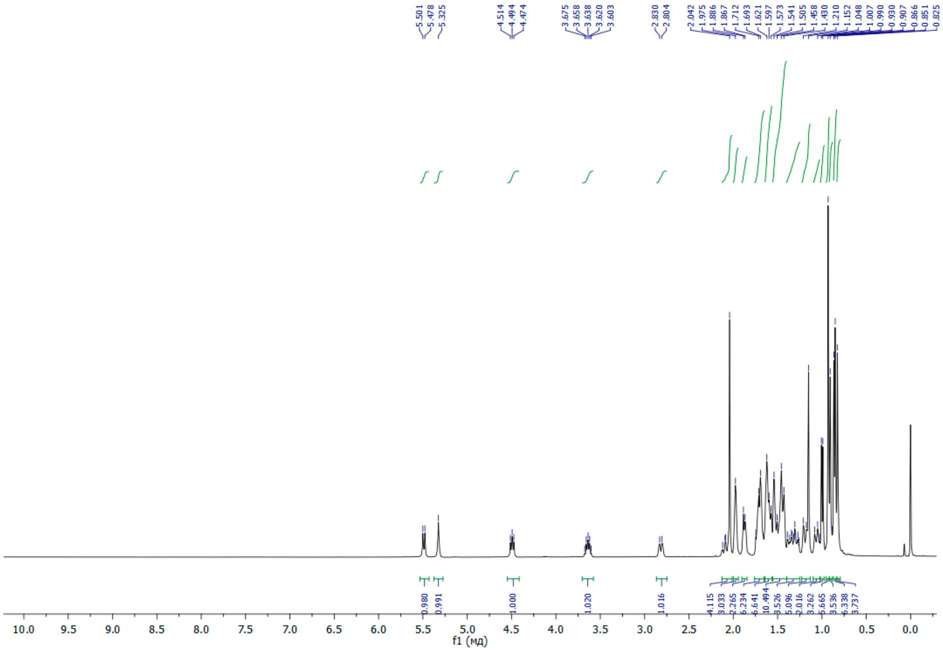

Figure S9. ^{13}C NMR Spectrum of compound **7e** (125 MHz, $CDCl_3$)

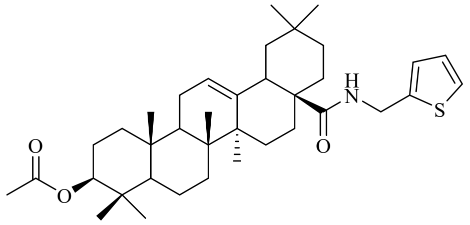

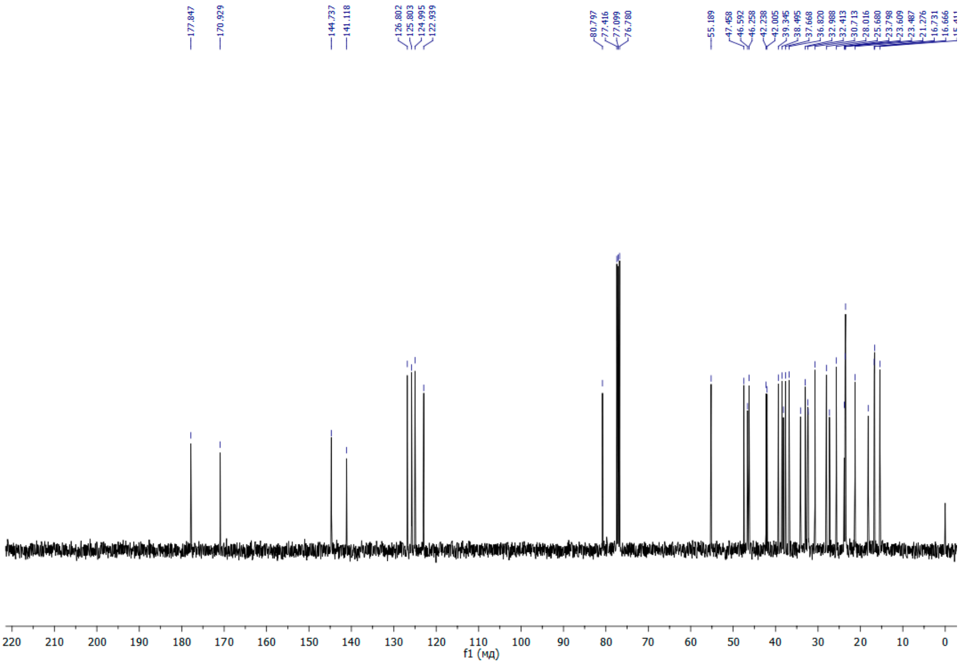

Figure S10. ^{1}H NMR Spectrum of compound **7e** (500 MHz, $CDCl_3$)

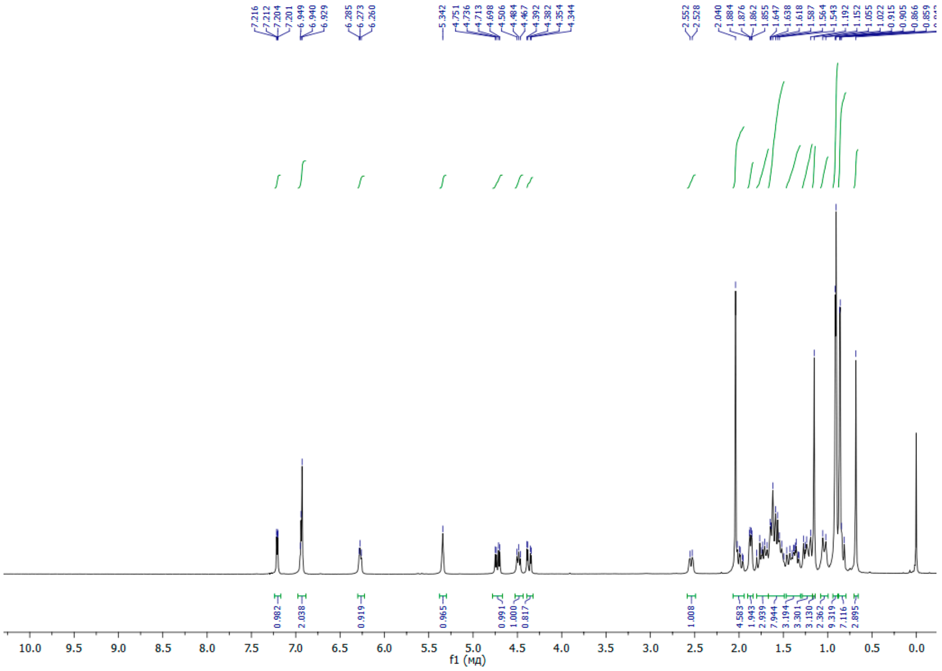

Figure S11. ^{13}C NMR Spectrum of compound **7f** (125 MHz, $CDCl_3$)

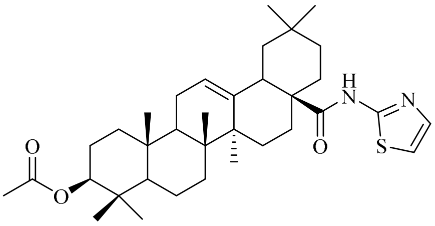

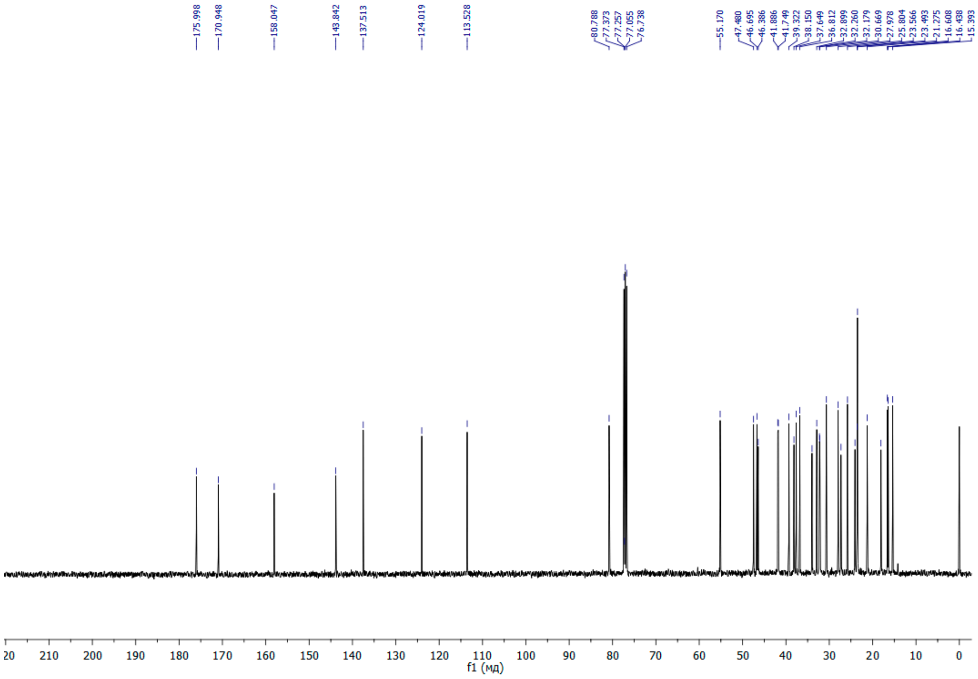

Figure S12. ^{1}H NMR Spectrum of compound **7f** (500 MHz, $CDCl_3$)

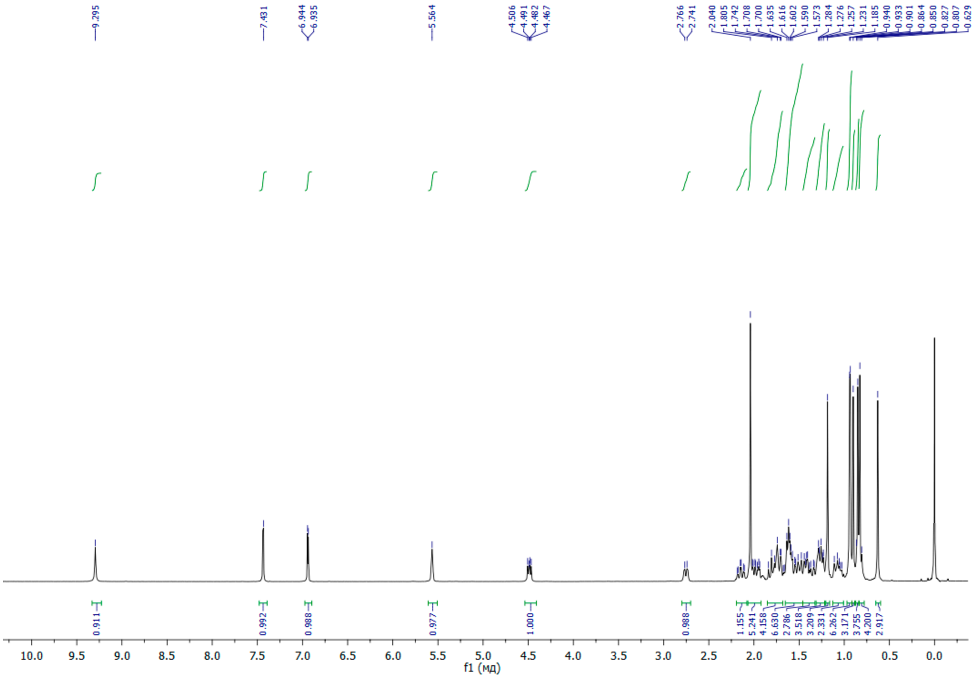

Figure S13. ^{13}C NMR Spectrum of compound **7g** (125 MHz, $CDCl_3$)

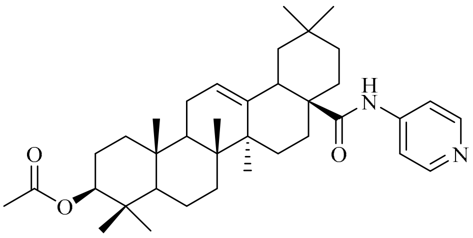

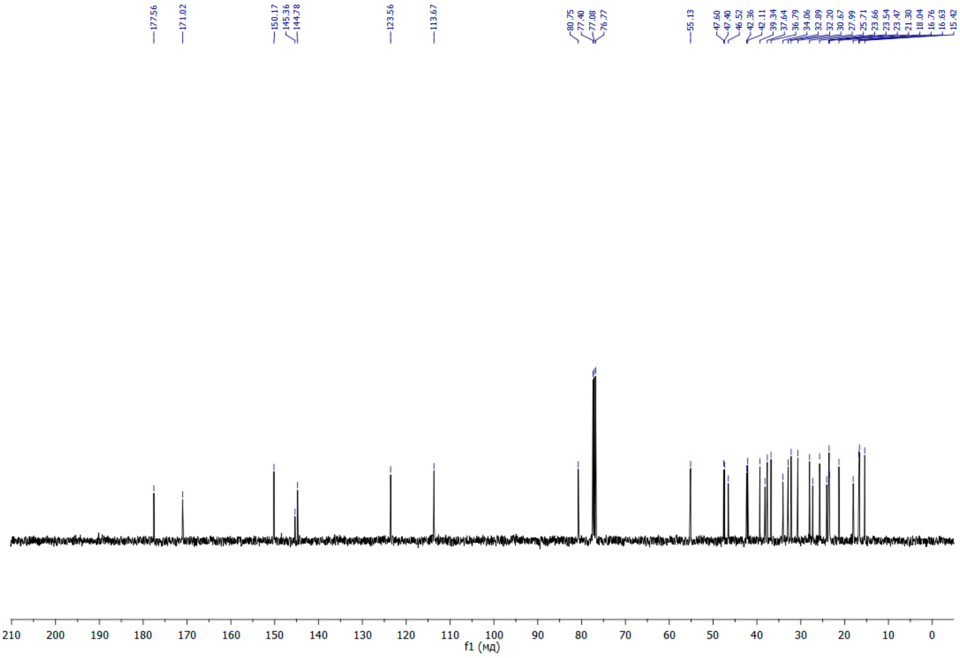

Figure S14. ^{1}H NMR Spectrum of compound **7g** (500 MHz, $CDCl_3$)

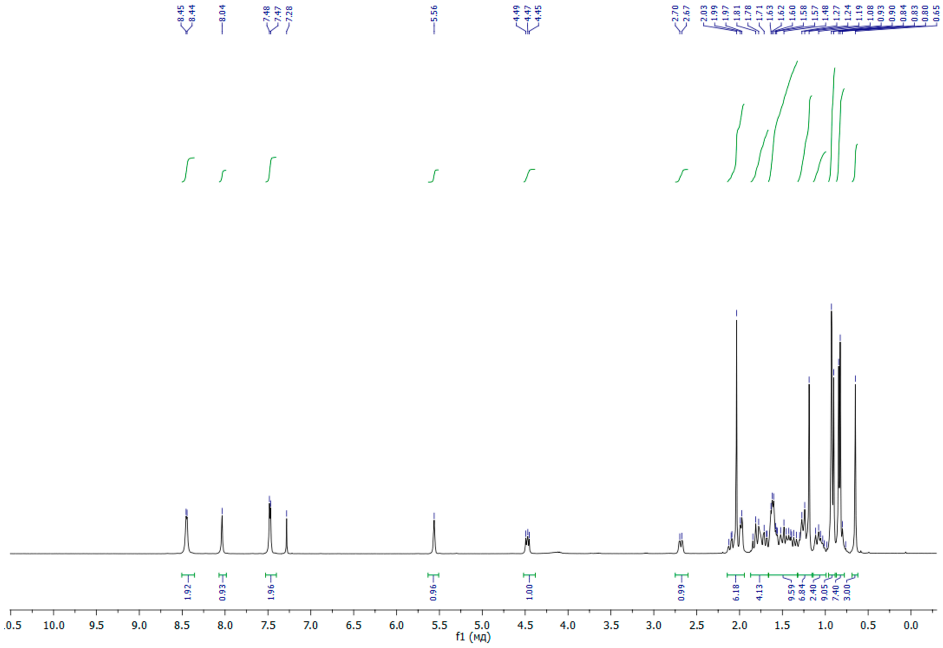

Figure S15. ^{13}C NMR Spectrum of compound **7h** (125 MHz, $CDCl_3$)

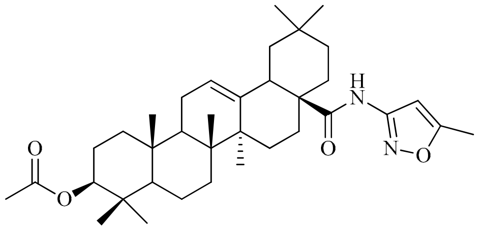

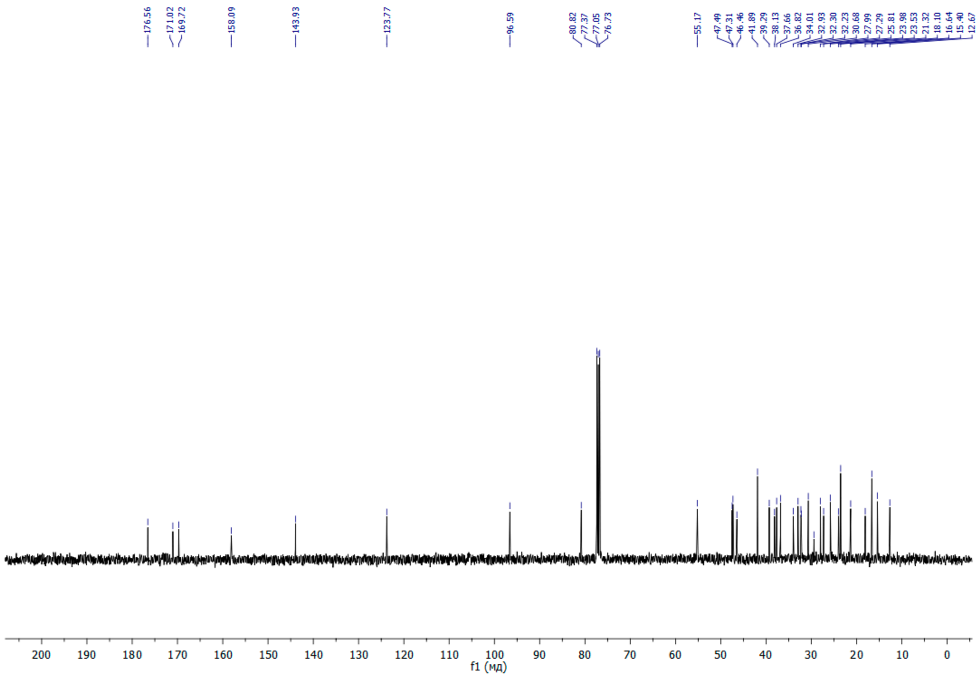

Figure S16. ^{1}H NMR Spectrum of compound **7h** (500 MHz, $CDCl_3$)

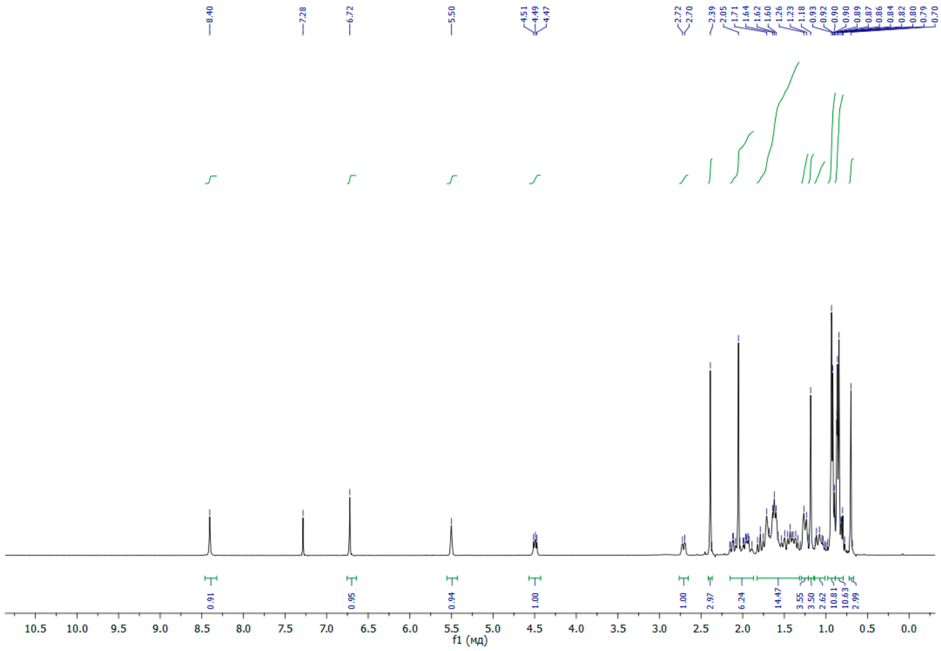

Figure S17. ^{13}C NMR Spectrum of compound **7i** (125 MHz, $CDCl_3$)

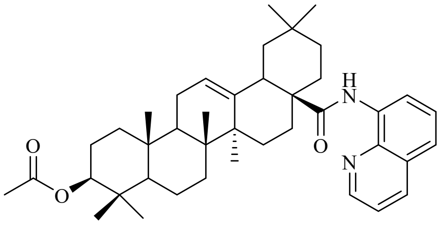

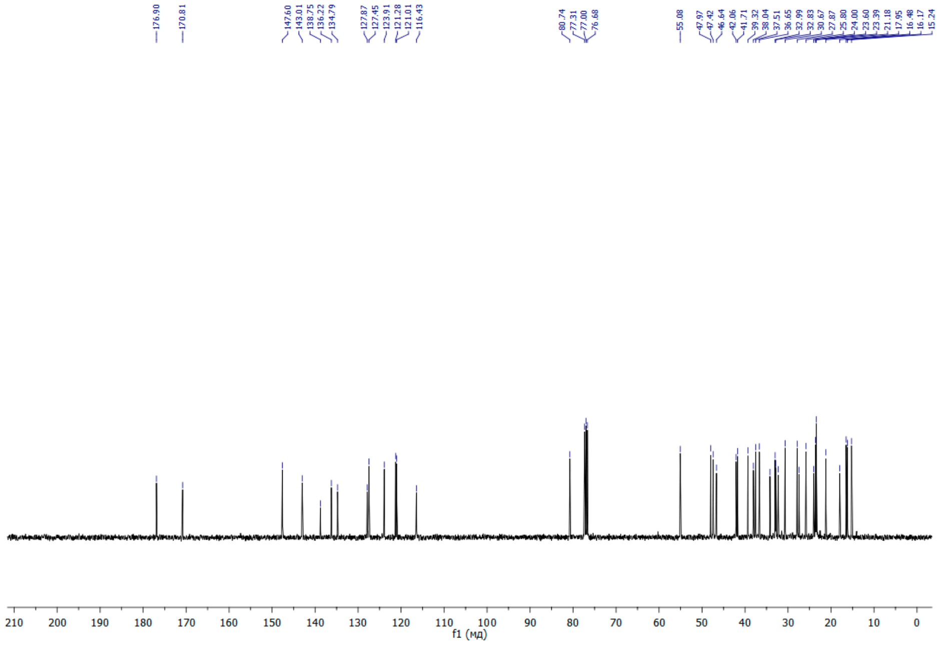

Figure S18. ^{1}H NMR Spectrum of compound **7i** (500 MHz, $CDCl_3$)

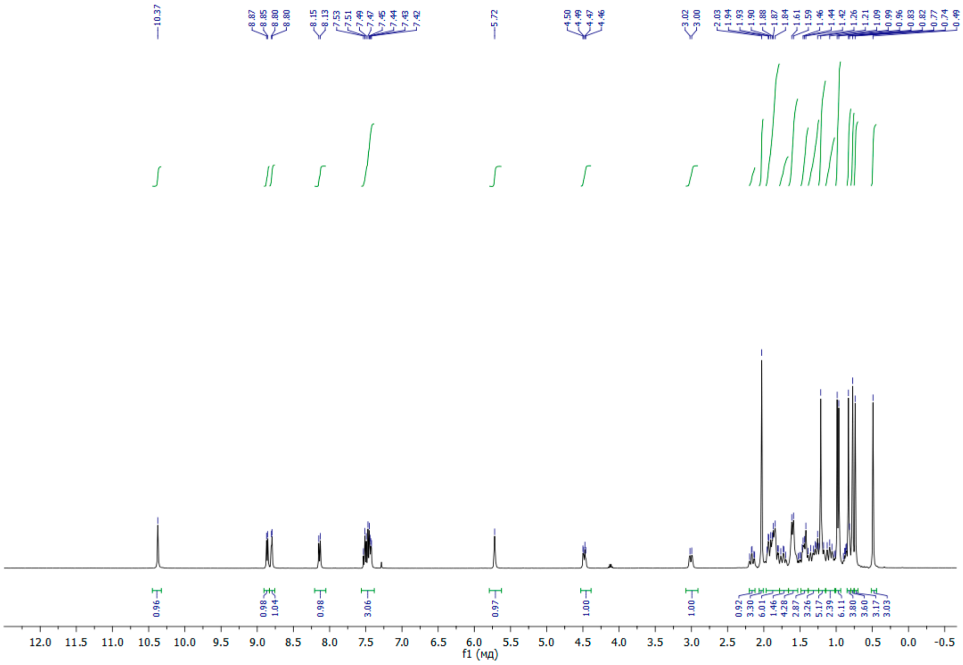

Figure S19. ^{13}C NMR Spectrum of compound **8a** (125 MHz, $CDCl_3$)

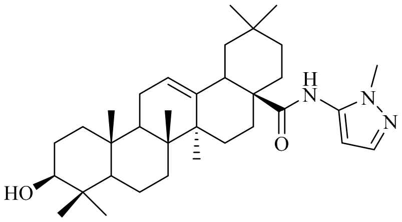

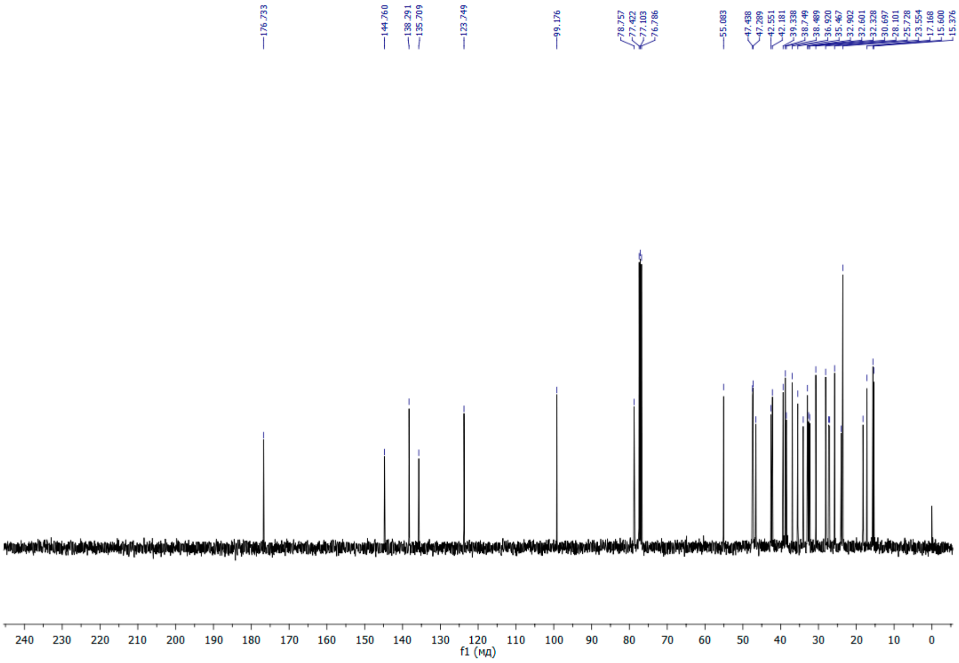

Figure S20. ^{1}H NMR Spectrum of compound **8a** (500 MHz, $CDCl_3$)

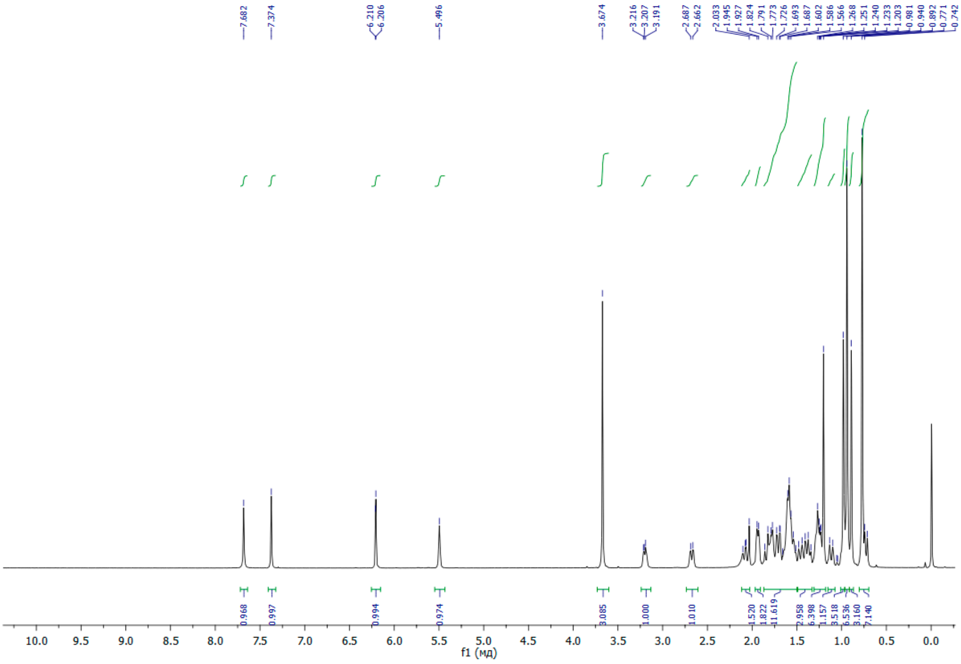

Figure S21. ^{13}C NMR Spectrum of compound **8b** (125 MHz, $CDCl_3$)

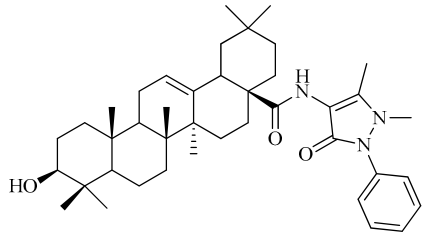

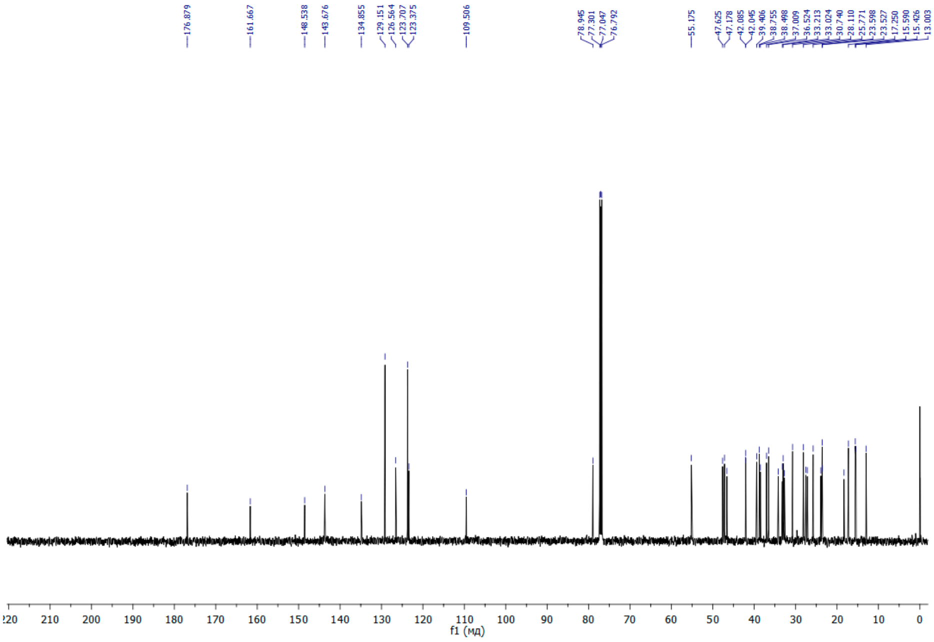

Figure S22. ^{1}H NMR Spectrum of compound **8b** (500 MHz, $CDCl_3$)

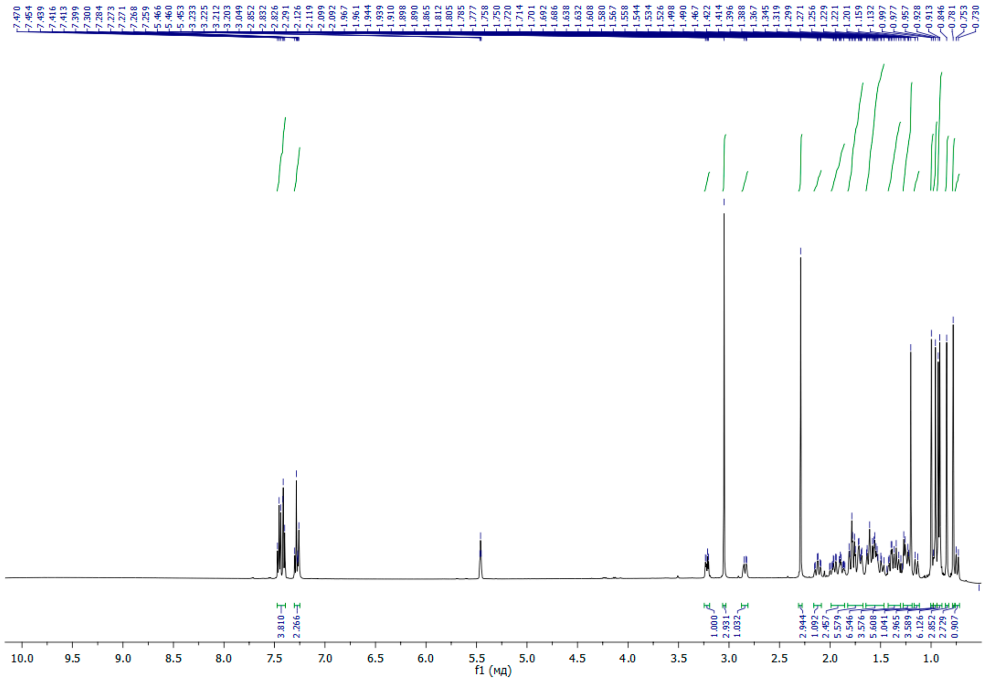

Figure S23. ^{13}C NMR Spectrum of compound **8c** (125 MHz, $CDCl_3$)

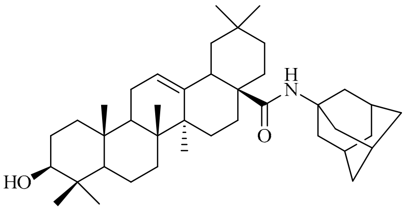

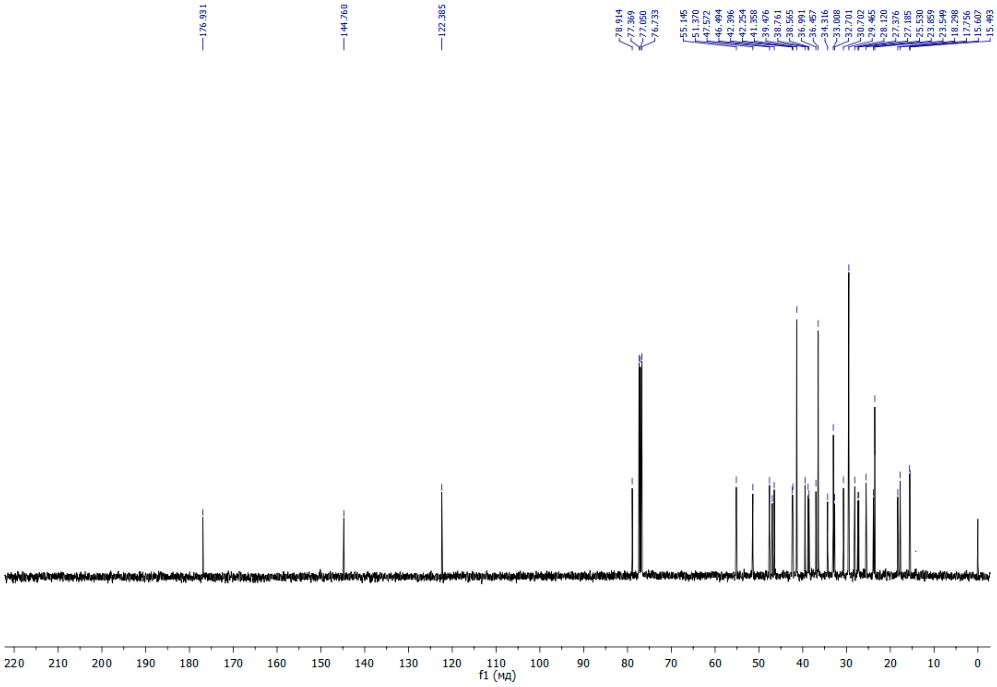

Figure S24. ^{1}H NMR Spectrum of compound **8c** (500 MHz, $CDCl_3$)

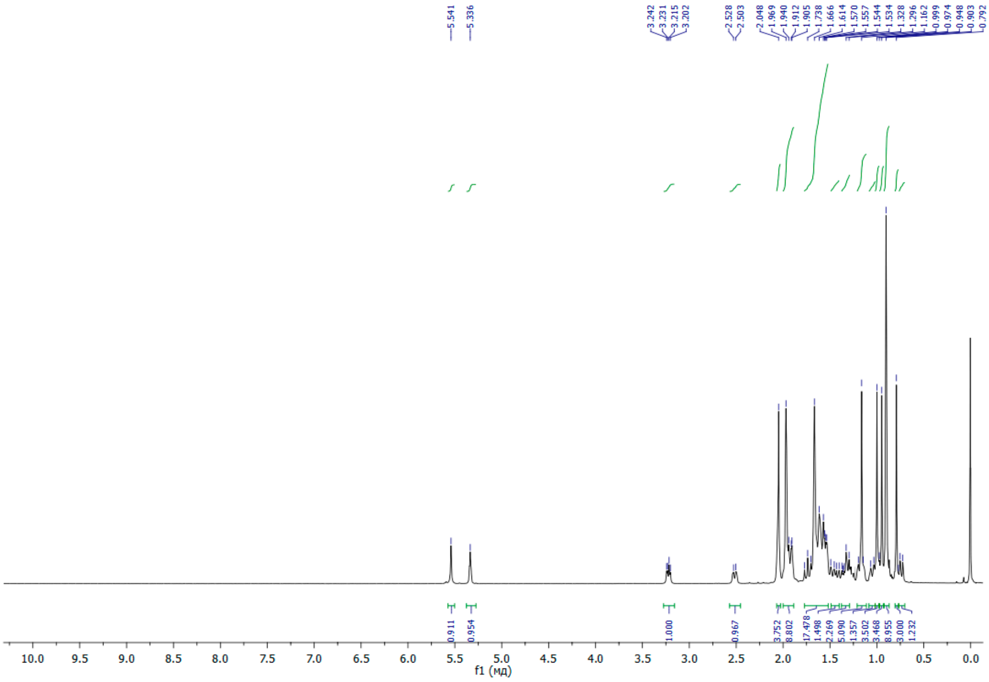

Figure S25. ^{13}C NMR Spectrum of compound **8d** (125 MHz, $CDCl_3$)

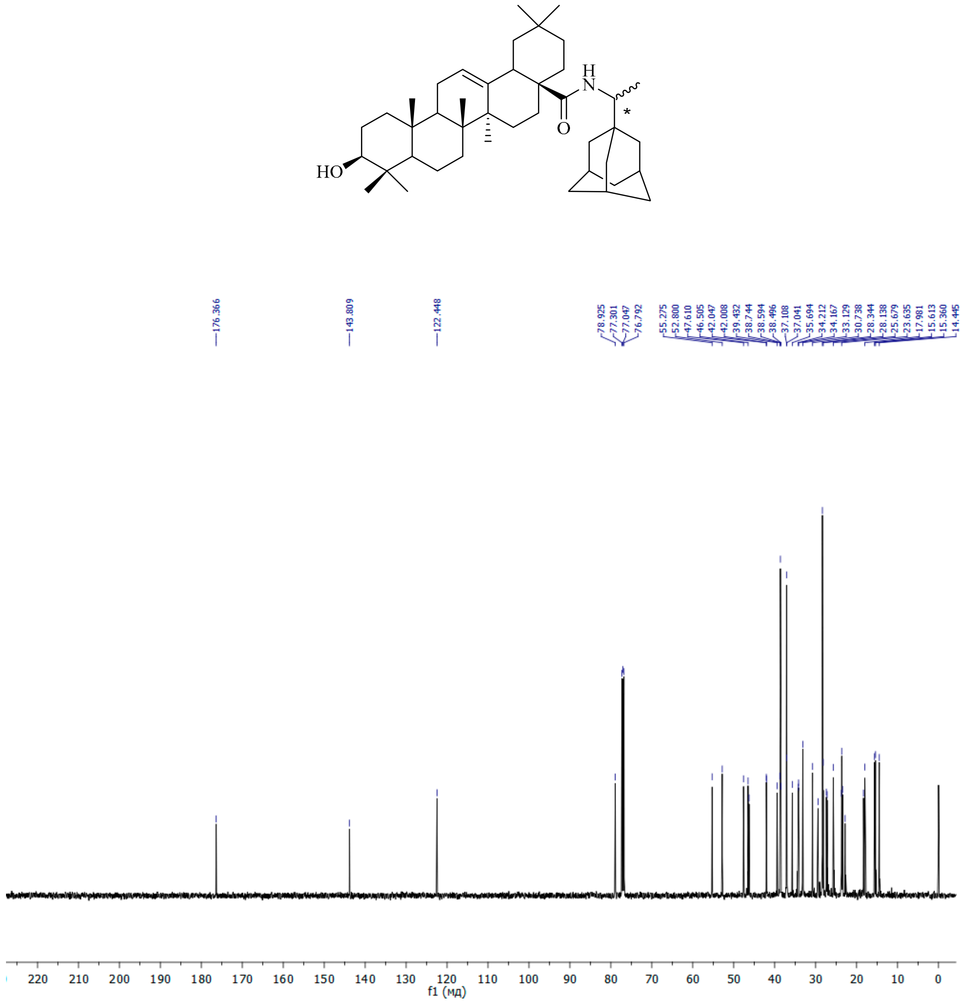

Figure S26. ^{1}H NMR Spectrum of compound **8d** (500 MHz, $CDCl_3$)

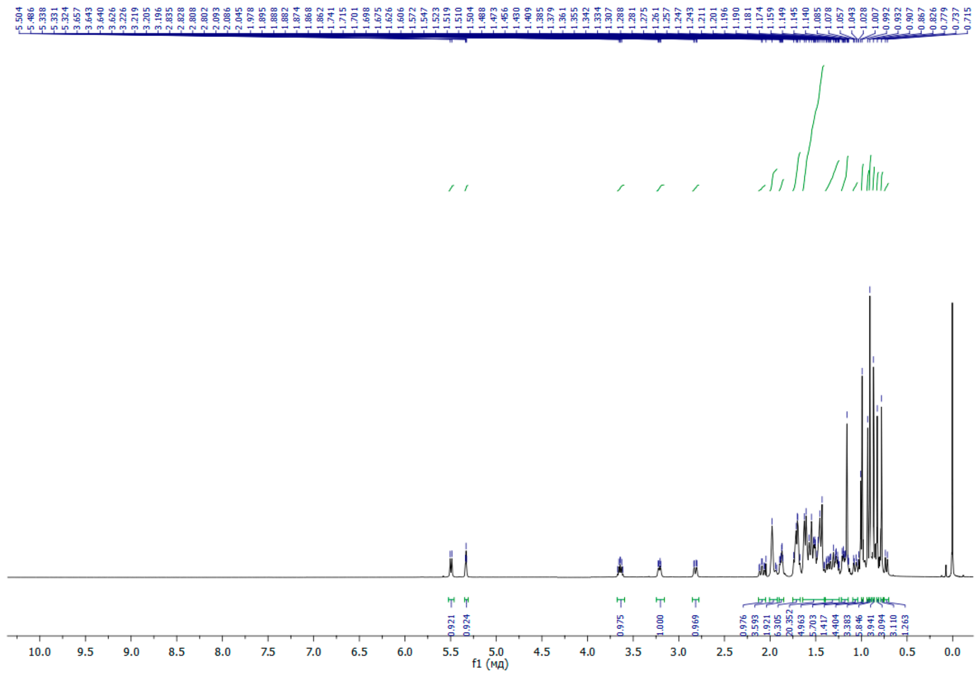

Figure S27. ^{13}C NMR Spectrum of compound **8e** (125 MHz, $CDCl_3$)

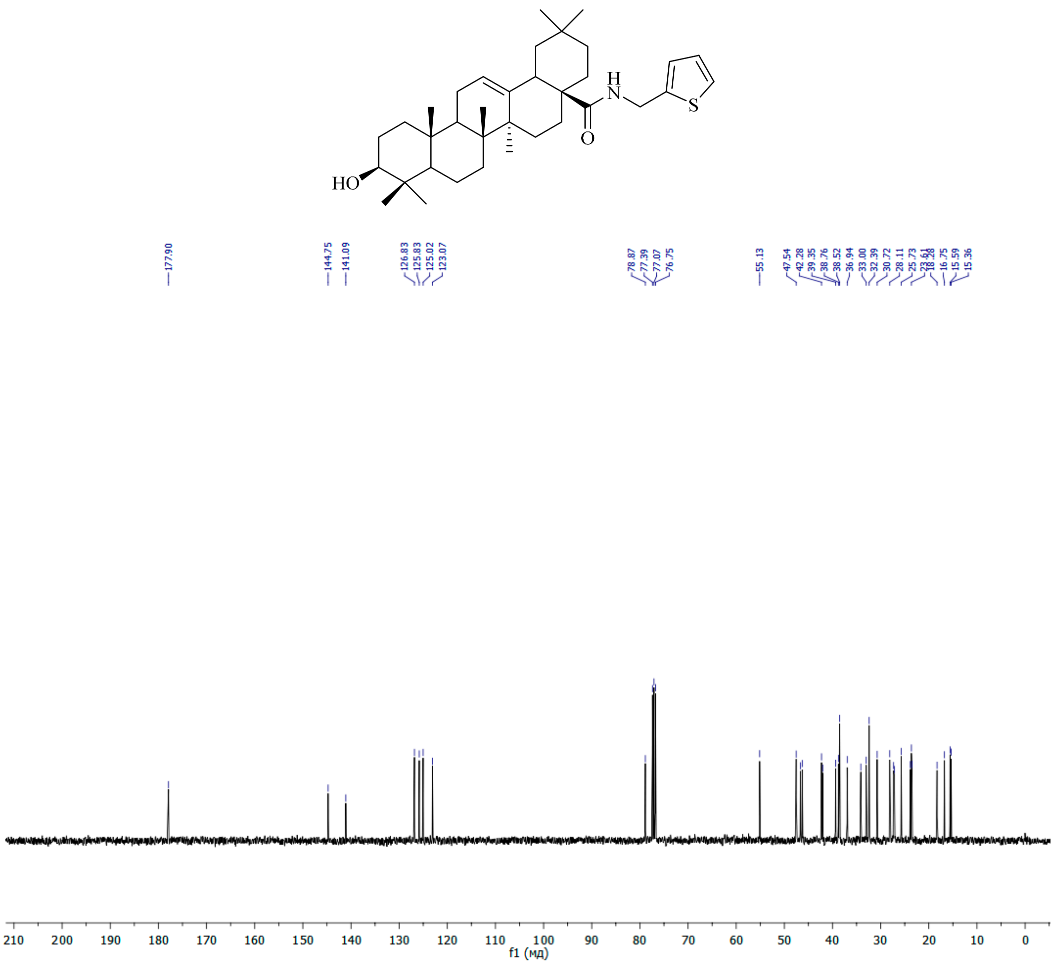

Figure S28. ^{1}H NMR Spectrum of compound **8e** (500 MHz, $CDCl_3$)

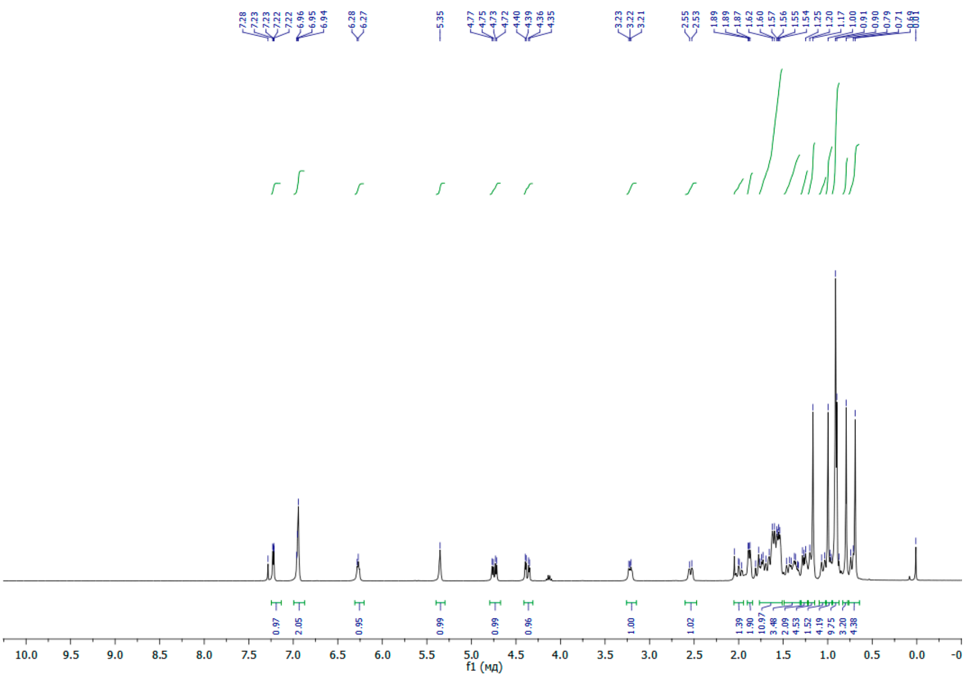

Figure S29. ^{13}C NMR Spectrum of compound **8f** (125 MHz, $CDCl_3$)

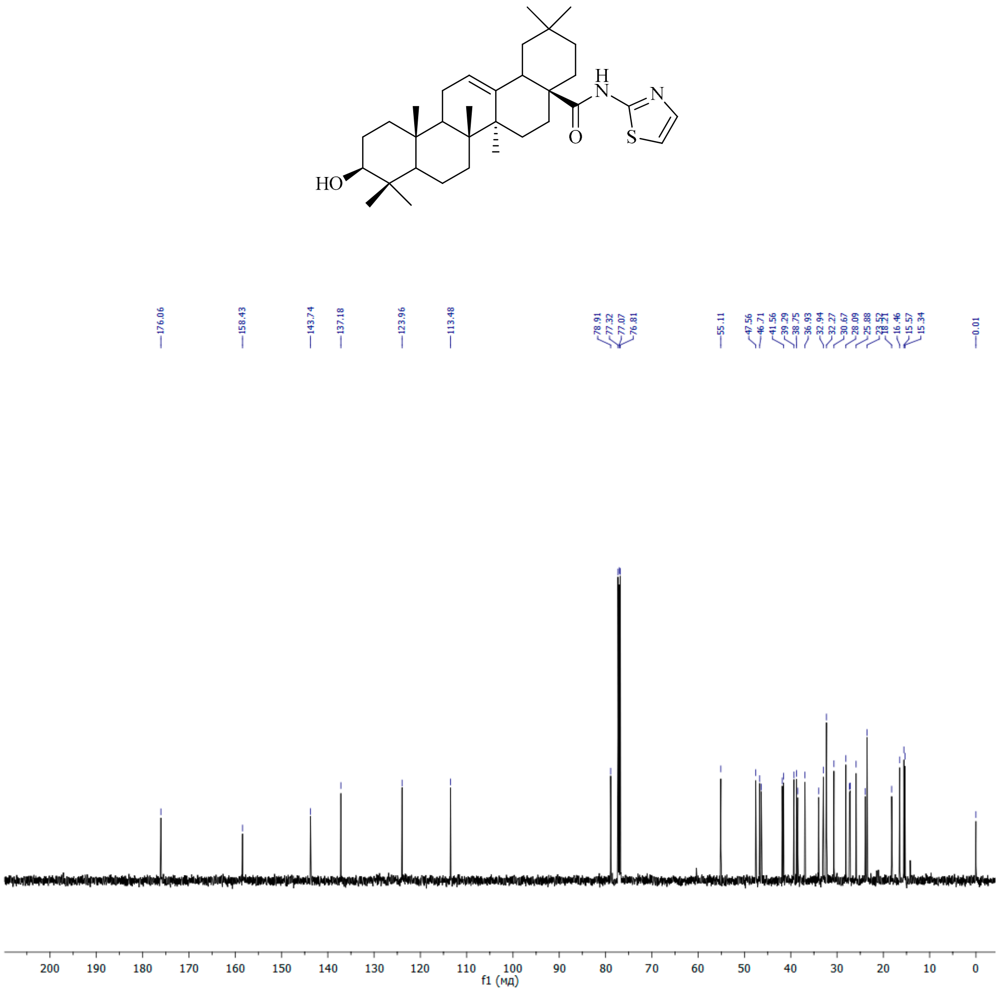

Figure S30. ^{1}H NMR Spectrum of compound **8f** (500 MHz, $CDCl_3$)

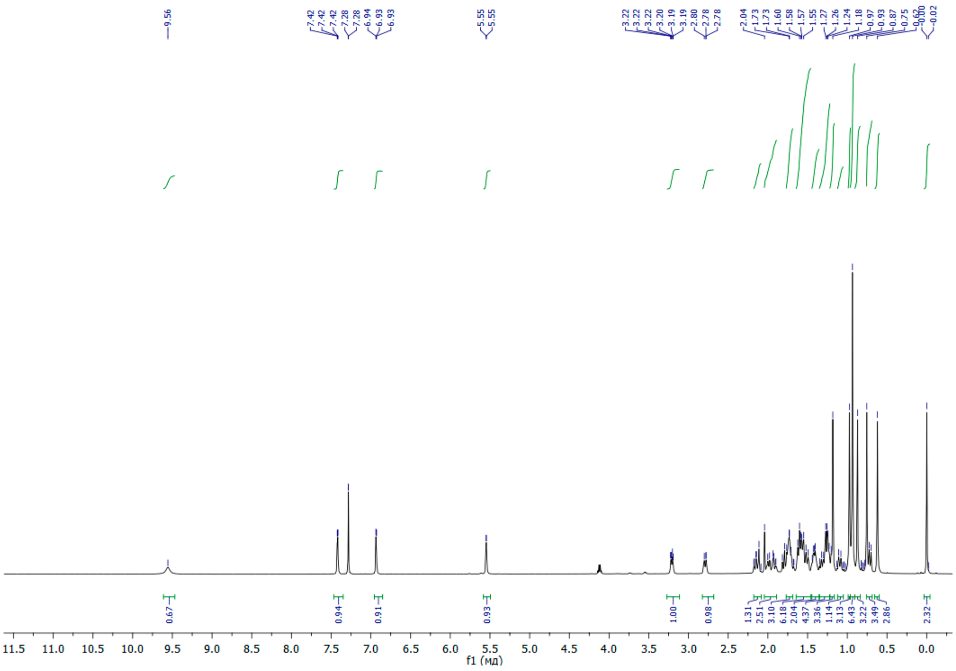

Figure S31. ^{13}C NMR Spectrum of compound **8g** (125 MHz, $CDCl_3$)

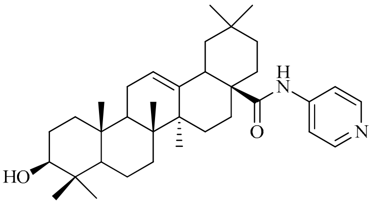

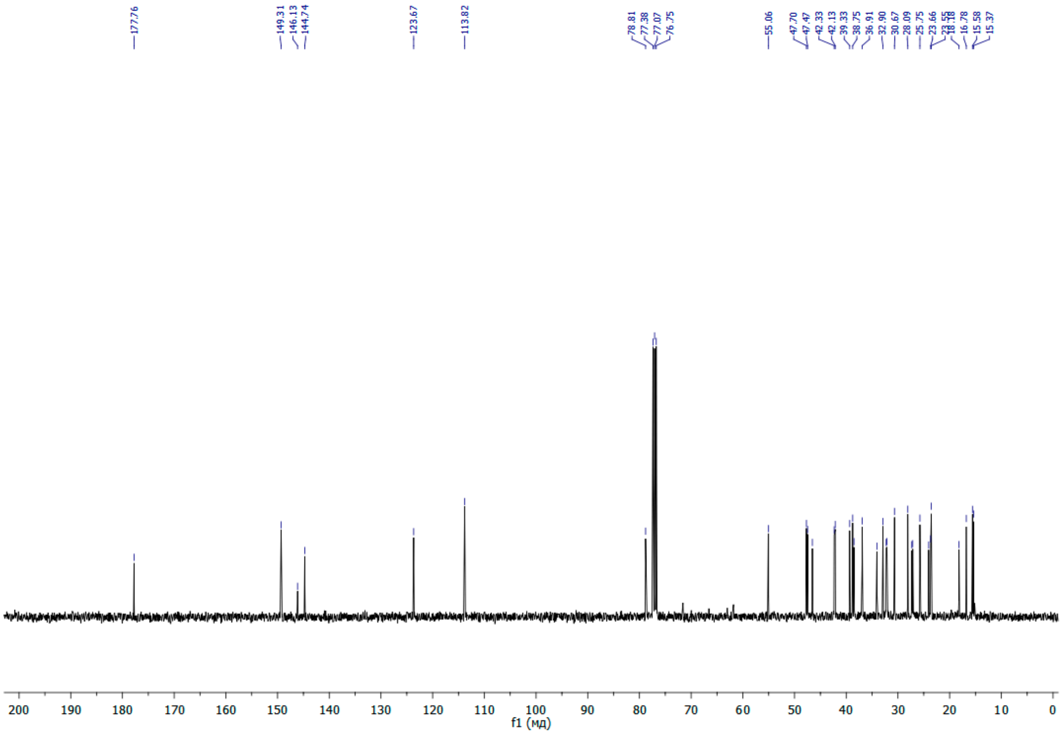

Figure S32. ^{1}H NMR Spectrum of compound **8g** (500 MHz, CDCl$_3$)

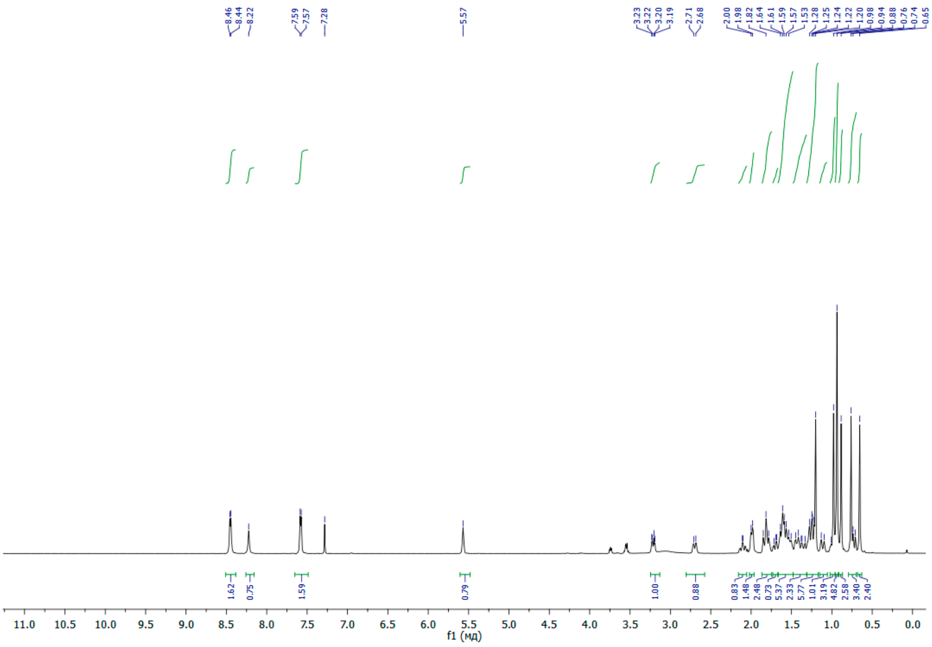

Figure S33. ^{13}C NMR Spectrum of compound **8h** (125 MHz, CDCl$_3$)

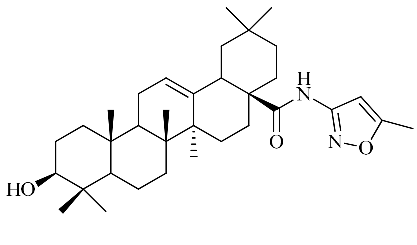

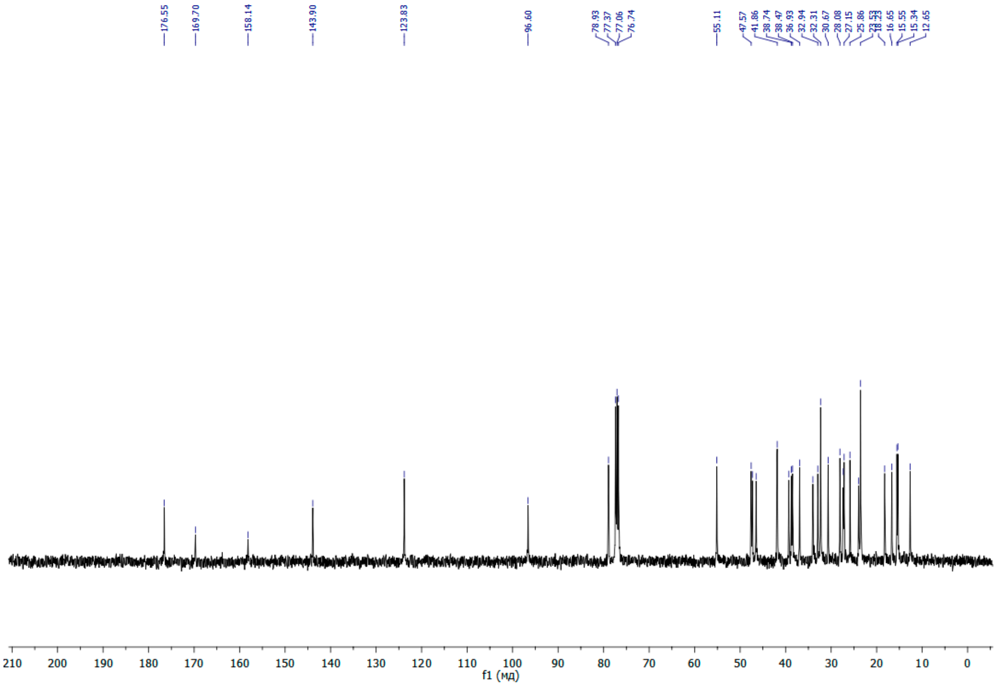

Figure S34. ^{1}H NMR Spectrum of compound **8h** (500 MHz, $CDCl_3$)

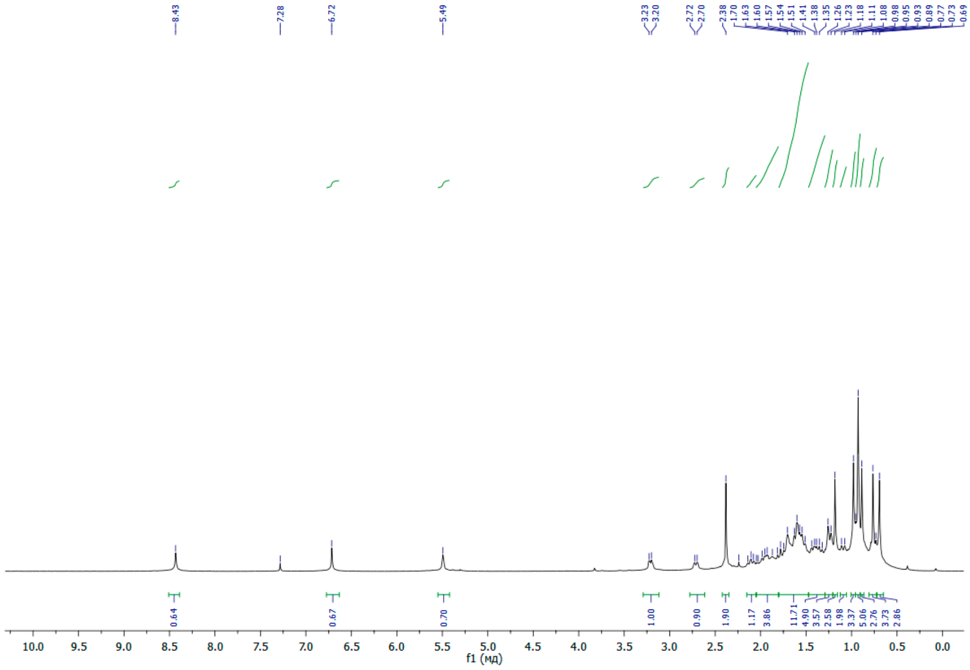

Figure S35. ^{13}C NMR Spectrum of compound **8i** (125 MHz, $CDCl_3$)

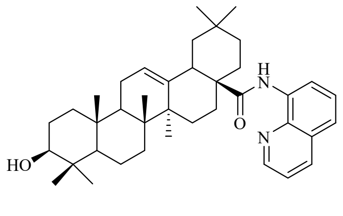

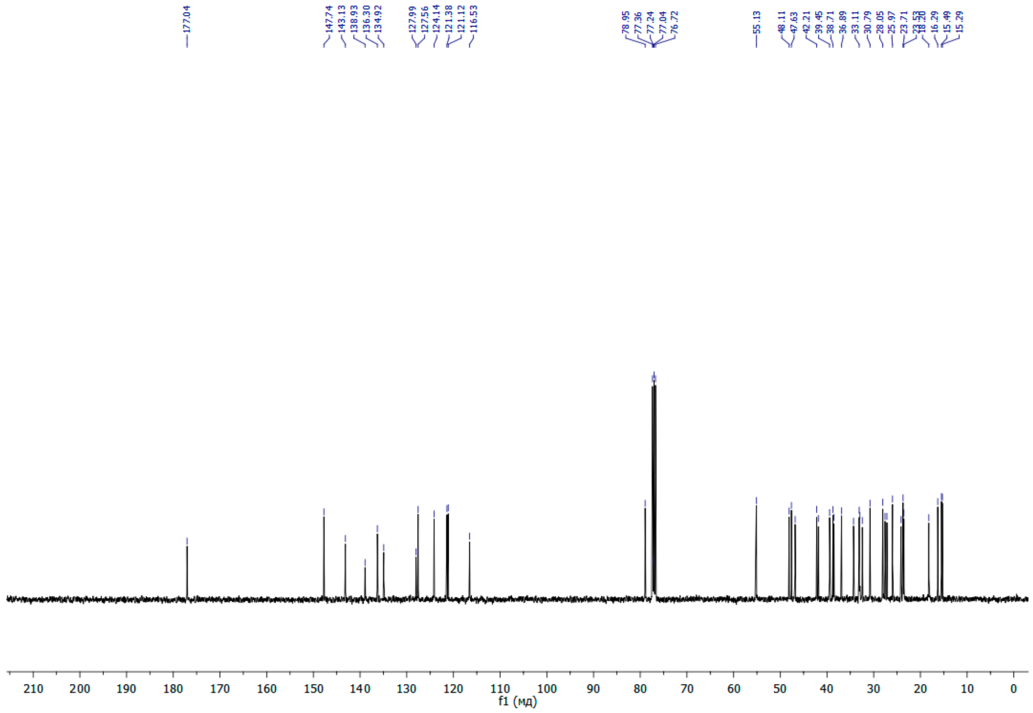

Figure S36. ^{1}H NMR Spectrum of compound **8i** (500 MHz, $CDCl_3$)

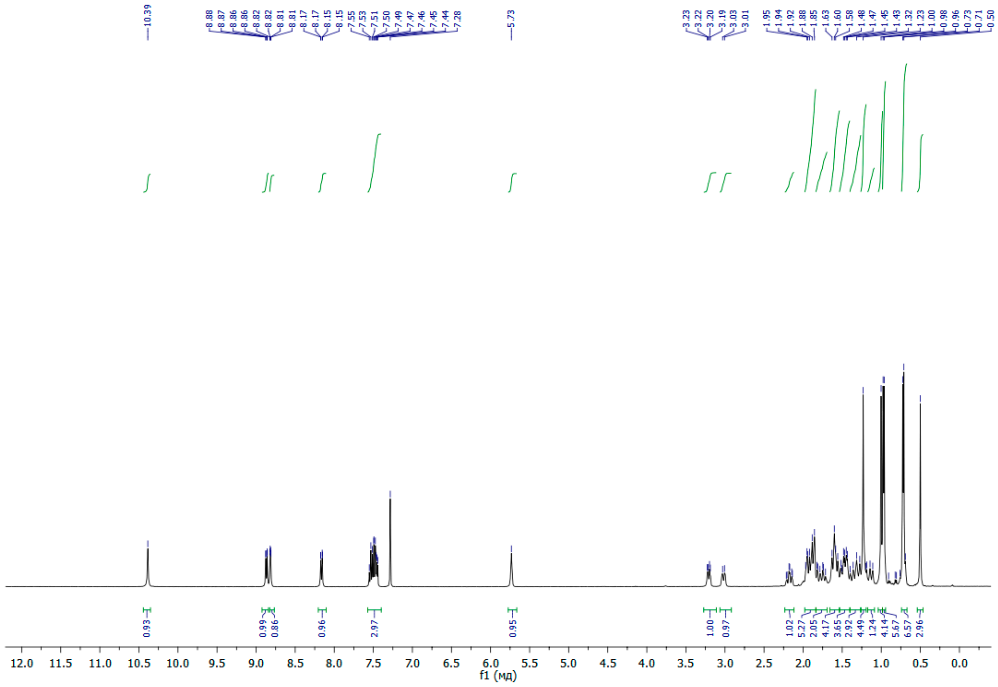

Figure S37. ^{13}C NMR Spectrum of compound **9a** (125 MHz, $CDCl_3$)

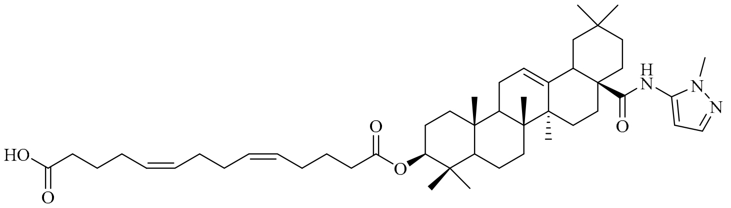

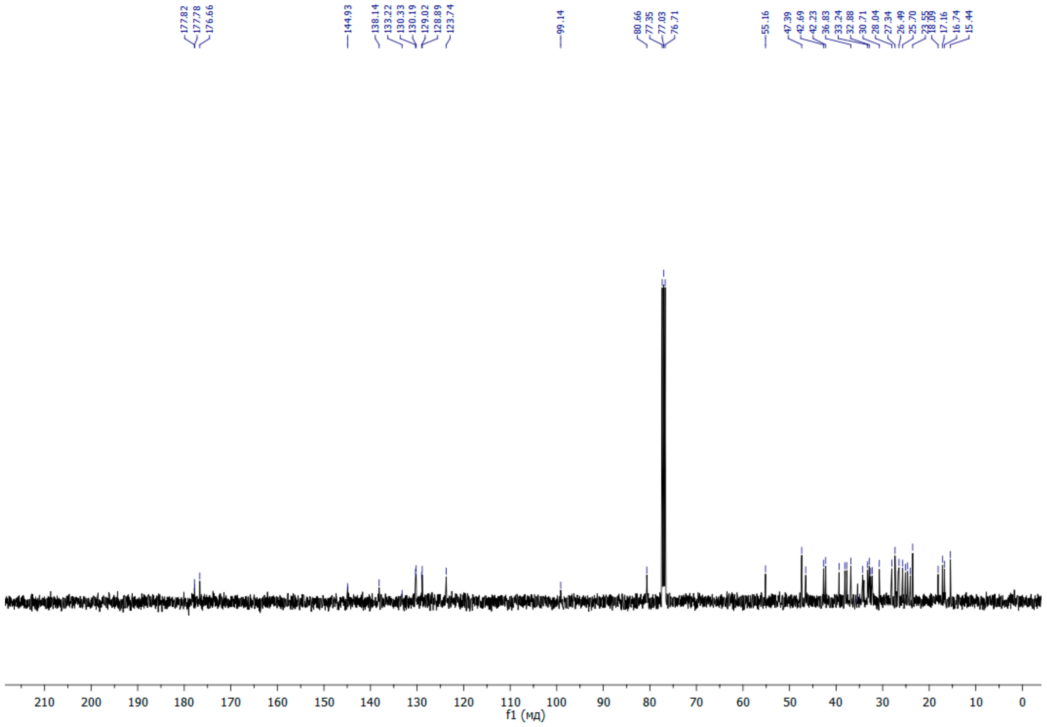

Figure S38. ^{1}H NMR Spectrum of compound **9a** (500 MHz, $CDCl_3$)

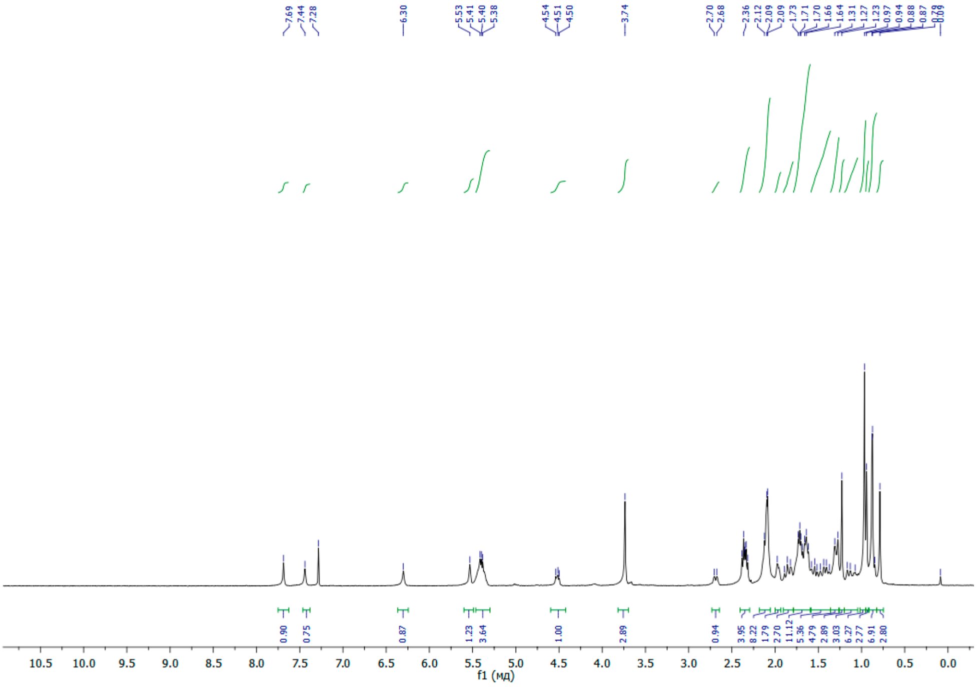

Figure S39. ^{13}C NMR Spectrum of compound **9b** (125 MHz, $CDCl_3$)

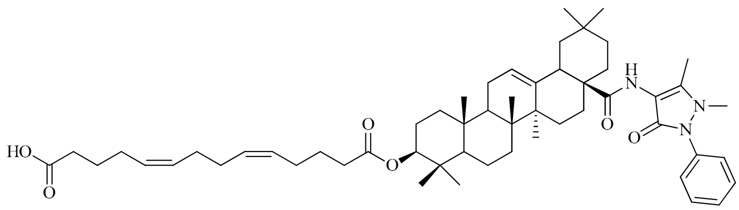

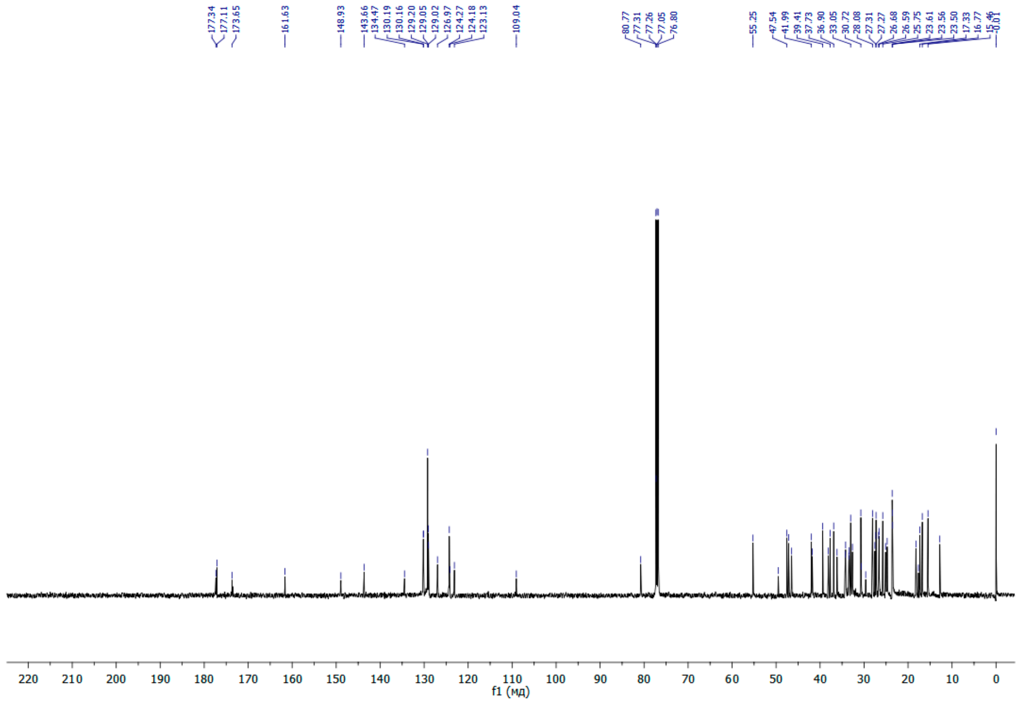

Figure S40. ^{1}H NMR Spectrum of compound **9b** (500 MHz, $CDCl_3$)

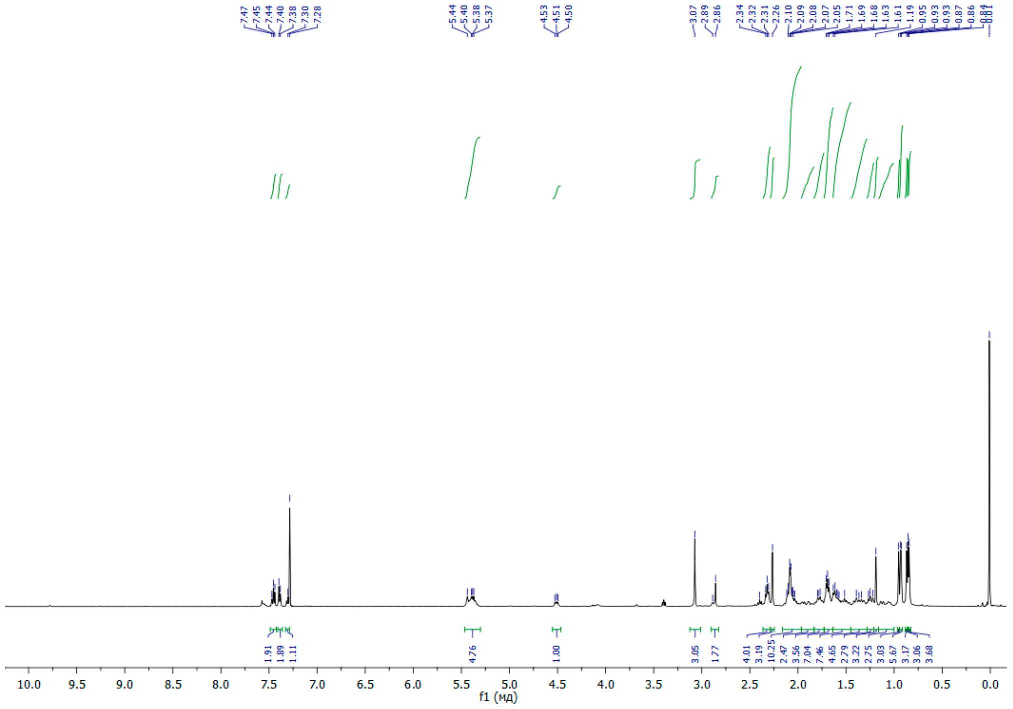

Figure S41. ^{13}C NMR Spectrum of compound **9c** (125 MHz, $CDCl_3$)

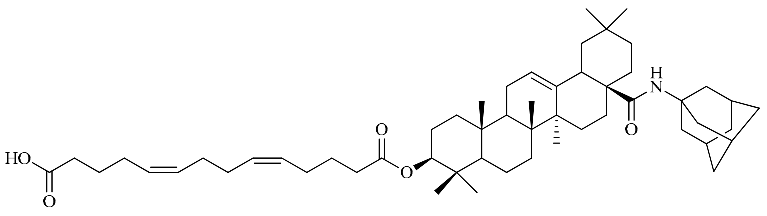

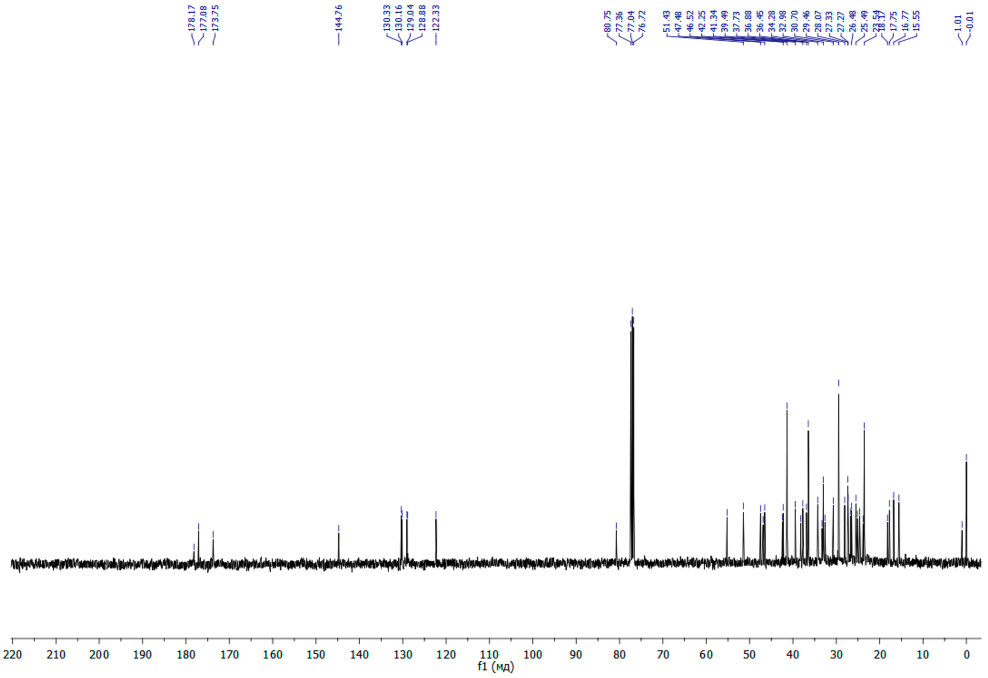

Figure S42. ^{1}H NMR Spectrum of compound **9c** (500 MHz, $CDCl_3$)

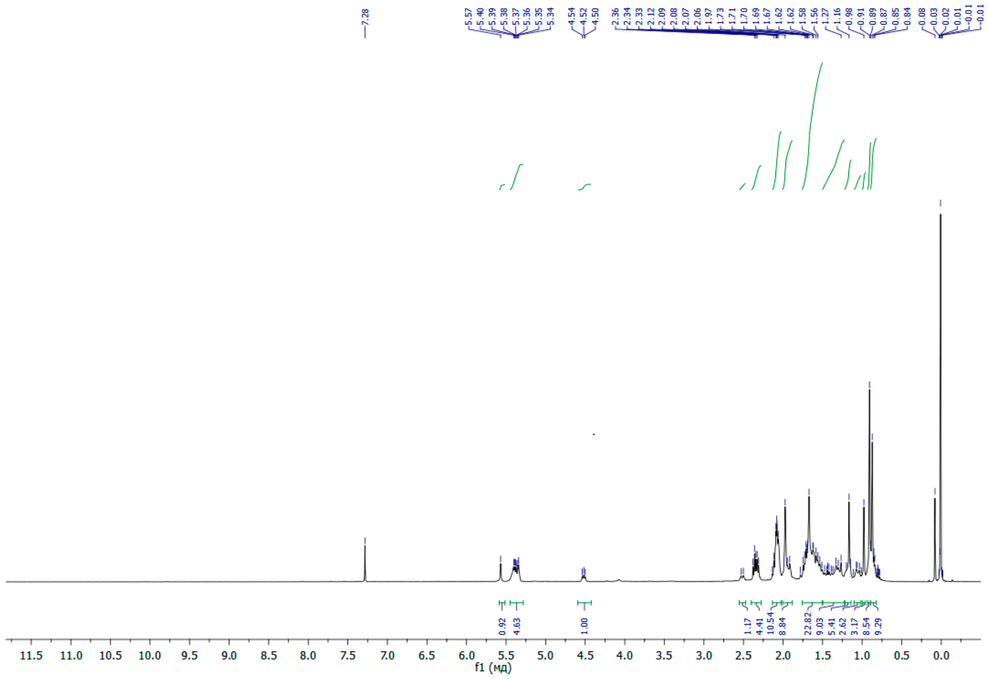

Figure S43. ^{13}C NMR Spectrum of compound **9d** (125 MHz, $CDCl_3$)

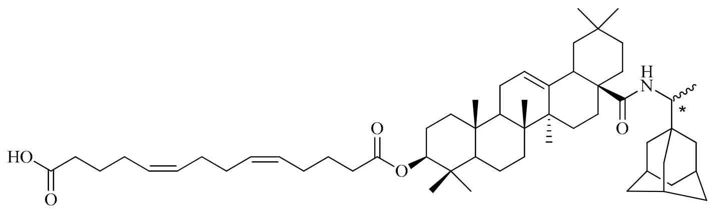

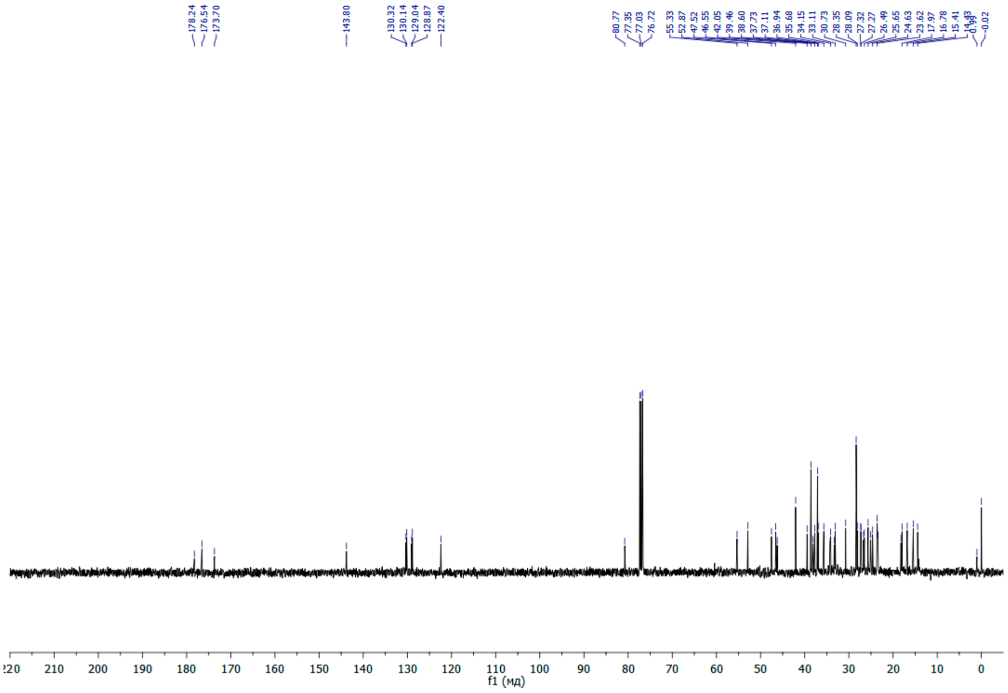

Figure S44. ^{1}H NMR Spectrum of compound **9e** (500 MHz, $CDCl_3$)

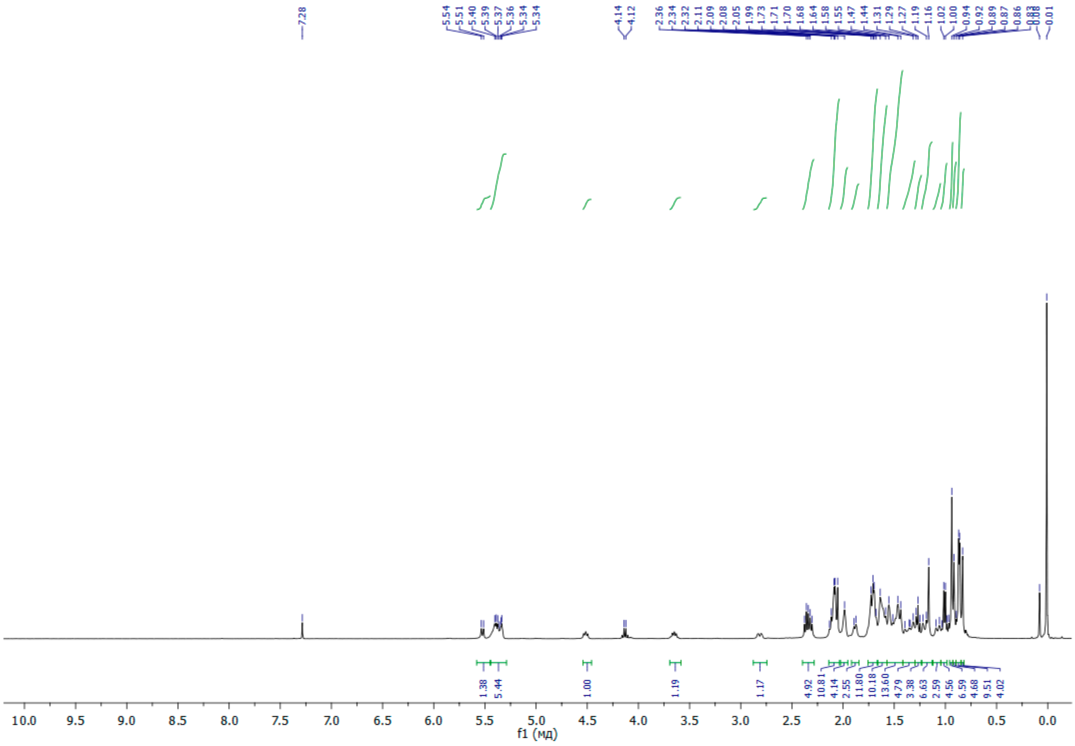

Figure S45. ^{13}C NMR Spectrum of compound **9e** (125 MHz, $CDCl_3$)

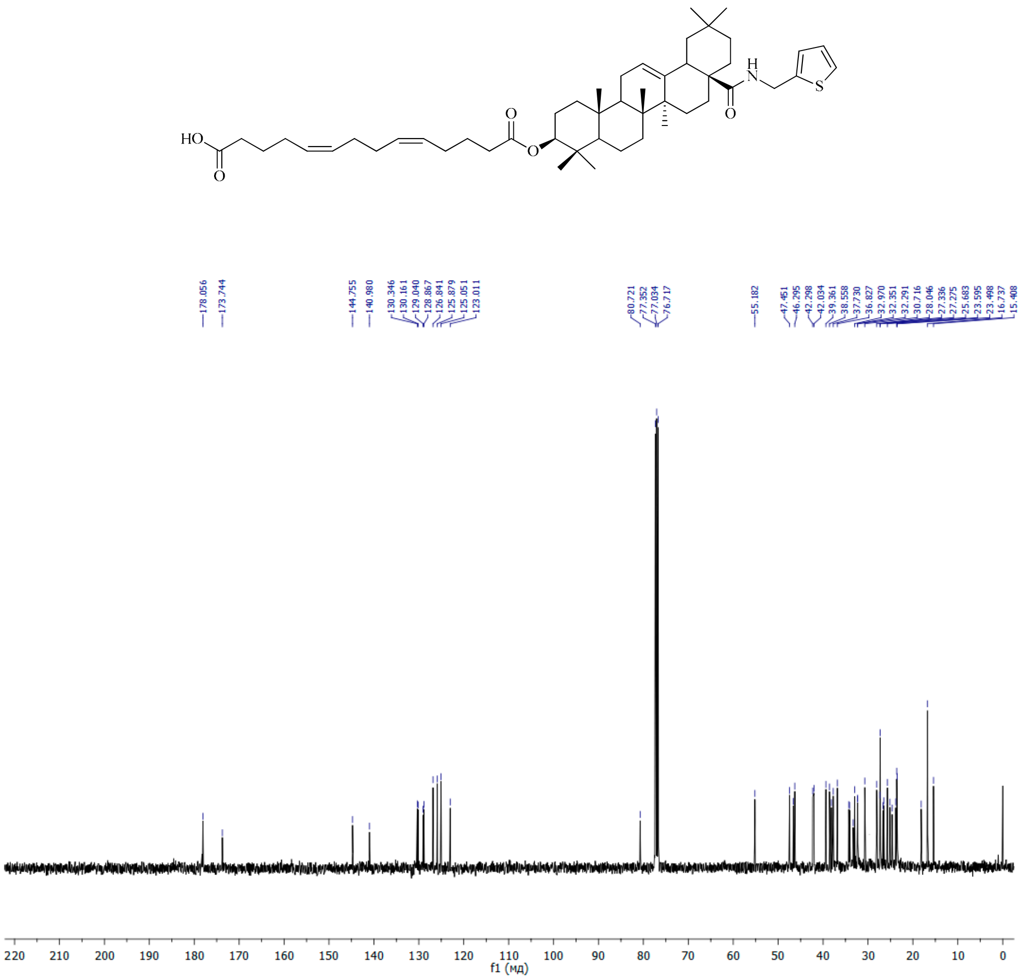

Figure S46. ^{1}H NMR Spectrum of compound **9e** (500 MHz, $CDCl_3$)

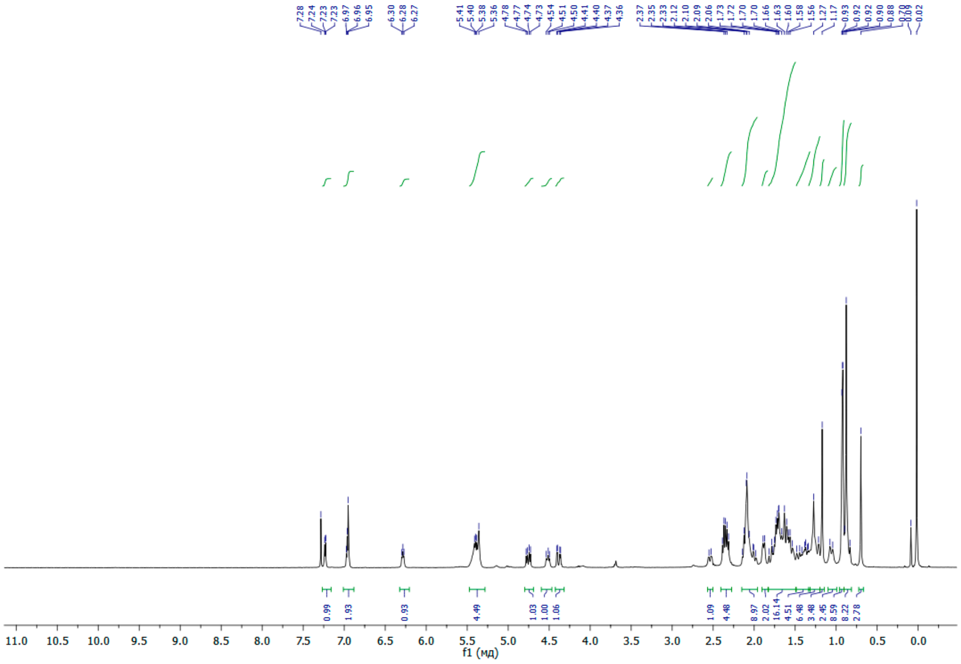

Figure S47. ^{13}C NMR Spectrum of compound **9f** (125 MHz, $CDCl_3$)

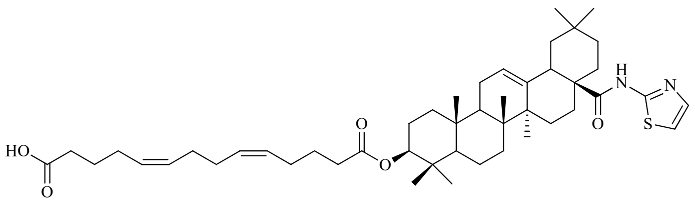

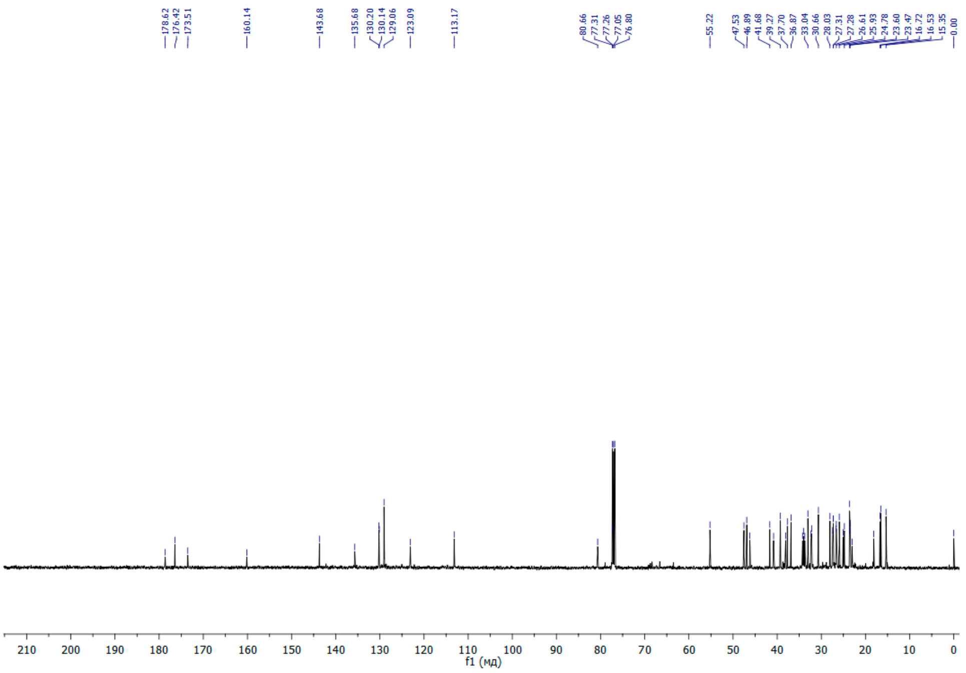

Figure S48. ^{1}H NMR Spectrum of compound **9f** (500 MHz, $CDCl_3$)

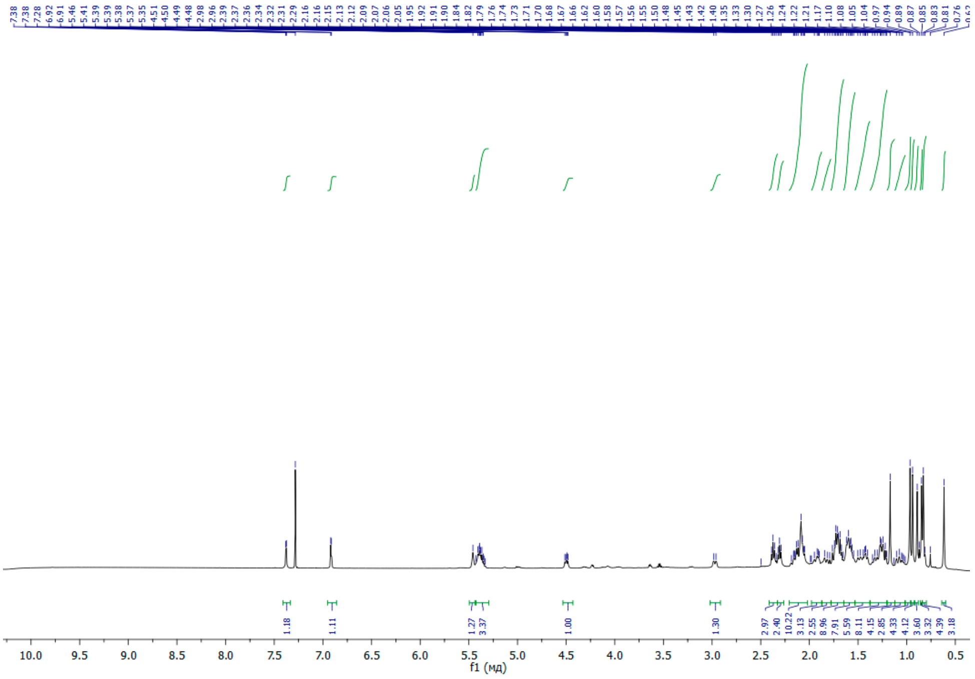

Figure S49. ^{13}C NMR Spectrum of compound **9g** (125 MHz, $CDCl_3$)

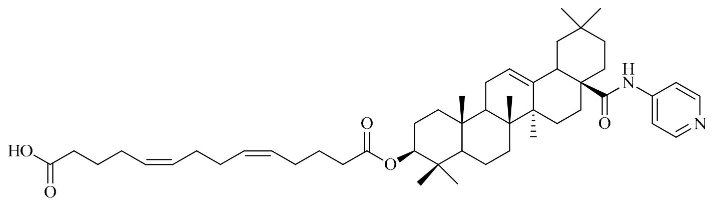

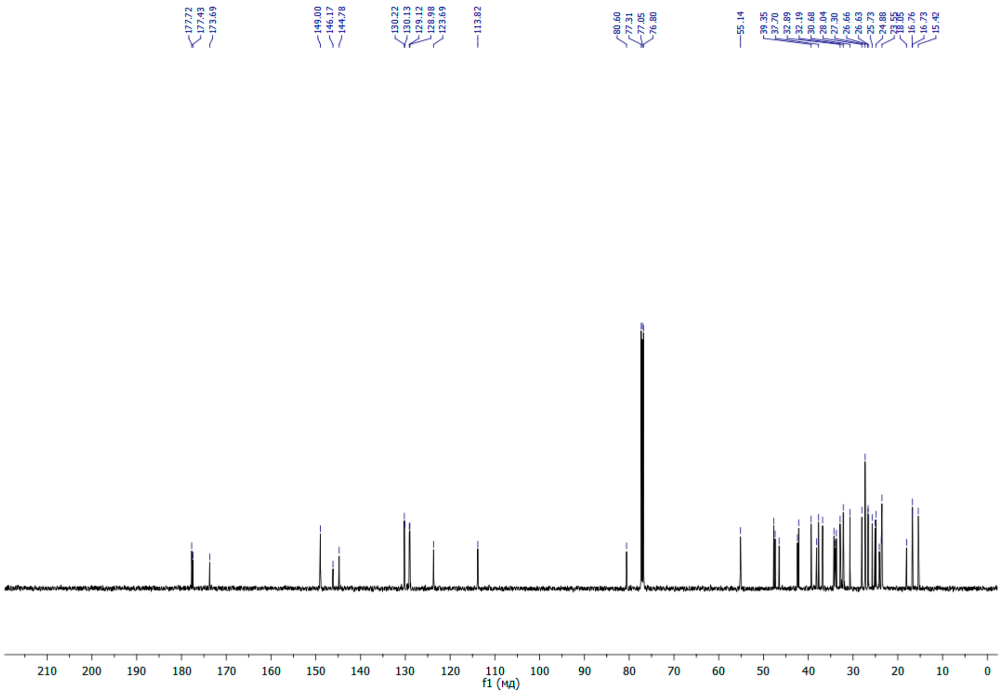

Figure S50. ^{1}H NMR Spectrum of compound **9g** (500 MHz, $CDCl_3$)

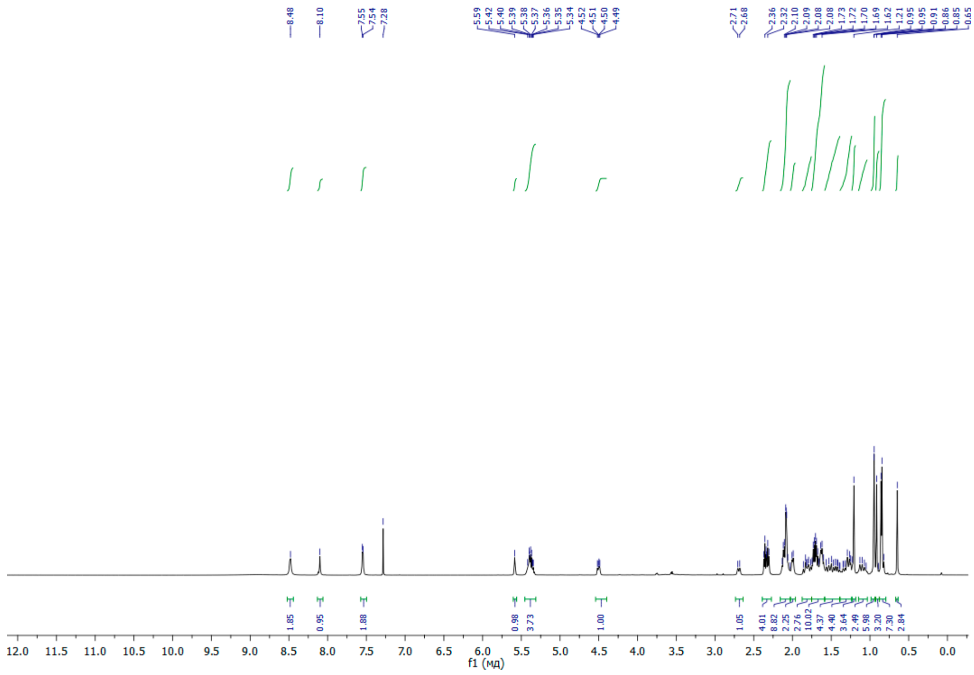

Figure S51. ^{13}C NMR Spectrum of compound **9h** (125 MHz, $CDCl_3$)

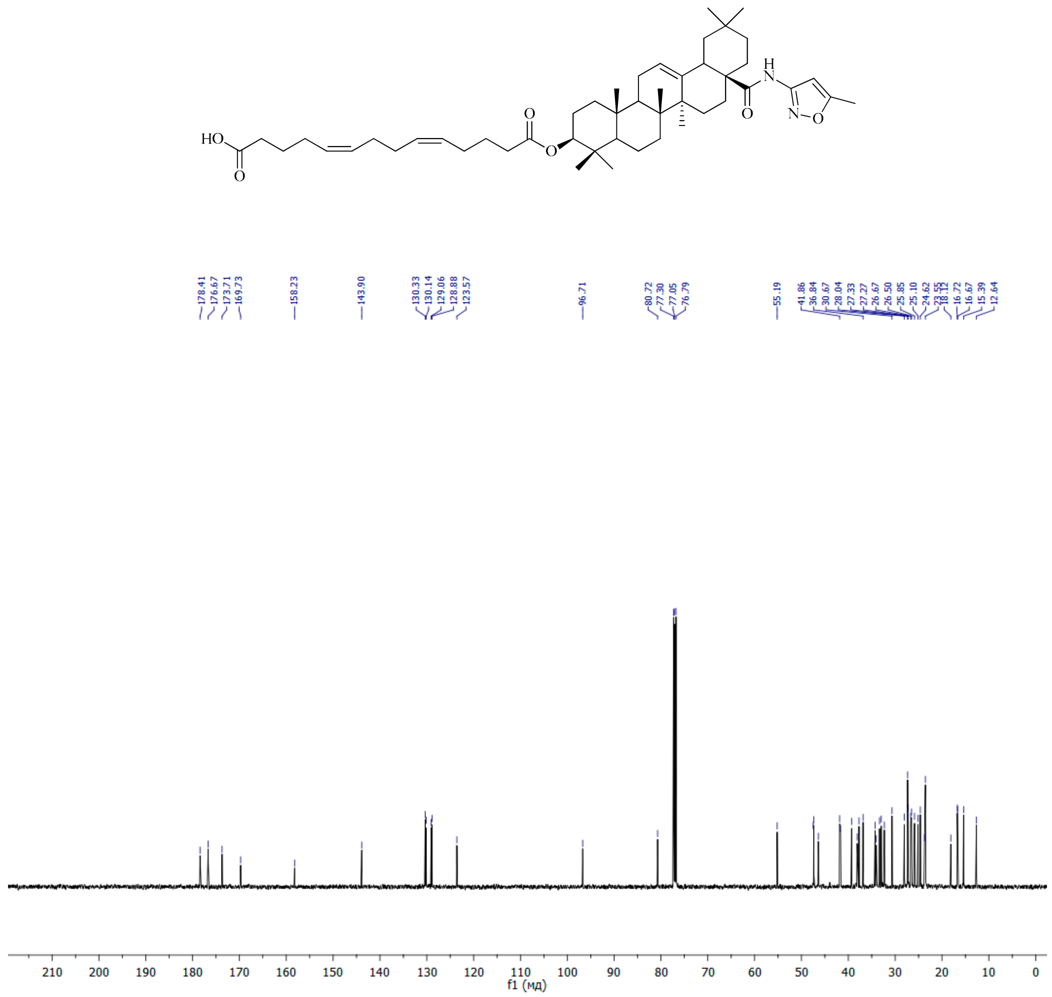

Figure S52. ^{1}H NMR Spectrum of compound **9h** (500 MHz, $CDCl_3$)

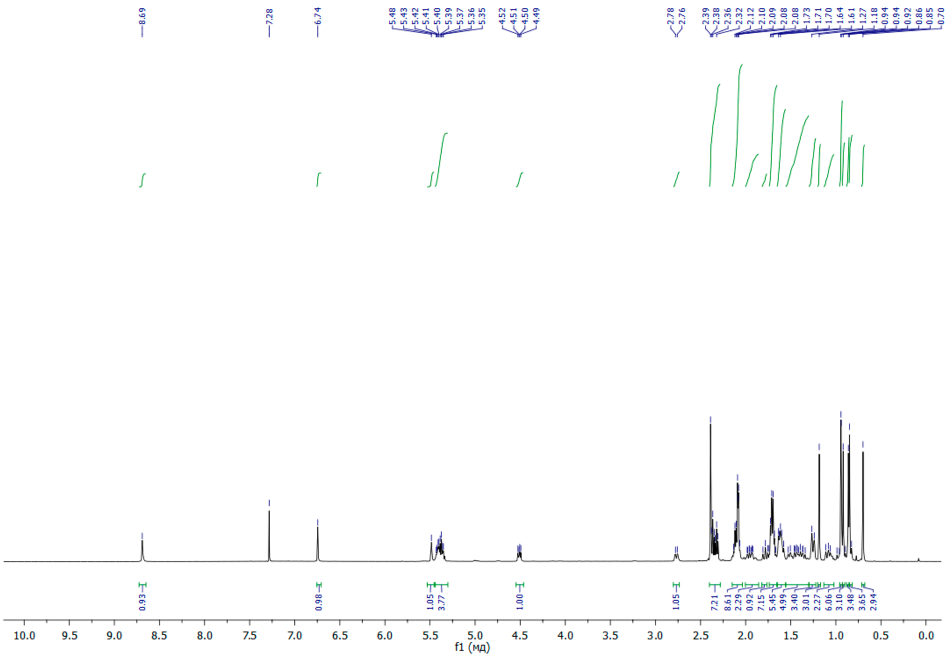

Figure S53. ^{13}C NMR Spectrum of compound **9i** (125 MHz, $CDCl_3$)

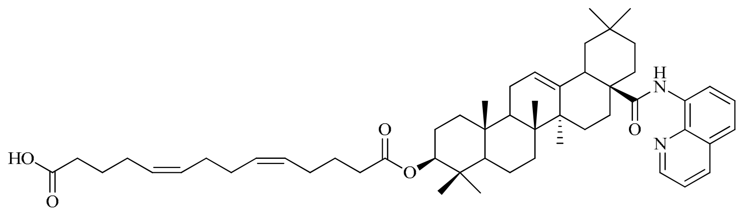

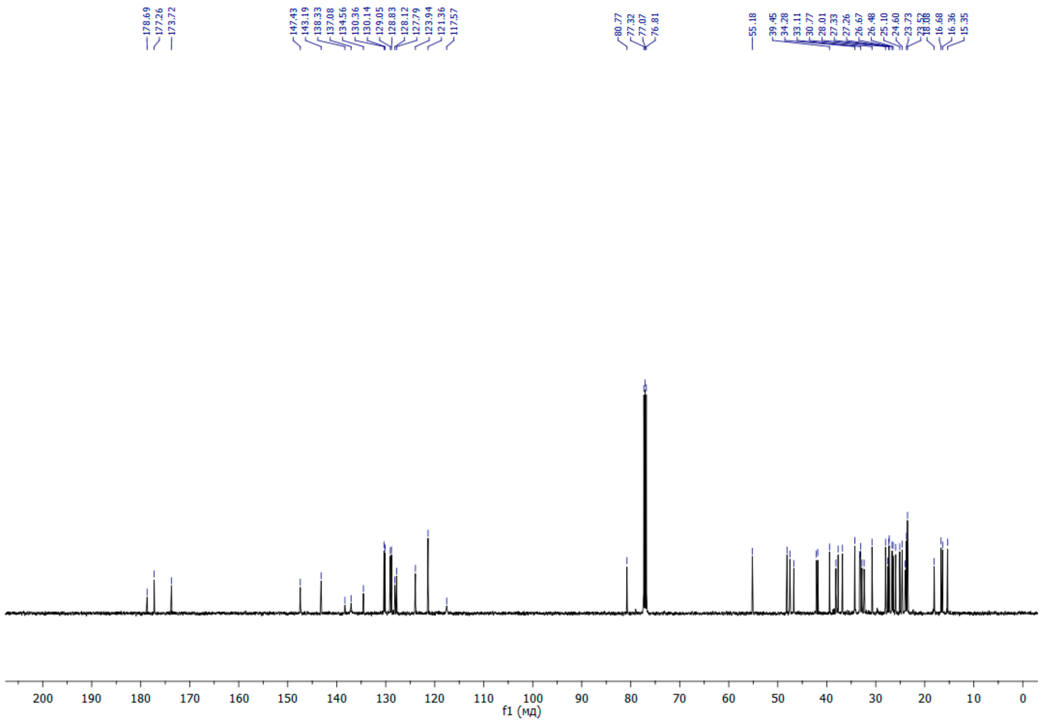

Figure S54. ^{1}H NMR Spectrum of compound **9i** (500 MHz, $CDCl_3$)

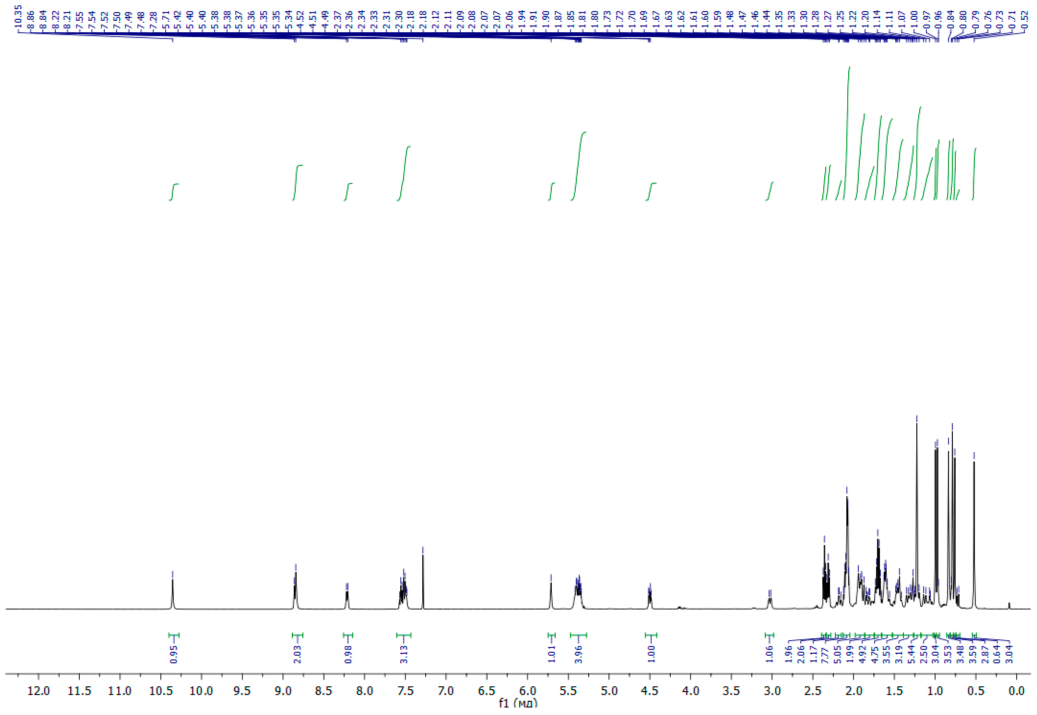

Table S1. Numerical CC_{50} values for each compound in Jurkat, K562, U937, and HEK293 cell lines.

	**Jurkat**	**K562**	**U937**	**HEK293**
**Str**	44.65±4.15	49.78±3.85	39.31±2.91	84.37±7.34
**CPT**	588.47±46.19	594.56±54.43	521.95±44.44	691.81±53.65
**Oleanoic acid**	198.53±18.42	171.31±16.47	143.76±14.32	497.34±39.76
**7a**	22.11±1.96	17.68±1.39	14.36±1.04	58.36±6.02
**7b**	86.47±8.46	81.34±7.98	72.19±7.34	218.49±20.73
**7c**	121.28±11.83	103.58±10.62	98.92±9.54	365.92±34.67
**7d**	119.64±11.76	99.91±9.38	97.13±8.96	341.64±33.84
**7e**	131.26±12.47	124.91±11.86	108.39±11.04	437.94±42.64
**7f**	151.68±14.12	143.72±12.77	132.49±12.89	493.64±42.24
**7g**	142.73±14.26	124.91±11.86	112.34±12.26	430.42±41.88
**7h**	146.37±13.95	139.28±12.94	129.13±11.67	488.59±44.76
**7i**	152.86±13.94	133.19±12.86	121.89±12.75	456.31±42.71
**8a**	23.77±2.84	20.35±2.11	19.61±1.96	109.67±10.34
**8b**	98.74±9.39	94.83±9.57	85.35±8.64	267.31±22.67
**8c**	115.42±12.06	94.73±8.68	57.65±6.49	413.76±39.54
**8d**	110.46±11.57	93.76±8.91	58.13±6.26	398.16±37.62
**8e**	164.88±14.21	149.38±12.61	121.65±11.97	593.08±53.91
**8f**	188.57±17.43	167.53±17.09	156.71±14.95	611.31±57.69
**8g**	159.73±14.26	137.13±12.42	134.61±12.82	442.86±40.31
**8h**	179.36±17.05	161.42±16.37	155.32±15.16	597.62±58.98
**8i**	164.86±14.32	154.72±12.86	142.21±12.47	514.97±40.34
**9a**	4.51±0.41	3.09±0.34	2.85±0.34	26.17±1.93
**9b**	6.11±0.56	5.76±0.69	4.86±0.42	21.37±2.33
**9c**	54.42±5.88	49.24±5.01	29.37±3.18	167.18±15.49
**9d**	49.96±4.07	47.56±4.82	31.66±3.09	178.34±16.84
**9e**	76.26±7.25	69.79±6.99	56.39±5.11	195.43±17.95
**9f**	96.69±9.18	84.35±8.47	79.73±6.78	204.64±19.67
**9g**	80.36±7.86	72.11±6.39	61.22±6.48	223.45±21.83
**9h**	91.23±8.34	81.47±7.62	72.26±6.85	212.93±18.28
**9i**	84.67±8.21	77.29±6.85	62.87±5.91	261.74±22.67

Table S2: Changes in genotoxicity-related protein levels (% of control) in Jurkat cells upon exposure to compound **9a**

	**Chk2**	**MDM2**	**H2A.X**	**p21**	**ATR**	**p53**	**Chk1**
**Jurkat unstim**	100	100	100	100	100	100	100
**9a-CC$_{50}$ 6h**	936	102	3173	390	131	128	144
**9a-0,5CC$_{5}$0 6h**	645	115	2485	318	119	52	97
**Anisomycin**	20836	367	1841	3727	440	738	610
**Camptotecin**	5800	1417	957	170654	263	17285	52

Table S3: Changes in apoptosis-related protein levels (% of control) in Jurkat cells upon exposure to compound **9a**

	**Bad**	**Casp 8**	**Bcl-2**	**Casp9**	**JNK**	**p53**	**Akt**
**Jurkat unstim**	100	100	100	100	100	100	100
**9a-CC$_{50}$ 6h**	55	17	20	1341	106	160	8,8
**9a-0,5CC$_{50}$ 6h**	106	2,4	140	1144	78	112	15
**Anisomycin**	486	1229	3180	9353	357	17	129
**Camptotecin**	516	209	580	10289	141	132	196
